# The epidemiology of maternal mental health in Africa: a systematic review

**DOI:** 10.1007/s00737-025-01563-4

**Published:** 2025-04-12

**Authors:** Amanuel Abajobir, Estelle Monique Sidze, Caroline Wainaina, Mulusew J. Gerbaba, Frederick Murunga Wekesah

**Affiliations:** 1 African Population and Health Research Center, Nairobi, Kenya; 2 School of Public Health, The University of Queensland, Herston, Brisbane, QLD Australia; 3 Julius Global Health, Julius Center for Health Sciences and Primary Care, University Medical Center Utrecht, Utrecht University, Utrecht, the Netherlands

**Keywords:** Maternal, Perinatal, Newborn and child health outcomes, Mental health, Mental disorders, Systematic review, Africa

## Abstract

**Background:**

Despite a growing body of evidence on maternal mental health in Africa, significant gaps remain in understanding its overall landscape, risk factors/determinants, immediate and long-term effects, accessibility to healthcare and services, and availability of practicable/effective interventions. This paper provides a thorough review of both peer-reviewed and grey literature and makes key recommendations and directions for future research and development.

**Methods:**

We systematically reviewed extant evidence using the Preferred Reporting Items for Systematic Reviews and Meta-analyses (PRISMA) 2020 guidelines. Peer-reviewed studies published in English between 2010, and June 2024 were included based on a priori criteria. The National Institutes of Health (NIH) and Critical Appraisal Skill Program (CASP) quality assessment tools were used to critically appraise the reliability, validity and overall quality of included articles. A qualitative narrative synthesis was perfomed to summarize the findings effectively.

**Results:**

A total of 206 full-text articles evaluated for eligibility and inclusion in the systematic review, predominantly observational studies with a minority employing randomized controlled trial (RCT) designs, were included, with 70%, 22%, and 8% of the articles rated as good, fair, and poor quality, respectively. Women in Africa experience a wide range of common perinatal mental disorders, including major depressive disorders and psychosis, either discretely or comorbid. Socioeconomic disadvantages and other intertwined poverty-related factors at the individual, family, social, and environmental levels are implicated in maternal mental health disorders. Currently, there is insufficient evidence regarding the short- and long-term health, development, and social impacts of maternal mental health. Addtionally, there is limited knowledge about the availability and accessibility of mental healthcare, evidence-based context-specific interventions, and healthcare-seeking behaviors of women in Africa, as well as their access to and utilization of mental health services.

**Conclusion:**

The evidence base on maternal mental health in Africa suffers from considerable variability, inconsistency, and equivocal findings resulting from heterogeneity across the studies. This restricts generalizability and the ability to draw valid conclusions. Published studies also likely underestimate the scale and health impacts of perinatal mental disorders. Evidence from these studies are rarely used to inform policies and programs. The maternal mental health ecosystem in Africa needs to be better understood. More rigorous study designs should be implemented to focus on evidence generation and the evaluation of interventions, alongside robust integration of mental health services within health systems. Policy initiatives aimed at reducing socioeconomic disparities in maternal, newborn, and child health, particularly concerning maternal mental health, must be supported by these studies.

**Article Highlights:**

• Women across Africa suffer from various mental health problems, including major depressive disorders, anxiety, and psychosis, occurring separately or in combination.

• The evidence base on maternal mental health in Africa displays significant variability, inconsistency, and ambiguous findings, largely attributed to study heterogeneity.

• Factors at the individual, familial, societal, and environmental levels contribute to poverty-related issues that can lead to or worsen maternal mental health disorders.

• Current evidence has not been synthesized to improve our understanding of the short- and long-term health impacts, developmental consequences, and social implications of maternal mental health conditions, as well as the healthcare-seeking behaviors and access to mental health services.

• Insufficient policy prioritization and funding for maternal mental health in Africa hinder the development, evaluation, and sustainability of interventions.

• There is an urgent need to integrate mental health services into primary healthcare, particularly in resource-limited settings across Africa. This integration should be guided by evidence from rigorous research that uses longitudinal designs. It is also essential to emphasize the importance of investing in digital and community-based approaches to improve the accessibility to mental health services.

**Supplementary Information:**

The online version contains supplementary material available at 10.1007/s00737-025-01563-4.

## Background

Maternal mental health broadly refers to "a state of well-being in which the individual realizes his or her own abilities, can cope with the normal stresses of life, can work productively and fruitfully, and is able to make a contribution to his or her community (WHO 2004).” Risk factors for maternal mental various dimensions, including social (social support, history of mental health issues, and life stressors), psychological (self-efficacy, optimism, resilience, and hope), and environmental constructs, influencing a woman’s ability to cope with challenges and maintain mental well-being (WHO 2004; Herrman et al. 2006; WHO 2019). Perinatal mental health disorders are *bio-psycho-social* illnesses that distort feeling, thinking, perception and respective activities, and include common mental disorders (e.g., depression, anxiety, somatic disorders, maternal dysthymia (low mood persisting for two years or more), pregnancy and postpartum obsessive compulsive disorders and birth-related posttraumatic stress disorders, as well as severe forms of mental disorders (e.g., major depressive disorder, severe anxiety and postpartum psychosis) that affect women during pregnancy or in the first year following childbirth (McNab et al. 2022; GBD 2019 Mental Disorders Collaborators 2022; González-Guarda and Ortega 2018). About 2 in 13 women of reproductive age (15–49 years) in low- and middle-income countries (LMICs), particularly those in Africa are affected by these common mental health disorders/illnesses. The disease burden varies greatly depending on the stage of pregnancy, socioeconomic status, and structural disparities (Mutahi et al. 2022; Pan et al. 2024).

It is estimated that 25.3% of pregnant women and 19.0% of women who had recently given birth in LMICs experience depression and other mental health disorders (Gelaye et al. 2016). These proportions are reported to be much higher than those typically observed in high-income countries (Pan et al. 2024). Estimates specific to the African region are not available, as no systematic review published to date has focused particularly on the region, but it could be assumed that the prevalence is higher than the LMIC average due to factors such as widespread poverty, exposure to sexual and gender-based violence and exposure to emergency and conflict situations. Such a situation is compounded by poor access to and unavailability of mental health services (Herrman et al. 2006; WHO 2019).

Addressing the mental health needs of women globally is critical in achieving maternal and child health goals as stipulated in the sustainable development goals (SDGs) (Pan et al. 2024; Gelaye et al. 2016). There is indeed strong evidence of the impact of maternal mental health on maternal and child mortality and morbidity. The negative impacts of poor mental health on women’s health include low utilization of antenatal and postnatal care services, poor adherence to prescribed health regimes, and risks of obstetric and preterm labor. Impacts on children’s health include low birth weight, malnutrition and stunting, delays in immunization, diarrheal and infectious diseases due to poor infant breastfeeding practices, and poor or lack of healthcare-seeking behaviors by mothers with depressive symptoms (Pan et al. 2024).

The SDG 3 target 3.4 to *'reduce by one-third premature mortality from non-communicable diseases through prevention and treatment and promote mental health and well-being'* aims to prevent and manage mental health problems (2015). The strategies to achieve this target include integrating mental health in maternal health policies, plans and activities in countries, and including mental health programs into ongoing maternal health services at the primary healthcare level (2015). These recommendations have generally hardly been embraced in LMICs and the African region. No mention is made, for instance, of the need to address perinatal mental health disorders in the African Union’s 2014 Status Report on Maternal, Newborn and Child Health (Grace and Sansom 2003). Moreover, very few African countries specifically mention issues related to maternal mental health in their health policies. Improving delivery of maternal mental health programs in LMICs necessitates acting on 4 misconceptions or myths, including 1) maternal depression is rare; 2) maternal depression is not relevant to maternal and child health programs; 3) only specialists can treat maternal depression; and 4) it is not possible to integrate mental healthcare into maternal and child health programs (2014).

The prevalence of common maternal mental health disorders is as high as 33% in selected LMICs, where there exists a wide intervention gap for the disorders (WHO 2019), in addition to limited awareness and insufficient mental health infrastructure at all levels. The African continent is poorly represented with respect to the existing body of evidence on these conditions (McNab et al. 2022). Emerging evidence points to the increasing disease burden associated with maternal mental illnesses, primarily in adolescent and young women in Africa mainly driven by underlying environmental and societal problems such as abject poverty, a lack of adequate and appropriate healthcare infrastructure (GBD 2019 Mental Disorders Collaborators 2022) and emerging and re-emerging public health problems (e.g., COVID-19 pandemic), necessitating integration of maternal mental health services into basic healthcare delivery system (Rahman et al. 2013).

Attention to maternal mental health is relevant to maternal and child health programs in the African region as the region continues to grapple with high rates of maternal and child mortality. The region still contributes to more than half of all maternal deaths in LMICs and reports the highest neonatal mortality rate in the world (WHO 2016; 2018). Some of the challenges in integrating mental healthcare into routine primary care in the region include inadequate mental health specialists; lack of prescribing guidelines; limited evidence on feasible detection and treatment strategies for maternal mental disorders; and stigmatizing attitudes toward mental illnesses among primary healthcare providers and the society in general (Baron et al. 2016). Anticipating demand for services by the healthcare system is also made difficult by the lack of evidence on the scale of the burden of maternal mental ill-health (Baron et al. 2016). Despite growing evidence on the effectiveness of community-based non-mental health specialist-led interventions (e.g., use of community health workers or promoters), such alternatives are also yet to be explored by many countries in the region (Rahman et al. 2013).

While there are a few reviews on maternal mental health in Africa, their scope is limited, either by focusing on specific populations (e.g., adolescent or young mothers) or by emphasizing specific mental health conditions (e.g., depression and anxiety) (Dadi et al. 2020; Endomba et al. 2021). There have been no systematic reviews on mental health disorders and their determinants in childbearing women in Africa accounting for various aspects such as short- and long-term outcomes, as well as availability and access to mental health services. Addressing this gap is crucial for a more comprehensive understanding of the mental health landscape and the development of large-scale research and effective interventions for this vulnerable population. There is thus a need to comprehensively and systematically document what is known about perinatal mental disorders in Africa.

The current review comprehensively assesses available evidence on the topic to inform the research and development ecosystem of maternal mental health in Africa. The review had five objectives:

1. to determine the prevalence of different types of mental health problems and/or disorders in perinatal women.
2. to document types of measurement/diagnosis methods/tools used.
3. to assess risk profiles (risk factors) for these problems and/or disorders.
4. to assess health-seeking behaviors, and type and quality of mental health services accessed by perinatal women.
5. to document the short- and long-term effects of maternal mental health (on pregnancy outcomes, maternal health, child health, and social/developmental issues).

Aligning with these objectives, this systematic review endeavors to rigorously evaluate and synthesize results for each objective, discern patterns and gaps in existing evidence, and facilitate explicit discussions on the broader contextual underpinnings of maternal mental health in Africa. It employs an explicit, transparent, peer-reviewed search strategy, synthesizes findings from individual studies, and provides a comprehensive summary along with relevant recommendations to guide future maternal mental health research and implementation initiatives in the continent.

### Methods and search strategy

**Fig. 1:**
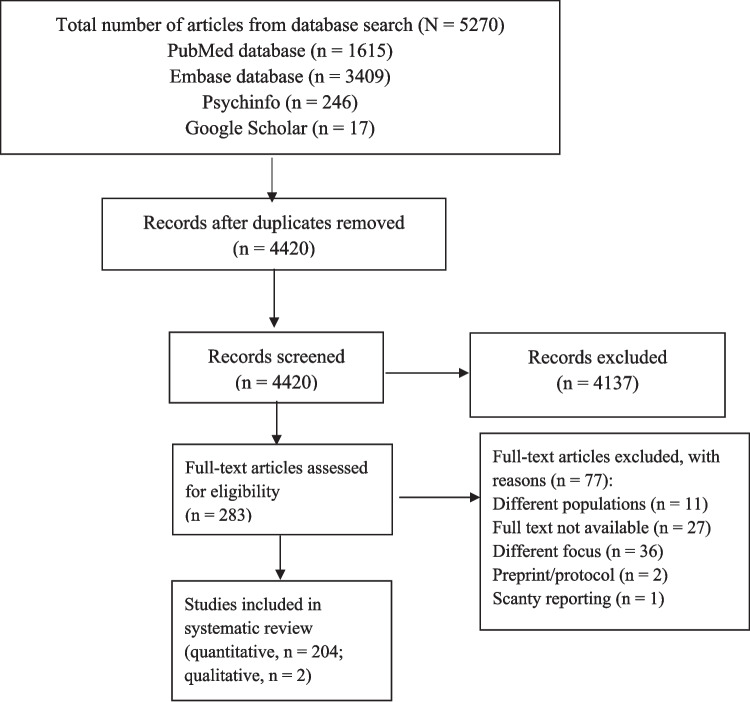
PRISMA flow diagram illustrating the article selection process

The Preferred Reporting Items for Systematic Reviews and Meta-Analyses (PRISMA) checklist (Page et al. 2021) was used to present the screening and eligibility of studies for the review and to summarize the findings, primarily for quantitative studies (Fig. 1). Eligible studies were: (1) quantitative studies that addressed at least one mental health disorder (2) for which potential confounders were controlled using robust statistical procedures (e.g., multivariable analyses); and (3) that obtained ethical clearances from wherein appropriate respective institutions. All included quantitative studies involved descriptive and analytic (inferential statistical) analyses; thus, descriptive findings were part of the synthesis. Descriptive and qualitative studies which addressed one or more perinatal mental health disorders and met third criteria were also included in the review. References of eligible studies were cross-checked to retrieve and include all relevant studies in the review. Both published and grey literature available in English on perinatal mental health disorders for the period January 2010 through to June 2024 was retrieved using electronic search engines.

A comprehensive search was conducted in PubMed/Medline, Embase and PsycInfo. The search was carried out using a combination of MeSH and variation terms as title/abstract headings. Google Scholar was also used for supplementary manual searches of eligible articles. Search/MeSH terms that are generally likely to relate to the main theme of the review were used. These included “pregnancy”, “postnatal”, “postpartum”, “prenatal”, “antenatal”, “perinatal”, “maternal”, “women”, “reproductive age women”, “mental health”, “anxiety”, “depressive”, “depression”, “somatic disorders”, “maternal dysthymia”, “psychosis”, “postpartum obsessive compulsive disorders”, “birth-related post-traumatic stress disorders”, “mental health", "mental health disorders” “maternal health services”, “Africa”, “sub-Saharan Africa”, “East Africa”, “West Africa”, “South Africa”, “Central and Southern Africa”, and “North Africa,” including names of specific African countries. However, specific search terms were established for specific databases and the full search strategy is attached (see Supplementary 1). The term ‘perinatal mental health’ has been used interchangeably with ‘maternal mental health’ to consistently present the findings in this article (Supplementary 1). The review was registered in PROSPERO (CRD42022369309).

After further screening for duplicates, titles and abstracts of all studies were screened for initial eligibility. The screened articles from the primary review were then moved to the phase of data extraction. Data extraction focused on overall study characteristics, study designs, participant characteristics, exposures/factors and outcome variables and their measurements. Variables of interest included maternal mental health during the perinatal period, its determinants, and short- and long-term health/behavioral effects. In addition, the quality of evidence in each study was critically appraised (Supplementary Tables 2 and 3).

### Quality appraisal of individual articles

The National Institute of Health (NIH) quality assessment tool was used to critically appraise the reliability, validity and overall quality of included articles (n.d). A standard assessment was done regarding: (i) the appropriateness of the research objective to the research question of the systematic review; (ii) the study population and eligibility of participation; (iii) the provision of sample size justification; (iv) study power and sampling; (v) variable measurements of exposure and outcome including a clear definition of dependent and outcome variables; (vi) report on loss to follow-up; (vii) blinding of participant or investigator; (viii) identification of covariates/confounders; and (xi) stratification and descriptive analyses. Articles were rated as good, fair, or poor. Each reviewer explained the quality rating of each article included in the review. Any misclassifications or rating disagreements were resolved within the research team.

The assessment of qualitative studies was done using the Critical Appraisal Skill Program (CASP) tool. The CASP checklist has ten items that look at the significance as well as clarity of research goals and appropriateness of the research design. This tool also assesses the methodology used in addressing the research question(s), recruitment strategies, data collection, data analysis, findings, ethical consideration and value of the research. Key findings from included quantitative and qualitative studies were synthesized narratively, providing a comprehensive overview of the key themes and patterns identified across studies, given the absence of a meta-analysis component due to heterogeneity. The extraction and synthesis of descriptive aspects such as specifics about study countries/regions, study characteristics (e.g., study design, participants, sampling strategy, and data sources), as well as measurement or diagnostic tools used for assessing and/or diagnosing maternal mental problems and/or disorders and their epidemiology, including prevalence, predominantly arose from the descriptive components of eligible and included studies. Furthermore, the identification of diverse determinants associated with these problems/disorders and synthesis of associated outcomes was primarily derived from the analytical components of the eligible and included studies. An independent consultant identified potential articles based on a priori* criteria* and extracted the data based on the listed variables. AA led the preparation and compilation of the systematic review. AA, FMW, EMS and CW contributed to the writing and critical review and revision of the manuscript. All authors approved the final manuscript for submission to the journal.

A comprehensive overview of eligible studies, encompassing details on study countries/regions, study characteristics, and measurement or diagnostic tools used to assess/diagnose maternal mental health problems and/or disorders is provided below.

### Eligibility of studies

Two hundred and six out of the 283 full-text articles that were evaluated for eligibility were included in the narrative synthesis (see Supplementary Figure). Based on the NIH quality assessment tool that was used to critically evaluate the articles from quantitative studies for reliability, validity, and overall quality, the quality of evidence in 145 articles was found to be of good quality, 54 of fair quality, and 5 of poor quality (Supplementary Table 2). The 2 qualitative studies included in the review received a fair rating according to CASP criteria (Supplementary Table 3).

Details on study countries, study designs, study participants, sampling strategy, data sources, and the stage of pregnancy (antenatal, postnatal) or non-pregnant status, by study type, as extracted from the eligible and included studies in Supplementary Table 1. In this Table, studies are systematically organized, starting from less robust study designs to more rigorous ones. In Table 1, data on the measurement and/or diagnostic tools of mental health problems and/or disorders, determinants of mental health, effect measures, short- and long-term effects of mental health (outcomes), source of healthcare assessed, and perception of healthcare quality, by types of mental health problems and/or disorders, are presented.

**Table 1:** Maternal mental health problems in Africa: assessment tools, consequences and healthcare utilization (perception and quality) by type of mental health problems/disorders

Author, year	Assessment/diagnosis tools of mental health status	Summary findings	Determinants of mental health and their effect measures	Short- and long-term effects of mental health (outcomes)	Source of healthcare assessed	Perception of healthcare quality
Depressive symptoms and depression						
Ngocho et al. 2022	Edinburgh Postnatal Depression Scale (EPDS)	Women who are not married, have limited support from their partners or have experienced violence are more vulnerable to depressive symptoms during pregnancy	Lifetime experience of violence, perceived availability of support and partner support	NR	Formal healthcare (Government facilities)	NR
Adeoye et al. 2022	EPDS (≥ 12 score)	Prevalence of antepartum depression was 14.1%. Factors associated with APD were high income (≥ 20,000) and perceived stress: moderate and high stress	Perceived stress and social support, intimate partner violence and binge eating	NR	Formal healthcare (4 Government facilities)	NR
Umuziga et al. 2022	EPDS (≥ 12)	Prevalence of antenatal depressive symptoms was 26.6%. Unwanted pregnancy, parity, exposure to stressful life events (violence), and partner support were linked to antenatal depressive symptoms	Exposure to stressful life events like violence and social support	NR	NR	NR
Gureje et al. 2022	EPDS (≥ 12)	Intervention group had lower levels of depressive symptoms than the control group and better parenting practices	NR	NR	Formal healthcare (30 primary maternal care clinics)	NR
Larsen et al. 2022	Patient Health Questionnaire—9 (PHQ-9)	14.6% ever had moderate to severe depression (MSD) during pregnancy or postpartum; 8.6% of women living with HIV had MSD in pregnancy and 9.0% in postpartum MSD. Stigma, food insecurity and unintended pregnancy were associated with higher risk of MSD	Social support, HIV related stigma, abuse and unintended pregnancy	NR	Formal healthcare facilities (6 Government facilities)	NR
Bitew et al. 2017	PHQ-9	28.7% of women had antenatal depressive symptoms (PHQ-9 score ≥ 5). Women with antenatal depressive symptoms had more than twice the odds of self-reported complications in pregnancy (OR = 2.44, 95% CI: 1.84, 3.23), labor (OR = 1.84 95% CI: 1.34, 2.53), and the postpartum period (OR = 1.70, 95% CI: 1.23, 2.35) compared to women without these symptoms	NR	Pregnancy complications: oedema, blurred vision, severe abdominal pain, abnormal vaginal discharge, burning sensation at urination, and severe headache; labor complications such as severe headache, convulsion, hemorrhage, unconsciousness, fever, premature rupture of membranes, prolonged labor, and retained placenta; postpartum complications (edema, blurred vision, severe abdominal pain, burning sensation at urination, severe headache, convulsion, hemorrhage, unconsciousness and fever. Neonatal complications: difficulty of breathing or fast breathing and convulsions or spasms	NR	NR
Bitew et al. 2017	PHQ-9	Antenatal depressive symptoms: 28.7%; nearly two-thirds, 783 women (62.6%), delivered in healthcare institutions. After adjusting for potential confounders, women with antenatal depressive symptoms had increased odds of reporting institutional birth [adjusted Odds Ratio (aOR) = 1.42, 95% Confidence Interval (CI): 1.06, 1.92] and increased odds of reporting having had an assisted delivery (aOR = 1.72, 95% CI: 1.10, 2.69) as compared to women without these symptoms. However, the increased odds of institutional delivery among women with antenatal depressive symptoms was associated with unplanned delivery care use mainly due to emergency reasons (aOR = 1.62, 95% CI: 1.09, 2.42) rather than planning to deliver in healthcare institutions	NR	Assisted delivery	Healthcare facility	NR
Khalifa et al. 2018	EPDS and 10-items Hopkins Symptoms Checklist (HSCL-10)	Prevalence of postnatal depression symptoms by EPDS was lower at eight months compared to three months after birth (3.6% at eight months (8/223) compared to 9.2% at three months (22/238), *p* < 0.001). Eight Mothers exhibited depression symptoms eight months after birth. Depressed mothers at three months had a 56% reduction in EPDS mean scores by eight months and 96.4% of participants either remained in the same EPDS category or improved eight months after birth. Four participants with major depression symptoms at eight months were also depressed three months after birth and four participants had new onset depression symptoms. The HSCL-10 measured higher distress than EPDS across the two screening points (19.3% at three months, 9.1% at eight months postpartum, *p* < 0.001)	Strong and positive correlations between depression and distress	NR	NR	NR
Wong et al. 2017	EPDS	Overall, 11% of women had EPDS scores suggesting probable depression, and 6% reported self-harming thoughts. Younger women reported more depressive symptoms. Report of self-harming thoughts was 11% in younger and 4% in older women (*p* = 0.003). In multivariable analysis, age remained significantly associated with depressive symptoms and report of self harming thoughts. Level of HIV-related stigma and report of intimate partner violence modified the association between age and depressive symptoms	Younger age associated with more depressive symptoms (β = 0.9, 95% CI: 0.1–1.8, *p* = 0.04) and self-harming thoughts (aOR = 3.2, 95% CI: 1.5–6.6, *p* = 0.002)	NR	NR	NR
Yotebieng et al. 2017	PHQ-9	51 (11.8%) had a PHQ-9 score ≥ 15 including 15 (3.5%) with a score ≥ 20. At six weeks postpartum, 67 (15.5%) were LFTU and 331 (76.4%) were in care and had accepted all available PTMCT services. prenatal depression assessed with a PHQ-9 score ≥ 15 was not a strong predictor of LTFU among newly diagnosed HIV-infected women in Kinshasa, DRC	NR	NR	NR	NR
Mossie et al. 2017	Local language version of Beck Depression Inventory (DDI) (> = 14)	Magnitude of depression was 31.1%. Pregnant women with low level of income (AOR = 3.66 (95%CI; 1.12, 11.96)), unmarried (AOR = 4.07 (95% CI; 1.18, 14.04)) and housewives (AOR = 4.24 (1.38, 13.03)) were risk groups for depression	Women who were unmarried (AOR = 4.07 ((95% CI; 1.18, 14.04); pregnant women with a low level of income (monthly income of < 1500 Birr) than women who earn a monthly income of more than 1500 Birr ((AOR = 4.06; 95% CI (1.32, 12.49)); housewives, compared to women who were involved in private jobs (AOR = 4.24 (1.38, 13.03))	NR	Public health facility	NR
Tuthill et al. 2017	PHQ-9	Most (80.9%) of the samples reported at least some symptoms of depression prenatally. Rates of depression were lower postpartum (47.1%). In multivariate models, higher prenatal depression scores significantly predicted a lower likelihood of EBF at six-weeks postpartum after adjusting for demographics, condition, and intentions (AOR = 0.68, p < 0.05)	NR	Exclusive breastfeeding: women who were more depressed prenatally were less likely to be EBF at 6 weeks (AOR = 0.68, 95% CI [0.49, 0.95], *p* = 0.04	NR	NR
Gebremichael et al. 2017	WHO Self-Reported Questionnaire (SRQ-20) with 20 items	Prevalence of perinatal depression among the study period was 26.7%. monthly income, number of pregnancies, age at marriage, health problems during pregnancy, alcohol use, husband smoking status, history of previous depression, and family history of psychiatric disorders were the independent factors associated with perinatal depression	Monthly income AOR (95% C.I): 4.2 (1.9, 9.3), parity [AOR (95% C.I): 0.14 (0.03, 0.65)], pregnancy complications AOR (95% C.I): 5 (2.5, 10.4), husband smoking status [AOR (95% C.I): 4.12 (1.6, 10.6)], history of previous depression AOR (95% C.I): 2.7 (1.54, 4.8), and family history of psychiatric disorders ([AOR (95% C.I): mental health 2.7 (1.54, 4.8), depression 3.6 (1.4, 9.1) were the independent factors associated with perinatal depression	NR	NR	NR
Rogathi et al. 2017	EPDS	Exposure to at least one type of IPV increased the odds of PPD more than three times (AOR = 3.10; 95% CI: 2.04–4.40) as compared to those women who were not exposed to IPV during their pregnancy. Among women exposed to emotional violence, women with no previous history of depression were also at higher risk of developing postpartum depression as compared to women who were having previous history of depression (AOR = 2.79; 95% CI: 1.76–4.42) and (AOR = 0.89; 95% CI: 0.38–2.08)	Depression during pregnancy: Exposure to IPC (during pregnancy, AOR 2.51; 95% CI = 1.36–3.76). Depression after pregnancy: physical and sexual violence (AOR 2.15; 95% CI = 1.13–4.11 and 1.98, 95% CI = 1.22, 3.23)	NA	NR	NR
Belay et al. 2018	BDI-II	Prevalence of antenatal depression was 17.9% [95% CI (14.0, 22.0%). Pregnancy planning [AOR: 0.04; 95% CI (0.014, 0.114), social support [AOR: 0.21; 95% CI (0.07, 0.66), and marital conflict [AOR: 6.45; 95% CI (2.1, 17.9)] were significantly associated with antenatal depression	Marital conflict [AOR = 6.45(2.1, 17.9)], planning current pregnancy [AOR = 0.04(0.01, 0.11)] medium social support [AOR = 0.21 (0.07, 0.66)]	NA	Hospital	NR
Pingo et al. 2017	EPDS	PPH was present in 49.1% of the participants at day 3 postpartum whilst PPD was present in 33.3% of participants on day 3 postpartum and in 45.6% at week 6. Participants meeting the clinical cut-off for both PPH and PPD on day 3 (17.5%) had significantly higher depression scores at week 6 than those with only PPH (*p* = 0.010) or only PPD (*p* = 0.035) on day 3. Depression scores on day 3 and lower social support scores at week 6 were predictive of PPD at week 6	PPD at week 6: social support	NA	Hospital	NR
Brittain et al. 2017	EPDS	11% and 19% had elevated EPDS scores using thresholds described in the original development of the scale (scores ≥ 13 and ≥ 10, respectively). Social support and stigma were highly interrelated and were associated with depressive symptoms. Stigma was observed to moderate the association between social support and depression scores; when levels of stigma were high	Maternal age, the report of an unintended pregnancy and higher levels of social rejection and internalized shame remained significantly associated with depression scores. A one-unit increase in social rejection was associated with a 0.7-unit increase (95% CI: 0.1, 1.3; *p* = 0.025) in depression score. A clear dose–response relationship was observed between tertiles of HIV-related stigma and increasing depression scores across all levels of social support	NA	NR	NR
Natamba et al. 2017	Center for Epidemiologic Studies-Depression scale (CES-D)	Association between FI and depressive symptoms severity was moderated by SS i.e. was stronger among women in the low SS category (adjusted beta (95%CI): 0.91 (0.55; 1.27)) than for women belonging to the high SS group (0.53 (0.28; 0.78)) (adjusted p-value for interaction = 0.026)	Food insecurity	NA	Hospital	NR
Barthel et al. 2017	PHQ-9	Three distinct classes of depressive symptoms were characterized by an asymptomatic trajectory (91.5%), by recurrent risk (4.3%) and by postnatal risk (4.3%). The longitudinal course of depressive symptoms was strongly associated with anxiety symptoms (χ2 = 258.54, *p* < 0.001; φ = 0.577). Among other factors, higher levels of anxiety, new pregnancy 2 years after birth, economic stress, and family stress were associated with the risk classes	Recurrent depressive symptoms: anxiety symptoms 1.13 (1.03, 1.25); Economic stress 1.93 (1.25, 2.96); mother pregnant since last birth 3.11 (1.10, 8.79). Postnatal depressive symptoms: anxiety symptoms 1.12 (1.01, 1.24); Family stress 1.98 (1.06, 3.68)	NA	Hospital	NR
Mbawa et al. 2018	EPDS	Prevalence of postpartum depression (PPD) among adolescent mothers (ADLM) (13.0%) than among adult mothers (ADM) (7.2%) (*p* < 0.001) in Mashonaland Central and Bulawayo provinces of Zimbabwe. The following were shown to significantly associate with PPD among ADLM: (*p* < 0.05): (i) abandonment by a partner, (ii) lack access to childhood needs, (iii) bad relationships within families, (iv) social insecurity, (v) prenatal depression (vi) unplanned pregnancies, (vii) lack of information about contraception, (viii) negative perception of teenage pregnancy, (ix) absence of both parents during childhood and negative familial relationships during childhood [OR > 1, 95% CI; *p* < 0.05)	Prenatal depression, unplanned pregnancy, lack of information about contraception before first pregnancy, negative societal response upon interception of news about pregnancy, not living with both parents during pregnancy	NA	NR	NR
Rodriguez et al. 2018	10-item EPDS	Half of the women (age = 29 ± 5) reported depression prenatally and one-third reported depression postnatally. In multivariable logistic regression, not living with their male partner, nondisclosure of HIV status, and postnatal depression predicted cognitive delay; decreased prenatal male involvement predicted delayed gross motor development (*p* < 0.05)	NA	Infant cognitive delay at 12 months; gross motor developmental delay at 12 months	NR	NR
Harrington et al. 2018	EPDS and PHQ-9	1 in 10 women screened positive for probable antenatal depression, whereas 1–6% screened positive postpartum. Sensitivity analyses to account for loss to follow-up suggested that postpartum depression prevalence could have ranged from 1–11%. At postpartum time points, 0–3% of participants screened positive for incident probable depression	NA	NR	Hospital	NR
Toru et al. 2018	PHQ-9	Magnitude of postpartum depression among the study population was 102 (22.4%, 95% CI: 19.84–24.96). Postpartum depression is higher among mothers with age range between 18 and 23 years, unplanned pregnancy, child having sleeping problems, domestic violence, unsatisfied marital relation, poor social support, history of previous depression and substance use	Age range between 18 and 23 years (aOR 3.89 95%CI: 1.53–9.90), unplanned pregnancy (aOR 3.35 95% CI: 1.701–6.58), a child having sleeping problems (aOR 3.72 95%CI: 1.79–7.72), domestic violence (aOR 2.86 95%CI 1.72–8.79), unsatisfied marital relation (aOR 2.72 95% CI 1.32–5.62), poor social support (aOR 4.30 95% CI 1.79–10.30), history of previous depression (aOR 7.38 95% CI 3.12–17.35) and substance use (aOR 5.16 95% CI 2.52–10.60)	NR	NR	NR
Anokye et al. 2018	PHQ-9	Postpartum depression was 7%. The severity ranged from minimal depression to severe depression. Psychosocial support proved to be the most effective intervention (*p* = 0.001) that has been used by healthcare workers to reduce depressive symptoms	Respondents who were employed were 4.7 times more likely to develop depressive symptoms: adjusted Odds Ratio (AOR) = 4.72 [95% CI 1.021–14.01]	NR	NR	NR
Ongeri et al. 2018	EPDS	18.7% (95% CI: 13.3–25.5) were found to have postpartum depression using an EPDS cut-off of 10. In multivariate analyses, the odds of having postpartum depression was increased more than seven-fold in the presence of conflict with a partner (OR = 7.52, 95% CI: 2.65–23.13)	Conflict with a partner (OR = 7.52, 95% CI: 2.65–23.13)	NR	NR	NR
Osok et al. 2018	EPDS and PHQ-9	32.9% (*n* = 58) tested positive for a depression diagnosis using PHQ-9 using a cut-off score of 15 + . However on multivariate linear regression, after various iterations, when individual predictors using standardized beta scores were examined, having experienced a stressful life event explained the most variance in the caregiver burden, followed by absence of social support for pregnant adolescents, being diagnosed with HIV/AIDS and being young	Having experienced a stressful life event (B = 3.27, *P* = 0.001, β = 0.25), absence of social support for pregnant adolescents (B = − 2.76, *p* = 0.008, β = − 0.19), being diagnosed with HIV/AIDS (B = 3.81, *p* = 0.004, β = 0.17) and being young (B = 2.46, *p* = 0.038, β = 0.14)	NR	NR	NR
Rotheram-Fuller et al. 2018	EPDS	Depressed mothers were significantly less educated, had lower incomes, and were less likely to be employed or to have electricity; were more likely to report problematic drinking of alcohol, experience food insecurity, interpersonal partner violence, and to be HIV seropositive. At 36 months, the pattern of maternal depressed mood over time was significantly associated with children’s compromised physical growth, both in weight and height, and more internalizing and externalizing symptoms of behavior problems	Depressed mothers were significantly less educated, had lower incomes, and were less likely to be employed or to have electricity; were more likely to report problematic drinking of alcohol, experience food insecurity, interpersonal partner violence, and to be HIV seropositive	Children’s compromised physical growth, both in weight and height, and more internalizing and externalizing symptoms of behavior problems	NR	NR
Shitu et al. 2019	EPDS (≥ 8)	Prevalence of postpartum depression was 23.7% with 95%CI: 20.3–27.2. The participant mothers who are divorced/widowed/unmarried, unwanted pregnancies, unpreferred infant sex, infant illness, and low social support was independent predictor of postpartum depression	Mothers who were divorced/widowed/unmarried (AOR = 3.45 95%CI: 1.35–8.82), unwanted pregnancy (AOR = 1.95 95%CI: 1.14–3.33), unpreferred infant sex (AOR = 1.79 95%CI: 1.13–2.86), infant illness (AOR = 2.08 95%CI: 1.30–3.34) and low social support (AOR = 3.16 95% CI: 1.55–6.43) was independent predictors of postpartum depression	NA	NR	NR
January and Chimbari 2018	DSM-5	Pooled prevalence of PND across the two districts was 26.0% (95% CI: 19.04–31.74). Having insufficient food in the household, intimate partner violence and having a child with a birth weight under 2500 g significantly increased the likelihood of PND	Having insufficient food in the household adjusted odds ratio (aOR) = 2.8 (95% CI: 1.2–6.1); intimate partner violence (aOR = 2.4 (95% CI: 1.1–5.6) and having a child with a birthweight under 2500 g aOR = 2.5 (95% CI: 1.2–5.3) significantly increased the likelihood of PND	NR	District hospital	NR
Chorwe-Sungani and Chipps 2018	EPDS	EPDS was 19% (95% CI 15.5% – 22.5%). The key risk factors that predicted antenatal depression were: ‘being distressed by anxiety or depression for more than 2 weeks during this pregnancy’; ‘feeling that a relationship with a partner is not an emotionally supportive one’; ‘having major stresses, changes or losses in the course of this pregnancy’; ‘feeling that father was critical of her when growing up’; and ‘having history of feeling miserable or depressed for ≥ 2 weeks before this pregnancy’	Being distressed by anxiety or depression for more than 2 weeks during this pregnancy (OR = 4.1 [2.1–7.9], p ≤ 0.001); feeling that a relationship with a partner is not an emotionally supportive one (OR = 3.5 [1.4–8.4], *p* = 0.01); having major stresses, changes, or losses in the course of this pregnancy (OR = 3.2 [1.7–6.2], *p* = 0.01); feeling that father was critical of her when growing up (OR = 3.2 [1.4–7.6], *p* = 0.01); and having a history of feeling miserable or depressed for ≥ 2 weeks before this pregnancy’ (OR = 2.4 [1.3–4.4], *p* = 0.01)	NR	NR	NR
Peltzer et al. 2018	EPDS	48.7% scored ≥ 13 on the EDPS while postnatally (at 12 months) the prevalence was 35.6%. Mothers who did not have depression before or after were 205 (50.1%), those who had depression before and after were 58 (14.4%), those who had depression only before were 81(20.1%), and those who had depression only after were 59 (14.6%). Less education and physical and psychological intimate partner violence were associated with sustained perinatal depression. Psychological partner violence, lack of male involvement during pregnancy and non-adherence to antiretroviral treatment were associated with depression	Perinatal depression: Less education and physical and psychological intimate partner violence. Depression: sustained psychological partner violence, lack of male involvement during pregnancy, and non-adherence to antiretroviral treatment	NR	NR	NR
Rwakarema et al. 2015	EPDS	33.8% had antenatal depression. Pregnancy related anxiety was associated with antenatal depression, pregnant women with a poor relationship with a partner and low/moderate socio-economic status	Pregnancy-related anxiety; (OR) 1.36, 95% confidence interval (CI) 1.23 to 1.5); Pregnant women with a poor relationship with partners and low/moderate socio-economic status	NR	NR	NR
Tomita et al. 2015	CES-D (> / = 10)	Depressive symptoms (CESD C 10) prior to pregnancy were associated with infant LBW. Women’s depressive symptoms prior to pregnancy are associated with the low birth weight of newborns and suggest that this association may not be limited to depression present during the ante-natal phase	NR	Low birth weight (AOR = 2.84, 95% CI 1.08–7.46)	Antenatal clinics	NR
Weobong et al. 2015	PHQ-9	PND was associated with an almost threefold increased risk of mortality up to 6 months (adjusted rate ratio (RR), 2.86 (1.58 to 5.19); *p* = 0.001). The RR up to 12 months was 1.88 (1.09 to 3.24; *p* = 0.023). PND was also associated with an increased risk of infant morbidity	NR	Mortality up to 6 months (adjusted rate ratio (RR), 2.86 (1.58 to 5.19); *p* = 0.001); mortality up to 12 months was 1.88 (1.09 to 3.24; *p* = 0.023); increased risk of infant morbidity	NR	NR
Bitew et al. 2016	PHQ-9	29.5% of participants had depressive symptoms. The majority (60.5%) of women had attended one or more ANC visits. Women with depressive symptoms had an increased risk of having more non-scheduled ANC visits, as well as an increased a number of emergency health care visits to both traditional providers and biomedical providers for pregnancy-related emergencies	NR	Increased risk of having more non-scheduled ANC visits (adjusted Risk Ratio (aRR) = 1.41, 95%CI: 1.20, 1.65), as well as an increased several emergency health care visits to both traditional providers (aRR = 1.64, 95% CI: 1.17, 2.31) and biomedical providers (aRR = 1.31, 95% CI: 1.04, 1.69) for pregnancy-related emergencies	Antenatal clinics	NR
Tefera et al. 2015	WHO SRQ-20	Prevalence of perinatal depression was about 31.5%. Maternal perceived difficulty of childcare, family History of mental illness, family visit during the perinatal period, history of child death, and husband smoking status were found as independent predictors of perinatal depression	Maternal perceived difficulty of childcare (AOR: 0.43; 95%CI = 0.20–0.93), family history of mental illness (AOR: 0.17; 95% CI 0.03–0.92), family visit during the perinatal period, history of child death and husband not using tobacco (AOR: 0.28; 95% CI = 0.12–0.67)	NR	NR	NR
Mohammed et al. 2014	EPDS	49.5% of mothers had PPD (29.5% had minor PPD and 20% had major PPD). Using logistic regression analysis, total household income, child sleeping hours, complications after delivery, and support of husband after delivery were found to be statistically associated with PPD	Low household income, child sleeping sufficient house, complications after delivery, no support of husband after birth	NR	NR	NR
Stellenberg et al. 2015	EPDS and BDI	50.3% of the mothers suffered from PND. A BDI analysis showed that of the participants who had PND, 28.8% was severe, 48.8% moderate and 22.5% mild. Factors influencing the development of PND included most participants (63.5%) were unmarried, 61.3% were unemployed and the majority (53.8%) had a history of a psychiatric illness. Significant associations between PND and unplanned and unwelcome babies (*p* < 0.01); partner relationships (*p* < 0.01) were identified	Factors influencing development of PND included unplanned and unwelcome babies (*p* < 0.01); partner relationships (*p* < 0.01)	NR	NR	NR
Sulyman et al. 2016	EPDS	Postnatal depression was 22.4%. Factors associated with the development of postnatal depression were unemployment, lack of support from the husband, and primiparity; others are unplanned pregnancy and physical illness in the mother	Unemployment [odds ratio (OR) = 0.49, 95% (CI) = 0.27–0.86), lack of support from the husband (OR = 0.34, 95% CI = 0.19–0.60), and primiparity (OR = 0.56, 95% CI = 0.35–0.88); unplanned pregnancy (OR = 0.56, 95% CI = 0.35–0.88) and physical illness in the mother (OR = 1.81, 95% CI = 1.77–2.79)	NR	Tertiary hospital	NR
Peltzer et al. 2016	EPDS	48.7% [95% CI: 44.8, 52.6] of women reported depressed mood. In multivariate analysis, not being employed, unplanned pregnancy, not having an HIV-positive child, poor antiretroviral therapy adherence, non-condom use at last sex, and intimate partner violence were associated with depressive symptoms	Not being employed (0.57 (0.37–0.88)), unplanned pregnancy (1.69 (1.22–2.33)), not having an HIV-positive child (0.28 (0.11–0.71)), poor antiretroviral therapy adherence (0.51 (0.36–0.72)), non-condom use at last sex (1.47 (1.05–2.06)), and intimate partner violence (1.99 (1.30–3.03))	NR	Community health centers	NR
Abate et al. 2021	PHQ-9	The prevalence of depression among HIV-positive pregnant women was 28.7%. Age (≥ 30 years), urban residency, having first pregnancy < 18 years, known HIV serostatus during pregnancy, and COVID-19-related knowledge were significantly associated with depression	Socio-demographic data, obstetric-related information, HIV and psychosocial support information, and COVID-19-related knowledge	NR	Formal healthcare facilities (Government facilities)	NR
Abebe et al. 2022	PHQ-9 and Center for Adherence Support Evaluation (CASE) Adherence Index	Prevalence of depression was 47.6%. Low income and non-adherence to ART were associated with depression among HIV-positive pregnant women	Adherence to ART, CD4 counts, and WHO HIV clinical Stage	NR	Formal health facility	NR
Adebowale et al. 2020	WHO SRQ-20	Prevalence of psychiatric morbidity was 11.6%	Socio-demographic characteristics and intimate partner violence	NR	Formal healthcare facility	NR
Acheampong et al. 2021	PHQ-9	Prevalence of moderate-severe depression was 11.9%. Depressed women had an increased risk of pre-eclampsia, cesarean section and episiotomy	Depression	Pre-eclampsia, cesarean section, and episiotomy	Formal healthcare facility	NR
Ahmed et al. 2021	EPDS	Prevalence of depression was 33.5%. SES, history of depression, history of depression, history of stressful conditions, familial support, unwanted pregnancy, and male preference were associated with depression	Previous psychiatry illness, family history of psychiatric illness, history of postpartum depression, stressful life event, lack of social support, gender and weight of baby	NR	Formal healthcare facility	NR
Adeyemo et al. 2020	EPDS	Having postpartum blues, not getting help with baby care, experienced intimate partner violence and having an unsupportive partner were identified as predictors of postpartum depression	Sociodemographic and maternal factors	NR	Formal healthcare facility	NR
Abebe et al. 2019	EPDS	Postpartum depressive symptoms among mothers were found to be 22.1. Stressful life events, domestic decision making, unplanned pregnancy, partner violence (and hospitalization of their babies were factors associated with depressive symptoms	Social support, intimate partner violence, women autonomy, stressful life event, history of mental illness and depression, and chronic illness	NR	Formal healthcare facility	NR
Abadiga 2019	EPDS	20.9% women had developed postnatal depression. Unplanned pregnancy, being a mother for the first time, history of previous depression, Domestic violence, history of substance use and poor social support were linked to postnatal depression	Social support, domestic violence, unemployment, substance use, stressful life event, presence of medical illness, and history of depression	NR	NA/community based study	NR
Adamu et al. 2018	EPDS	Prevalence of depression was 23.3%. Domestic violence was associated with postpartum depression	Domestic violence	Marriage dissatisfaction	Formal healthcare facility	NR
Desalegn et al. 2021	EPDS	Prevalence of antenatal depression was 36.4%. Being unmarried, experiencing IPV during pregnancy, ART non-adherence and experiencing internalized AIDS stigma were associated with antenatal depression	Social support, ART adherence, internalized AIDS stigma, and intimate partner violence	NR	Formal healthcare facility	NR
Tesfaye et al. 2021	PHQ-9	Prevalence of antenatal depression was 16.6%. Marital status, family history of depression, planning for pregnancy, history of abortion, and IPV were linked to antenatal depression	Social support status, Obstetric and clinical, intimate partner violence and family history of depression	NR	Formal healthcare facility	NR
Mostafa et al. 2021	HRSD	Prevalence of depression was 68.7%. About one-fifth were readmitted to NICU, 4% were LBW, and 3% were neonatal mortality	Economic status and obstetric characteristics	LBW, premature births, NICU admission, and neonatal mortality in 1st month	Formal healthcare facility	NR
Okafor et al. 2021	EPDS	Emotional and sexual violence were associated significantly with depression symptoms	Sociodemographic, substance use and trauma	NR	Formal healthcare facility	NR
Beyene et al. 2021	EPDS	Prevalence of antenatal depression was 24.45%. Being single, fear of pregnancy complication, history of chronic illness, unplanned pregnancy, history of stillbirth, negative life events and IPV were associated with antenatal depression	Social support, life-threatening events, intimate partner violence, and fast alcohol test	NR	NA/community-based study	NR
Dadi et al. 2021	EPDS	The cumulative incidence of diarrhea, ARI and malnutrition was 17.0%, 21.6%, and 14.4%, respectively	Social support	Infant ARI and malnutrition	NA/community-based study	NR
Zotova et al. 2022	PHQ-9	Disclosure of HIV status to partner was associated with lower depressive symptoms among women diagnosed prior to current pregnancy	Partner's HIV status, intimate partner violence and CD4 count	NR	Formal healthcare facility	NR
Beketie et al. 2021	EPDS	Magnitude of antenatal depression was 35.4%. Variables associated with antenatal depression were anxiety, unplanned pregnancy, and Primigravida being an uneducated mother and low educational level	Social support, intimate partner violence, threatening life events and partner's support	NR	Formal healthcare facility	NR
Wake et al. 2022	EPDS	19.7% of the postpartum women had depression. Occupational status, marital status, income management, sex of baby, history of child death, unplanned pregnancy, negative life event, substance use during pregnancy, history of depression, and marriage satisfaction were determinant of postpartum depression	Social support, domestic violence, negative life events, marriage satisfaction, partner support and relative present at last birthplace	NR	Formal healthcare facility	NR
Borie et al. 2022	PHQ-9	Depression among pregnant mother was found to be 27.2%. Women’s level of education, being elementary school, completing high school and above were associated with maternal depression	History of depression, substance use, husband support and medical illness	NR	Formal healthcare facility	NR
Rencken et al. 2022	EPDS	Prevalence of depression among mothers at initial contact was 72.0% and remain at 68.8% after follow-up. Employment and delivery method were associated with depression. Thoughts of self-harm were reported by 44.7% of mothers at baseline, and by 53.1% at follow-up	Thought of self-harm and anxious	NR	Formal healthcare facility	NR
Madeghe et al. 2021	EPDS	Prevalence of antenatal depression was 33.6%. Poor nutrition was associated with maternal depression	Poor nutrition	NR	Formal healthcare facility	NR
Okunola et al. 2021	EPDS	Prevalence of antepartum and postpartum depression were 6.3% and 8.8%, respectively. Antepartum depression, puerperal sepsis, domestic violence, age group, and household income were associated with postpartum depression	NR	NR	Formal healthcare facility	NR
Ayele et al. 2021	EPDS	Prevalence of depression among pregnant mothers who had IPV was 35%: physical abuse, more than one abuse, poor social support, and pregnant mothers whose partner drank for the past twelve months were associated with antenatal depression	Intimate partner violence, social support and presence of medical illness	NR	Formal healthcare facility	NR
Jihed et al. 2022	CES-D	Family income and work posture were significantly associated with MDD	Diagnosed with chronic illness, COVID-19 and a history of depression	NR	Formal healthcare	NR
Oladeji et al. 2022	EPDS	Being single and food insecurity were associated with being depressed	Physical, psychological and social quality of life	NR	Formal healthcare facility	NR
Keliyo et al. 2021	EPDS	24.3% of women had antenatal depression. Marital status, educational status, chronic medical illness, previous history of depression, and social support were associated with antenatal depression	Substance use, history of depression, social support, chronic medical illness, poor marital relationship and unwanted pregnancy	NR	Formal healthcare facility	NR
Sewnet et al. 2020	DASS-21	Prevalence of antenatal depression was 34.1%. Divorced, marital status, husband’s educational status was associated with depression among pregnant women during the COVID-19 pandemic	NR	NR	Formal healthcare facility	NR
Atuhaire et al. 2021	DSM-5	Prevalence of postpartum depression was 27.1%. Five factors associated with PPD were low perceived social support, HIV positive status, rural residence, obstetrical complications and the baby crying excessively	History of chronic illness, mode of delivery and complications in pregnancy	NR	Formal healthcare facility	NR
Bhushan et al. 2022	EPDS and PHQ-9	15% reported antenatal depression. Odds of probable perinatal depression decreased over time but were higher among those newly diagnosed) and on second line therapy as compared to previous defaulters. Odds of probable postpartum depression were lower for participants with high social support	NR	NR	Formal healthcare facility	NR
Mokwena and Mbatha 2021	EPDS	Prevalence of postnatal depression was 42.5%. Postnatal depression was associated with the participant’s partner having other sexual partners, adverse life events, low monthly income, and being financially dependent on others	NR	NR	Formal healthcare facility	NR
Anbesaw et al. 2021	EPDS	Prevalence of suicide ideation among women was 13.3%. single, history of abortion, depression, anxiety, poor sleep quality, stress, and intimate partner violence were predictors of suicidal ideation	Anxiety, history of abortion, history of mental illness, family history of mental illness and suicide attempts, chronic medical illness, and sleep quality	NR	Formal healthcare facility	NR
Beyene et al. 2021	EPDS	Cumulative incidence of LBW was found to be 27.76%. Antenatal depression in the second and third trimester of pregnancy were associated with LBW	Unplanned pregnancy, history of abortion, history of stillbirth, fear of pregnancy complications	LBW	NA/community-based study	NR
Kim et al. 2021	PHQ-9	Antenatal depression had very small or no association with maternal health service utilization. The study found that women with moderate or severe antenatal depression, those with perceived fair or poor health used fewer ANC visits and were less likely to use PNC within 7 days than those with perceived excellent, very good or good health	Intimate partner violence and partner support	Low ANC and PNC attendance	NA/community-based study	NR
Mwita et al. 2021	EPDS	Prevalence of depression was 15%. Polygamy and partner violence were risk factor for depression	Intimate partner violence, partner support, family support and HIV status	NR	Formal healthcare facility	NR
Oboro et al. 2022	EPDS	Prevalence of antenatal depressive symptoms in early pregnancy was 29.4%. Factors associated with antenatal depression were intimate partner violence, marital dissatisfaction, poor social support, past history of depression, previous pregnancy complication, low socio-economic status and unplanned pregnancy	History of miscarriage and preterm delivery, previous pregnancy complications, history of depression, substance use, intimate partner violence and social support	NR	Formal healthcare facility	NR
Jones et al. 2021	CES-D	Prevalence of depression among the cohort was 6.3%	Viral load counts, household food insecurity and history of depression	NR	Formal healthcare facility	NR
Ghoneim et al. 2021	EPDS	Emotional and sexual violence had significant roles as risk factors for depression during pregnancy	Intimate partner violence	NR	Formal healthcare	NR
Kaiyo-Utete et al. 2020	DSM-IV	Prevalence of antenatal depression was 23.47%. Intimate partner violence (IPV) and experiencing negative life events were risk factors for antenatal depression	Intimate partner violence, HIV status, negative life events, history of chronic conditions, and presence of chronic disease	NR	Formal healthcare facility	NR
Gordon et al. 2021	EPDS	Prevalence of antenatal depression was 13.0% and recurrent depression prevalence was 20.3%. Children of mothers who experienced depressed mood weighed less, were more aggressive, and hospitalized more often than children of never depressed mothers but were similar in cognitive and social development	Partner's HIV status, and intimate partner violence	NR	NA/community-based study	NR
Anato et al. 2020	EPDS (≥ 13)	Maternal depression was prevalent (22.8%) and was significantly associated with inappropriate complementary feeding and stunting (*P* < 0.05)	NR	NR	Formal healthcare facility	NR
Gebregziabher et al. 2020	DSM-5	Prevalence of postpartum depression was 7.4%. mothers with perceived low economic status, lack of partner support, unplanned pregnancy, and maternal illness after delivery had higher odds of postpartum depression	Partner support, history of mental illness, history of miscarriage, complications during perinatal period	NR	Formal healthcare facility	NR
Tiki et al. 2020	PHQ-9	Prevalence of depression during pregnancy was 32.3%. Low income, unplanned pregnancy and having history of abortion were associated with higher odds of depression	History of depression, chronic illness, and history of complications during pregnancy	NR	NA/community-based study	NR
Phukuta et al. 2020	EPDS	Prevalence of depression was 38.8%. Low educational level, contraceptives use prior to pregnancy and thoughts of self-harm were associated with postnatal depression	History of miscarriage, complications during pregnancy, and having a sick child	NR	NA/community-based study	NR
Belete et al. 2020	EPDS	Prevalence of postpartum depression was 33.8% for all mothers and 55.3% for with childhood history of sexual abuse. Age less than 25, family size (more than 5), history of childhood sexual abuse, joblessness and growing up with biological mothers were determinants of postpartum depression	Social support, history of childhood sexual abuse, abortion and depression, ability to care for child and substance use	NR	Formal healthcare facility	NR
Mokwena et al. 2020	EPDS	Prevalence of postnatal depression was 57.14%. Factors accounting for the depression were lack of support, not having preferred sex baby, low household income and having an older baby	Preferred baby's sex, planned baby and income	NR	Formal healthcare facility	NR
LeMasters et al. 2020	EPDS	Women experienced a double burden of physical and mental illness, expressed as pain in one’s heart. Receiving an HIV diagnosis unexpectedly during antenatal care was a key contributor to developing PND. This development was influenced by stigmatization and social support	NR	NR	Formal healthcare facility	NR
Govender et al. 2020	EPDS	Prevalence of prenatal depression was 15.9% and postpartum depression was 8.8%. Prenatal depression was associated with physical violence and verbal abuse. Postnatal depression was associated with physical violence, verbal abuse and intimate partner violence	Sexual and verbal abuse, IPV, partner support, history of abortion and repeated pregnancy	NR	Formal healthcare facility	NR
Dadi et al. 2020	EPDS	Prevalence of depression was 6.9%. Of these, 4.7% and 8.1% were in their second and third trimesters, respectively. Unplanned pregnancy, history of common mental health disorder and fear of giving birth to the current pregnancy were associated with depression	Social support, partner support, stress coping ability, and substance use	NR	NA/community-based study	NR
Bitew et al. 2019	PHQ-9	Incidence of postnatal depression was 38.8%. Higher intimate partner violence scores in pregnancy were significantly associated with greater risk of depressive symptoms	Number of chronic illness, alcohol use, history of adverse perinatal outcomes and pregnancy complications	NR	NA/community-based study	NR
Shitu et al. 2021	EPDS	Magnitude of antepartum depression was 27.6%. Unplanned pregnancy, complications during labor and delivery, previous history of child hospitalization, and satisfaction in marriage were associated with antepartum depression	Psychosocial support, intimate partner violence, substance use, and complications during pregnancy	NR	Formal healthcare facility	NR
Mbatha et al. 2020	EPDS	Prevalence of postnatal depression was 42.5%. No variable was significant after adjusting for other co-variates	Planned pregnancy, mode of delivery, history of baby's hospitalization, baby's HIV status, HIV status of mother and partner	NR	Formal healthcare facility	NR
Oladeji et al. 2019	EPDS	Prevalence of depression was 6.9% for adults mothers and 17.7% for adolescents. Adolescent mothers had poorer adjustment and attitudes to pregnancy, lower gestational age at birth, and low birth weight. Additionally, adolescent mothers had poorer maternal attitudes and parenting skills, and their infants had higher rate of undernutrition	NR	LBW and poor maternal attitude and parenting skills	Formal healthcare facility	NR
Lodebo et al. 2020	EPDS	Prevalence of antenatal depression was 23.3%. Factors associated with antenatal depression were being single, widowed or divorced), history of previous depression, family history of mental illness, intimate partner violence, unsatisfactory marital relation, lack of adequate social support and unplanned current pregnancy	Partner support, marriage satisfaction, and complications in pregnancy	NR	NA/community-based study	NR
Arach et al. 2020	EPDS	Prevalence of depression was 21.1%. Depression among the 77 women who had experienced perinatal death was 62.3% compared to 19.2%, among 1,712 live infants. Women who experienced a perinatal death were three times as likely to have postpartum depressive symptoms as those who had a live birth [adjusted prevalence ratio 3.45 (95% CI: 2.67, 4.48)	NR	NR	NA/community-based study	NR
Pellowski et al. 2019	Locally validated EPDS, with a score of ≥ 7	Five distinct trajectory patterns of depressive symptoms were identified: moderate levels of depressive symptoms during pregnancy but minimal postpartum (3.5%), minimal levels during pregnancy and in- creasing postpartum (3.7%), unstable levels peaking at 12 months postpartum (6.6%), mild levels with slight decrease postpartum (82.9%), and severe levels during pregnancy and postpartum (3.1%). Membership in the chronic severe symptom group was associated with stressful life events, sexual intimate partner violence and tobacco use	Unplanned pregnancy, HIV status, intimate partner violence and stressful life events	NR	Formal healthcare facility	NR
Modjadji et al. 2020	EPDS	Prevalence of depression was 22%. Odds of developing PND were 3.17 times more likely in women with babies aged six weeks and above (AOR = 3.17, CI; 1.39–7.23) and 4.50 times more likely in women living in households with an income of less than $115.55 (AOR = 4.50, CI; 1.03–19.74). Partner violence (AOR = 6.89, CI; 1.49–31.93) and stressful life events (AOR = 3.73, CI: 1.52–9.17) were associated with postnatal depression. Having partner support (AOR = 0.10, CI: 0.03–0.37) and receiving social support (AOR = 0.28, CI: 0.09–0.93) reduced the risk of developing postnatal depression	History of intimate partner violence, social support, and life stress	NR	Formal healthcare facility	NR
Mnisi et al. 2019	EPDS	Mothers’ accommodation during their babies’ admission to the neonatal unit (*p* = 0.002), having poor interpersonal relationships (*p* < 0.0001), and intimate partner violence (IPV) (*p* = 0.004) were significantly associated with screening positive for PND	Planned pregnancy, mode of delivery, HIV status of mother and medical conditions	NR	Formal healthcare facility	NR
Mbarak et al. 2019	EPDS	PPD was 20.5% in women who had pre-eclampsia or eclampsia. Factors associated with PPD included young age (AOR = 10.13 95% CI 1.99–52.02), being single mother (AOR = 3.18 95% CI 1.02–9.95), having lower educational level (AOR = 3.83 95% CI 1.45–10.16), having a perinatal death (AOR = 5.14 95% CI 2.53–10.45), lack of family support (AOR = 7.06 95% CI 1.25–39.90), and experience of stressful event during pregnancy (AOR = 15.14 95% CI 2.38–96.19)	Partner support, family support, partner relationship, financial problems and stressful event	NR	Formal healthcare facility	NR
Goweda et al. 2020	Arabic version of EPDS, with > 12 as cut-off for depression	Postpartum depression prevalence was 26.6% and suicidal ideation accounted for 4.6%. Factors significantly associated with high EPDS scores were a bad relationship with husband, having > 2 children, an unplanned pregnancy and an unhealthy newborn; with P-values of 0.000, 0.004, 0.000, and 0.018, respectively	Number of children, marital relationship, obstetric and newborn complications, substance use, gender of newborn, labor complications and mode of delivery	Suicide ideation	Formal healthcare facility	NR
Saeed et al. 2019	EPDS	Prevalence of depression was 16.8% (95%CI: 12.1–22.0%). Being currently unmarried (*p* < 0.001) and using lighting sources other than electricity (*p* = 0.004) were associated with higher risk of depression while being employed in other occupations (*p* = 0.001), and not cooking with firewood (*p* = 0.008) were associated with lower risk of depression	Type of cooking fuel, source of light in household, wealth index, and number of children	NR	Formal healthcare facility	NR
Dlamini et al. 2019	EPDS	47.4% women screened positive for postpartum depression. Unemployed ([OR] = 3.20, 95% CI: 1.17–8.79) and with poor social support from their partners (OR = 9.41, 95% CI: 3.52–25.14) were more likely to be depressed, while those who had less than 4 visits had lower odds of depression (OR = 0.32, 95% CI 0.11–0.92)	Planned pregnancy, maternal HIV status, partner support, condition of baby, and history of mental illness	NR	Formal healthcare facility	NR
Belete et al. 2019	Amharic version of BDI-II	Prevalence of antenatal depression was 15.20%. Urban residence [AOR = 6.8; 95% CI (1.97, 23.32)], being unmarried [AOR = 5.1; 95% CI (1.79, 14.63)], being government employee [AOR = 8.8; 95% CI (2.06, 37.12)] and merchant [AOR = 3.7; 95% CI (1.27, 10.91)], prim gravid [AOR = 5.3; 95% CI (2.03, 13.82)], not ANC following up on ANC [AOR = 8.7; 95% CI (3.46, 21.79)], intimate partner violence [AOR = 4.5; 95% CI (1.28, 15.52)], unplanned pregnancy [AOR = 6.2; 95% CI (2.37, 16.06)], and substance use [AOR = 5.6; 95% CI (2.12, 14.92)] were factors of depression	Substance use, intimate partner violence, and pregnancy planning	NR	NA/community-based study	NR
Alenko et al. 2020	BDI-II	Married mothers were 67% (AOR = 0.33, 95%CI: 0.15–0.75), housewives were 97% (AOR = 0.03, 95%CI: 0.01–0.14), private workers were 87% (AOR = 0.13, 95%CI: 0.04–0.44), and government employees were 84% (AOR = 0.16, 95%CI: 0.05–0.46), less likely to develop antenatal depression. Multigravida were 88% (AOR = 0.12, 95%CI: 0.04–0.37) less likely to develop antenatal depression. Third trimester pregnancy was four times (AOR = 4.04, 95%CI: 1.51–10.81) more likely to have depression. Mothers who had wanted pregnancy were 83% (AOR = 0.17, 95%CI: 0.04–0.81) less likely to develop antenatal depression compared with mothers having an unwanted pregnancy	Substance use, social support, and intimate partner violence	NR	Formal healthcare facility	NR
Necho et al. 2020	EPDS	More than one-fourth (27%) (95% CI 22.5, 31.5) have postpartum depression. Being single (AOR = 4.9, 95% CI 1.27, 16.74), dissatisfaction with child gender (AOR = 3.1, 95% CI 1.62, 6.69), unplanned pregnancy (AOR = 2.5, 95% CI 1.76, 7.23) and depression during current pregnancy (AOR = 3.2, 95% CI 2.81, 8.91) intimate partner violence; psychological (AOR = 6.5, 95% CI 1.98, 15.85), sexual and physical violence (AOR = 3.46, 95%CI 2.34, 18.55), current husbands alcoholism (AOR = 2.2, 95% CI 1.48, 5.34) from husband/partner-related variables and current substance use (AOR = 1.8, 95% CI 1.16, 3.75) were associated with postpartum depression	Social support, mode of delivery, desired gender of newborn, baby disease/defect, and depression during pregnancy	NR	Formal healthcare facility	NR
Davies al. 2016	EPDS	Stressors included poverty, unemployment, lack of support from partners, abuse, and death of loved ones, and were exacerbated by unwanted or unplanned pregnancies and the discovery of HIV positive status at antenatal appointments	Social support, substance abuse, HIV-positive status, and unplanned pregnancies	NR	Formal healthcare facility	NR
Heyningen et al. 2016	MINI Plus	Prevalence of Major depressive episode (MDE) was 22%, with 50% of depressed women also expressing suicidality. MDE was associated with multiple socioeconomic and psychosocial risk factors, including a history of depression or anxiety, food insecurity, experience of threatening life events and perceived support from family	Perceived social support, intimate partner violence, household food insecurity, and life-threatening experiences	Suicide ideation	Formal healthcare facility	NR
Manikkam et al. 2012	EPDS	38.5% had depression, and 38.3% thought of self-harm. HIV seropositive, prior history of depression, recent thoughts of self-harm, single marital status and unplanned pregnancy	Planned pregnancy, deliberate self-harm and HIV status	Thought of self-harm	Formal healthcare facility	NR
Wemakor and Mensah 2016	EPDS	Prevalence rates of child stunting and maternal depression were estimated at 16.1 and 27.8% respectively in Northern Ghana. Mothers with depression when compared with those without depression tended to be younger, be currently unmarried, belong to the poorest household wealth tertile, and were more likely to have low birth weight babies, so these characteristics were adjusted for. In an adjusted multivariate logistic regression model, children of depressed mothers were almost three times more likely to be stunted compared to children of non-depressed mothers (Adjusted OR = 2.48, 95% CI 1.29–4.77, *p* = 0.0011)	Maternal age, marital status and wealth index	Stunted growth of children	Formal healthcare facility	NR
Weobong et al. 2014	PHQ-9	Prevalence of antenatal depression was 9.9% (95%CI: 9.4%–10.2%). Depression was associated with: prolonged labor (RR 1.25, 95% CI 1.02–1.53); peripartum complications (RR 1.11, 95% CI 1.07–1.15);postpartum complications (RR 1.27, 96% CI 1.21–1.34); non-vaginal delivery (RR 1.19, 95% CI 1.02–1.40); newborn illness (RR 1.52, 95% CI 1.16–1.99); and bed net use during pregnancy (RR 0.93, 95% CI 0.89–0.98), but not neonatal deaths, still births, low birth weight, immediate breast feeding initiation, or exclusive breastfeeding. AND was marginally associated with preterm births (RR 1.32, 95% CI 0.98–1.76)	Peripartum complications, prolonged labor, and low birth weight	Preterm delivery	NA/community-based study	NR
Kaaya et al. 2016	HSCL-25	Postnatal depression was negatively associated [relative risk (RR = 0.80, *P* = 0.04], cumulative depression demonstrated a positive association with infant wasting (RR = 1.08, *P* < 0.01) and underweight (RR = 1.03, *P* < 0.01) after controlling for confounding factors. Variation in the association between depression and infant nutritional status was observed for episodic vs. chronic depression	Social support, weight-for-age, height-for-age or weight-for-height, anemia and HIV status	Infant malnutrition	Formal healthcare facility	NR
Gold et al. 2014	PHQ-9	32.7%) had PHQ-9 scores of 5–9, indicating mild depression; 42 (27.4%) had PHQ-9 scores of 10–14, indicating moderate depression; and 15 (9.8%) had scores of 15 or higher, indicative of moderate/severe depression. History of interpersonal violence with current partner predicted depression	Social support, interpersonal violence, history of depression, days child has been hospitalized, history of child loss and stillbirth	NR	Formal healthcare facility	NR
Mochache et al. 2018	EPDS	38.4%) found to have depressive symptoms and 27(10.7%) delivered preterm. The risk of delivering preterm was 3.8 times higher among those with depressive symptoms	Previous preterm history, social support, IPV, substance use and stressful life events	Preterm delivery	Formal healthcare facility	NR
Kaida et al. 2014	Modified HSC-15	Median CD4 count was 160 cells per cubic millimeter (interquartile range: 95–245), and mean depression score was 1.75 (s = 0.58) (39% with probable depression). Over 4.1 median years of follow-up, 104 women experienced 151 pregnancies. Mean depression scores did not differ across the time periods (*p* = 0.75). Increasing time on ART, viral suppression, better physical health, and “never married” were independently associated with lower mean depression scores. Findings were consistent when assessing probable depression	CD4 count at enrolment, BMI, and partner HIV status	NR	Formal healthcare facility	NR
Nydoo et al. 2017	EPDS	About 9.8% of women suffered from significant depressive symptoms, irrespective of HIV status. Prevalence rates of antenatal depressive symptoms did not differ significantly between HIV-infected and HIV-uninfected cohorts (*p* = 0.79). A new diagnosis of HIV infection (*p* < 0.0001) and maternal age (*p* = 0.03) were risk factors for antenatal depression. Unemployment was a borderline risk factor (*p* = 0.09) for the development of antenatal depression	CD4 cell count, unplanned pregnancy, substance use, relationship status and HIV status	NR	Formal healthcare facility	NR
Khalifa et al. 2016	EPDS	History of violence increased the odds of PND sevenfold, OR = 7.4 (95% CI 1.9 to 27.6). Older age of mothers decreased the odds of PND by almost 20%, OR = 0.82 (95% CI 0.73 to 0.92). Exclusive breast feeding and regular prenatal vitamins during pregnancy are associated with an 80% decrease in odds of PND, OR = 0.2 (95% CI 0.06 to 0.70) and 0.17 (95% CI 0.06 to 0.5), respectively	Polygamy, complications during pregnancy, presence of chronic illnesses, and sex of newborn	NR	Formal healthcare facility	NR
Kakyo et al. 2012	EPDS	Another female sexual partner husband has, current problems in marriage, parity of participants, infant's inability to breastfeed, and the husband's support were factors associated with postpartum depression	History of abortion, past mode of delivery, complications during postpartum period, self-reported health status and family support	NR	Formal healthcare facility	NR
Tsai et al. 2016	EPDS	IPV intensity had an association with depression symptom severity (regression coefficient B = 1.04; 95% CI, 0.61–1.47), with estimates from a quantile regression model showing greater adverse impacts at the upper end of the conditional depression distribution. Fitting a fixed effects regression model accounting for all time-invariant confounding (e.g., history of childhood sexual abuse) yielded similar findings (B = 1.54; 95% CI, 1.13–1.96). The magnitudes of the coefficients indicated that a one–standard-deviation increase in IPV intensity was associated with a 12.3% relative increase in depression symptom severity over the same time period	HIV status, IPV, household wealth and presence of chronic illness	NR	NA/community-based study	NR
Anderson et al. 2014	EPDS	At 6 weeks postpartum, women who had not linked to HIV care after testing positive at their first antenatal visit had higher levels of depression and internalized stigma, compared to women who had linked to care. Internalized stigma mediated the effect of linkage to care on depression. Furthermore, participants who had both linked to HIV care and initiated antiretroviral therapy reported the lowest levels of depressive symptoms	HIV status, pre- and post-IPV, and ART use	NR	Formal healthcare facility	NR
Okronipa et al. 2012	EPDS	Diarrheal incidence was 0.6 episodes/100-d at risk. More HIV-P than HIV-N and HIV-U women tended to report PND symptoms (*P* = 0.09). PND symptoms increased the risk of infantile diarrhea only for HIV-P and HIV-U but not HIV-N women (interaction term, *p* = 0.02). Health care providers should be aware of the increased risk of infantile diarrhea when both maternal HIV and PND symptoms are present and take preventive action to reduce morbidity	Maternal and infant weight, HIV status, and maternal stress	Infantile diarrhea	Formal healthcare facility	NR
Madeghe et al. 2016	EPDS	Prevalence of PPD was 13.0% (95%CI: 8.3–17.7%). Considering differences in socioeconomic status of depressed and non-depressed mothers, non-depressed mothers had a 6.14 (95%CI: 2.45–13.36) times higher odds of practicing exclusive breastfeeding than mothers who were depressed. Mothers with PPD had a 4.40 (95%CI: 1.91–11.93) time higher odds of having an underweight infant than mothers without depression	Living situation, birthweight and feeding practices	NR	Formal healthcare facility	NR
Ayele et al. 2016	BDI-II	Depression among pregnant women was 23% (95%CI: 18.48%, 26.86%). Factors significantly associated with depression were: woman`s age (20 to 29, AOR = 0.18,95% CI:0.07,0.49), occupation (housewife, AOR = 2.57,95%CI: 1.21,5.46, merchant and daily laborers, AOR = 3.44 (1.38,8.58), previous pregnancy (No, AOR = 4.74,95% CI:1.58,14.17) and previous ANC follow up pattern (irregular, AOR = 11.43,95% CI:3.68,35.49), no follow up, AOR = 11.98, 95% CI:4.73,30.33)	History of pregnancy complications, history of labor complications, number of previous abortions, planned pregnancy and number of live births, husband and community support	NR	Formal healthcare facility	NR
Lillie et al. 2020	PHQ-9	19.7% women reported symptoms indicative of moderate to severe depression with 14.1% endorsing suicidal ideation in the last 2 weeks. In the multivariable analyses, low hopefulness, household hunger, emotional IPV, physical and/or sexual IPV, and insufficient female relative support remained significantly associated with depression	Intimate partner violence, number of pregnancies, wealth quintile, social support, polygamous relationship and physical health	Suicide ideation	NA/community-based study	NR
Bisetegn et al. 2016	EPDS	Prevalence of antenatal depression was 11.8%. Having debt (OR = 2.79, 95% CI = 1.33, 5.85), unplanned pregnancy (OR = 2.39, 95% CI = (1.20, 4.76), history of stillbirth (OR = 3.97, 95% CI = (1.67,9.41), history of abortion (OR = 2.57, 95% CI = 1.005, 6.61), being in the third trimester of pregnancy (OR = 1.70, 95% CI = 1.07,2.72), presence of a complication in the current pregnancy (OR = 3.29, 95% CI = 1.66,6.53), and previous history of depression (OR = 3.48, 95% CI = 1.71,7.06) were factors significantly associated with antenatal depression	Unplanned pregnancy, hunger in the past month, debt, history of stillbirth and abortion and history of depression, social support, family history of depression and relational problems	NR	NA/community-based study	NR
Yator et al. 2017	EPDS	48% women screened positive for elevated depressive symptoms. 9% of the participants reported high levels of stigma. Multivariate analyses showed that lower education (OR = 0.14, 95% CI [0.04–0.46], *p* = 0.001) and lack of family support (OR = 2.49, 95% CI [1.14 – 5.42], *p* = 0.02) were associated with presence of elevated depressive symptoms. The presence of stigma implied more than ninefold risk of development of PPD (OR = 9.44, 95% CI [1.132–78.79], *p* = 0.04). Stigma was positively correlated with an increase in PPD	HIV status of mother and child, family/social support, substance abuse, intimate partner violence and engagement in extramarital sex	NR	Formal healthcare facility	NR
Azale et al. 2016	PHQ-9	Only 4.2% of women with high PPD symptoms had obtained mental health care and only 12.7% of women had been in contact with any health service since the onset of their symptoms. In the multivariable analysis, urban residence, adjusted odds ratio (aOR): 4.39 (95% confidence interval (CI) 1.23, 15.68); strong social support, aOR: 2.44 (95% CI 1.30, 4.56); perceived physical cause, aOR: 6.61 (95% CI 1.76, 24.80); perceived higher severity aOR: 2.28 (95% CI 1.41, 5.47); perceived need for treatment aOR: 1.46 (95% CI 1.57, 18.99); PHQ score, aOR: 1.14 (95% CI 1.04, 1.25); and disability, aOR: 1.06 (95% CI 1.01, 1.15) were associated significantly with help-seeking from health services. More than half of the women with high levels of PPD symptoms (60%) attributed their symptoms to a psychosocial cause and 269 (69.9%) perceived a need for treatment	Social support, experienced hunger, and relative wealth	NR	NA/community-based study	NR
Stewart et al. 2018	WHO SRQ-20	14.2% women scored SRQ ≥ 8, indicating likely depression. Antenatal depression was not associated with birth weight, duration of pregnancy, newborn LAZ, or head-circumference Z-score. There was an inverse association with newborn MUAC (adjusted mean difference − 0.2 cm (95%CI: − 0.4–0, *p* = 0.021)	HIV status, number of pregnancies, and duration of pregnancy	NR	Formal healthcare facility	NR
Garman et al. 2019	EPDS	Four trajectories were identified: chronic low (71.1%), late postpartum (10.1%), early postpartum (14.4%), and chronic high (4.5%). Low social support, unwanted pregnancy, and risky drinking were associated with the chronic high trajectory; unemployment and HIV-positive status with the early postpartum trajectory; and intimate partner violence with the late postpartum trajectory. Weight-to-length and weight-for-age z-scores at 18 months, and weight-for-age z-scores, length-for-age z-scores, emotional symptom, and peer problem scores at 36 months differed across trajectories	Intimate partner violence, social support, unplanned pregnancy, substance use and history of miscarriage	NR	NA/community-based study	NR
Nyamukoho et al. 2019	EPDS	39.4% (95%CI: 32.5–46.3) met criteria for antenatal depression Intimate partner violence (IPV) [OR 3.2 (95% CI 1.5–6.7)] and previous history of depression OR 4.1 (95% CI 2.0–8.0)] were associated with depression	HIV status, intimate partner violence, childhood sexual abuse, social support, history of depression and miscarriage and presence of chronic condition	NR	Formal healthcare facility	NR
Duko et al. 2019	EPDS	Prevalence of antenatal depression was 21.5%. Being in age group of 20–30 years [AOR = 5.85 (95% CI: (3.70, 10.14)], current pregnancy complication [AOR = 4.98 (95% CI: (3.01, 10.37)], unplanned pregnancy [AOR = 7.12, (95% CI: (3.12, 9.63)], categories of stressors (LTE) Health risk [AOR = 1.76, (95% CI: (1.01, 3.22)], previous history of depression [AOR = 2.76 (95% CI: (1.94, 6.75)], history of abortion [AOR = 1.52, (95% CI:1.04, 5.09)], history of still birth [AOR = 1.18, (95% CI: 1.08, 2.91)], poor social support [AOR = 2.14, (95% CI: 1.49, 3.11)] and poor baby father support [AOR = 3.21 (95% CI:1.93, 6.71)] were significantly associated with antenatal depression	Current and previous pregnancy complications, history of abortion and child loss, partner and social support, unplanned pregnancy, and history of stilbirth	NR	Formal healthcare facility	NR
Belay et al. 2019	EPDS	Prevalence of antenatal depressive symptom was 6.8% (95% CI 6.2–11.3). Increased risk of depression was found among women who had been exposed to IPV (AOR = 17.60; 95%CI = 6.18–50.10) and whose husbands use alcohol (AOR = 3.31; 95%CI = 1.33–8.24)	Partner's substance use, social support, parental violence, and pregnancy desire	NR	NA/community-based study	NR
Garman et al. 2019	EPDS	Two trajectories were identified: antenatal only (91.4%), with moderate to severe symptoms at baseline which later subside; and antenatal and postnatal (8.6%), with severe depressive symptoms during pregnancy and later in the postpartum period, which subside temporarily to moderate levels at 3 months postpartum. Predictors for the antenatal and postnatal trajectory include severe food insecurity, intimate partner violence, lower social support, greater functional impairment, problematic drinking and suicide risk	Intimate partner violence, social support, substance use, HIV status, history of depression, and food status	NR	NA/community-based study	NR
Kerie et al. 2018	EPDS	33.82% mothers had postpartum depression. Unplanned pregnancy adjusted odds ratio (AOR) = 4.49, 95% CI (2.31, 8.71), age from 15 to 24 years AOR = 0.420, 95% CI (0.18, 0.98), having a chronic physical illness AOR = 7.71, 95% CI (2.34, 25.44), experiencing death of infant AOR = 4.12, (1.78, 9.51) and unstable marital condition AOR = 6.02, (2.79, 12.99) were significantly associated with postpartum depression	Unplanned pregnancy, chronic illness, history of depression, death of infant, current marital problem, and mode of delivery	NR	Formal healthcare facility	NR
Harrington et al. 2020	EPDS	9.5% (95%CI 7.5–11.9%) of women screened positive for current depression, and 46% self-reported a history of depression or anxiety. Women were more likely to screen positive for current depression if they reported a history of depression (adjusted PR 2.42; 95%CI 1.48–3.95) or had ever experienced intimate partner violence (1.77; 1.11–2.81). Having an unintended current pregnancy (1.78; 0.99–3.21), being unmarried (1.66; 0.97–2.84), or employed (1.56; 1.00–2.44) had potential associations with probable depression	Intimate partner violence, history of depression or anxiety, family history of depression or anxiety, HIV clinical stage, previous child death, disclosure of HIV status	NR	Formal healthcare facility	NR
Fantahun et al. 2018	EPDS	Prevalence of postpartum depression among mothers was 23.3%. Moreover, women who were unmarried, had unplanned pregnancy, delivered without presence of any relatives in health institutions, had previous history of child health, had history of substance use and had low income were found to more often display postpartum depression	Number of pregnancies, planned pregnancy, sex of last baby, desired sex of baby, stressful life events, history of child death, illness during pregnancy, and hospitalization of child(ren)	NR	Formal healthcare facility	NR
Mokhele et al. 2019	CES-D	25.0% women screened positive for postpartum depression; 14.6% women had low to medium PPD 10.4% had major PPD. A higher proportion of HIV-negative women experienced PPD, 129/461 (28.0%) among HIV negative vs.159/690 (23.0%) among HIV-1 infected. Among HIV positive women, there was no mean- ingful difference in PPD between newly HIV diagnosed and those diagnosed before the most recent pregnancy (aOR 1.3, 95% confidence interval (CI): 0.9–1.8). Predictors of PPD among HIV positive women were living with friends/in a house-share (aOR 0.5 for house- share vs. own home, 95% CI: 0.3–0.9), and attending antenatal care (ANC) for the most recent pregnancy (aOR 0.2 for ANC attendance vs. no ANC attendance, 95% CI: 0.0–0.5). Living with friends/in a house-share was also a predictor of PPD among HIV-negative women (aOR 0.4 for house-share vs. own home, 95% CI: 0.2–0.8)	Social support, HIV knowledge, family support, sex of newborn, and planned pregnancy	NR	Formal healthcare facility	NR
Woldetensay et al. 2018	PHQ-9	Community based prevalence of depressive symptoms during pregnancy was 10.8% (95%CI: 9.92–11.70). Moderate household food insecurity (OR 1.74; 95% CI: 1.31–2.32), severe household food insecurity (OR 7.90; 95% CI: 5.87–10.62), anemia (OR = 1.30; 95% CI: 1.04–1.61) and intimate partner violence (OR 3.08; 95% CI: 2.23–4.25) were significantly associated with prenatal depressive symptoms. On the other hand, good social support from friends, families and husband reduced the risk of prenatal depressive symptoms by 39% (OR 0.61; 95% CI: 0.50–0.76)	History of child death, social support, intimate partner violence, anemia and household food insecurity, history of abortion, acute illness and social participation	NR	NA/community-based study	NR
Naude et al. 2022	BDI-II	Antenatal depressive symptoms (25%) were significantly associated with higher levels of IL-7 (*p* = 0.008), IL-8 (*p* = 0.019), and TNF-α (*p* = 0.031) in the mothers after correcting for sociodemographic and lifestyle factors. Serum IL-1β and NGAL levels were significantly elevated over time in children born to mothers with depressive symptoms compared to those without depression, after controlling for maternal and child health and sociodemographic factors. Elevated infant IL-1β at 6–10 weeks of age partially mediated the association of maternal depressive symptoms with poorer language scores at 2 years	Sociodemographic and lifestyle factors (maternal smoking during pregnancy, maternal alcohol use during pregnancy, maternal socioeconomic status, maternal BMI at 6 weeks postpartum, and maternal HIV status) and infant health	Poor language development	2 primary healthcare clinics	NR
Silverman et al. 2021	WHO SRQ-20	Increased reporting of poor mental health symptoms at 7 months postpartum as compared to shortly after birth. Worsening maternal mental health over the postpartum period was associated with higher SRQ-20 symptom scores shortly after birth	Socioeconomic resources and preexisting depressive/anxiety symptoms	NR	NR	NR
Boateng et al. 2022	CES-D	Interaction effect of Food Insecurity, Water Insecurity, and HIV was significantly associated with greater depressive symptomatology (β = 0.06) at 21 months postpartum	Water insecurity, food insecurity, and HIV	NR	7 Family AIDS Care and Educational Services (FACES) clinics	NR
Kariuki et al. 2022	BDI-II	Prevalence of PND in the sample of women was 27.1%. Women aged 18–24 (β = 2.04 95% CI [0.02; 4.05], *p* = 0.047), dissatisfied with body image (β = 4.33 95% CI [2.26; 6.41], *p* < 0.001), had an unplanned pregnancy (β = 2.31 95% CI [0.81; 3.80], *p* = 0.003 and felt fatigued (β = − 1.85 95% CI [− 3.50; 0.20], *p* = 0.028) had higher odds of developing PND. Participants who had no stressful life events had significantly lower depression scores as compared to those who had stressful life events (β = − 1.71 95% CI [− 3.30; − 0.11], *p* = 0.036) when depression was treated as a continuous outcome. Sensitivity analysis showed that mothers who had secondary and tertiary level of education had 51 and 73% had lower likelihood of having depression as compared to those with a primary level of education (A.O.R = 0.49 95% CI [0.31–0.78], *p* = 0.002) and (aOR = 0.27 95% CI [0.09–0.75], *p* = 0.013), respectively	Age in years, level of education, income, employment, body image, stress, planned pregnancy	NR	Health Centers/MCH clinics	NR
Soyemi et al. 2022	NR	In the first trimester of pregnancy, the prevalence of depression was 10.5%, while it was 3.9% in the third trimester of pregnancy. Collectively, the relationship between depression and QoL was significant in the overall domain	Depressive symptoms and quality of life	Poor quality of life	Antenatal clinic/hospital	NR
Osborn et al. 2021	PHQ-9	In pregnant WLWH, 9% had depression; these women had more recent HIV diagnosis than those without depression. Depression was associated with HIV-related stigma (adjusted Prevalence Ratio [aPR]: 2.36, *p* = 0.025), IPV (aPR:2.93, *p* = 0.002), and lower social support score (aPR:0.99, *p* = 0.023). Using population-attributable risk percent to estimate contributors to maternal depression, 81% were attributable to stigma (27%), recent diagnosis (24%), and IPV (20%)	HIV status, partner violence, stigma, gestational age and unintended pregnancies	NR	6 public maternal and child health clinics	NR
Stress/distress, psychological distress, anxiety, or posttraumatic disorder						
Ngene and Moodley 2021	Four-items perceived stress scale (PSS)	In the normotensive group, there was a positive correlation between sFlt- 1 and postpartum PSS and between sFlt- 1/PIGF ratio and post-partum PSS	Placental growth factor (PIGF), soluble fms-like tyrosine kinase (sFlt-1) and their ratio (sFlt-1/PGIF)	NR	Formal healthcare facilities (a regional hospital)	NR
Odinka et al. 2018	Hospital Anxiety and Depression Scale (HADS)	Prevalence of postpartum anxiety was 31.1% and of PPD was 33.3%. Marital dissatisfaction was observed in 39.5% (122) of the respondents who were mothers. Those with co-morbid depression and anxiety (22.0%) had worse marital dissatisfaction	HADS Depression subscale was positively related to index of marital satisfaction	NR	Hospital	NR
Shifa et al. 2018	WHO’s SRQ-20 (≥ 6)	Mothers who lost children had a significantly higher rate of mental distress (adjusted odds ratio (AOR) of 1.84(1.11–3.04) compared to their counterparts. Similarly, mothers with child loss reported a significantly higher rate of suicidal ideation (23.3%) than mothers without child death (16.3%), with p-value of 0.003	Odds of mental distress among mothers with child death among those mothers without child death (adjusted odds ratio (AOR) of 1.84 (1.11–3.04). Wealth: Average (AOR of 0.41(0.21–0.79)) and rich (AOR of 0.31 (0.14–0.67)) wealth indices; no other medical problems (AOR of 0.36 (0.18–0.73); seeking medical care from modern health facilities (AOR of 0.43 (0.20–0.94); ANC follow up during pregnancy of the index child, (AOR of 2.55 (1.25–5.18)	NR	Hospital	NR
MacGinty et al. 2017	Modified Post-Traumatic Stress Disorder Symptom Scale (MTSDSS)	Postnatal psychological distress was associated with both presences of wheezing and recurrent child wheezing	NA	Presence of wheeze (adjusted OR = 2.09, 95%CI: 1.16–3.77), and recurrent child wheeze (AOR = 2.26, 95%CI: 1.06–4.81)	NR	NR
Wall et al. 2017	Pregnancy related Anxiety Questionnaire (PRA-Q) and EPDS	25% of women scored 13 or higher (out of a possible 30) on the PRA-Q. Perceived stress, active depression and the number of people living in the home was the only statistically significant predictors of PRA	Perceived stress, active depression, and a number of people living in the home were associated with pregnancy-related anxiety	NR	District ANC facility	NR
Jebena et al. 2015	WHO SRQ-20	22.4% had mental distress. Food insecurity was also associated with mental distress. Pregnant women living in food-insecure households were 4 times more likely to have mental distress than their counterparts	Food insecurity (AOR = 4.15, 95% CI: 1.67, 10.32)	NR	Antenatal clinics	NR
Stewart et al. 2014	Focused group discussion (FGD) guide	Three major themes were identified: pregnancy as a time of uncertainty, the husband (and others) as support and stressor, and the impact of stressors on mental health. Poverty, lack of support, HIV, witchcraft, and child illness were identified as causes of worry in the perinatal period	Poverty, lack of support, HIV, witchcraft, and child illness	NR	NR	NR
Abegaz et al. 2022	Pregnancy-Related Anxiety Questionnaire-Revised (PRAQR)	Prevalence of pregnancy-related anxiety was found to be 43.9%. Having no formal education, primigravida, intimate partner violence and poor social support were associated with pregnancy-related anxiety	Depression, intimate partner violence, social support, history of mental illness	NR	Formal healthcare facility	NR
Ogueji 2021	Kessler psychological distress scale (K6)	The mean score on psychological distress was 17.07. Qualitative analysis found ‘anxious, depressive reports, loneliness, and regrets’, self-blame and guilt feelings’, as the experiences of psychological distress	Loneliness, self-blame and guilt	NR	Formal healthcare facility	NR
Wycliffe et al. 2021	NR	Factors contributing to maternal emotional distress were low levels of education, unemployment, lengthy NBU stays, ineffective communication patterns, null communication between mothers and health providers	Length of hospital stay and poor communication	NR	Formal healthcare facility	NR
Bishaw et al. 2022	GAD-7	Prevalence of generalized anxiety disorder was 43.7%. Having < 3 number of children, having a negative attitude about COVID and having a high- risk perception about COVID-19 were factors associated with generalized anxiety disorder	Good practice, favorable attitude and knowledge on COVID-19	NR	Formal healthcare facility	NR
Malaju et al. 2022	Traumatic Event Scale (TES)	Perceived traumatic childbirth, fear of childbirth, depression, anxiety, psychological violence, higher WHODAS 2.0 total score, multigravidity, stressful life events of health risk, relational problems and income instability were found to be predictors of PTSD	Social support, stressful life events, domestic violence, fear of childbirth, and functional status	NR	Formal healthcare facility	NR
MacGinty et al. 2020	WHO SRQ-20	21% of women reported psychological distress. Antenatal psychological distress was associated with childhood trauma, antenatal depression and smaller head circumference at birth	Intimate partner violence, childhood trauma, substance use, mental disorders, partner support, and birth and developmental outcomes	Childhood trauma and smaller head circumference	Formal healthcare facility	NR
Akinsulore et al. 2021	Perinatal anxiety screening scale	Prevalence of anxiety was 43.5%. Maternal age and socio-medical worries were associated with anxiety among pregnant women	Pregnancy complications, history of miscarriage, and partner support	NR	Formal healthcare facility	NR
Kassaw et al. 2020	GAD-7	Prevalence of generalized anxiety disorder was 32.2%. Living in a rural area, primary level of education, poor social support, and primigravida were factors associated with generalized anxiety disorder	Previous mental condition, social support, pregnancy status	NR	Formal healthcare facility	NR
Koen et al. 2017	MNI and DSM–IV	Most mothers (72%) reported lifetime trauma exposure; the lifetime prevalence of PTSD was 20%. Maternal PTSD was significantly associated with poorer fine motor and adaptive behavior–motor development; the latter remained significant when adjusted for site, alcohol dependence, and infant head-circumference-for-age z-score at birth	Stressful life events, substance use, trauma during childhood, and intimate partner violence	Poorer fine motor and adaptive behavior–motor development of child	Formal healthcare facility	NR
Nöthling et al.^ 2013^	CES-D	Children of mothers with depression were significantly more likely to display total behavior problems than children of mothers without depression. Maternal PTSD had greatest explanatory power for child behavior problems, although it did not predict child outcomes	CD4 count, substance use, experienced life events, external behavior problems	Behavior problems (attention and aggression)	Formal healthcare facility	NR
Guo al. 2014	GAD-7 and PHD-9	Parenting stress (PS) assessment at 3 months, 12 months and 24 months postpartum and the prevalences at each time point were 33.1%, 24.4%, and 14.9% in Ghana and 30.2%, 33.5%, and 22.6% in Côte d’Ivoire, respectively. At all three time points, the PS scores were significantly higher among depressed mothers vs. non-depressed mothers. In the multivariate regression analyses, antepartum and postpartum depression were consistently associated with PS after adjusting for other variables	Parental distress and parent–child dysfunctional interaction	NR	Formal healthcare facility	NR
Bekele et al. 2017	WHO SRQ-20	Prevalence MD (SRQ-20 score > 6) was found to be 26.2% (95%CI: 23.04 −29.36). Women with unplanned pregnancy, with obstetric problems in their current pregnancy and with history of psychiatric illness in the past were found to have a significantly higher MD	Planned pregnancy, history of abortion, obstetric complications and history of death of children	NR	Formal healthcare facility	NR
Koen et al. 2016	MINI and DSM–IV	Lifetime trauma was reported in approximately two-thirds of mothers, with about a third exposed to past-year intimate partner violence (IPV). The prevalence of current/lifetime PTSD was 19%. In multiple logistic regression, recent life stressors were significantly asso- ciated with lifetime trauma, when controlling for SES, study site, and recent IPV. Childhood trauma and recent stressors were significantly associated with PTSD, controlling for SES and study site. While no association was observed between maternal PTSD and birth outcomes, maternal trauma was significantly associated with a 0.3-unit reduction (95% CI: 0.1; 0.5) in infant head-circumference-for-age z-scores (HCAZ scores) at birth in crude analysis, which remained significant when adjusted for study site and recent stressors in a multivariate regression model	Stressful life events, substance use, trauma during childhood, and intimate partner violence	NR	Formal healthcare facility	NR
Chingono et al. 2021	Shona Symptom Questionnaires	Involvement in interactive self-help groups improved their mental health by strengthening peer support and engendering hope for the future	Burden of motherhood, social stigma and isolation related to sex work, and self-help groups as a source of hope	NR	NR	NR
Lelisho et al. 2022	GAD-7	Prevalence of generalized anxiety disorder (GAD) among women attending perinatal service during COVID-19 were 31.7%, 40.4%, 20.1%, and 7.8% had minimal to severe generalized anxiety disorder, respectively	Being town resident, lower-income status, occupation status, having a chronic illness, having a positive family history of anxiety or mood disorder, perceived social support, and fear of the COVID-19	NR	4 hospitals	NR
Self-harm and suicide/suicidal ideation						
Tilahun et al. 2022	Suicidal screening tool (Mini-International Neuropsychiatric Interview (MINI))	Magnitude or prevalence of suicidal behavior was 41.46%. Verbal abuses, a history of rape and depression, having a sexually unfaithful husband and khat chewing were factors in suicidal behaviors	Social support, substance use, abuse, having a sexually unfaithful husband, and mother's current wealth status	NR	10 formal healthcare facilities	NR
Rodriguez et al. 2018	EPDS-10	Prenatal suicidal ideation was 39%; suicidal ideation continued for 7% at 12 months, 13% experienced incident suicidal ideation, and for 19% suicidal ideation had stopped postnatally. Intimate partner violence (AOR = 1.17) and depression (AOR = 1.14) predicted sustained suicidal ideation. Increased income (AOR = 2.25) and greater stigma (AOR = 1.33) predicted incident suicidal ideation. Younger age (AOR = 0.94), disclosure of HIV status to partner (AOR = 0.60), and greater stigma (AOR = 1.24) predicted postnatal cessation of suicidal ideation	Suicidal ideation at baseline and 12 months: Physical intimate partner voilence (IPV) (AOR) = 1.17 [1.02, 1.34]). Depression: IPV– > (AOR = 1.14 [1.09, 1.19]). Suicidal ideation at 12 months: Increased income (AOR = 2.25 [1.17, 4.36) and greater stigma (AOR = 1.33 [1.06, 1.67). Cessation of suicidal ideation at 12 months: younger age (AOR = 0.94 [0.90, 0.99), greater stigma (AOR = 1.24 [1.02, 1.46), disclosure of HIV status to partner (AOR = 0.60 [0.36, 0.94)	NR	NR	NR
Onah et al. 2017	Suicidal ideation: Risk Factor Assessment (RFA) tool developed by the Perinatal Mental Health Project. Mental disorders: Expanded MINI Plus Version 5.0.0	The 1-month prevalence of SIB was 18%. SIB was associated with psychiatric illness, notably major depressive episode (MDE) and any anxiety disorder. However, 67% of pregnant women with SIB had no MDE diagnosis, and 65% had no anxiety disorder, while 54% had neither MDE nor anxiety disorder diagnoses. Factors associated with SIB included lower socio-economic status, food insecurity, interpersonal violence, multiparousity, and lifetime suicide attempt	Suicidal ideation: Being in a casual, unmarried relationship [aOR 3.69, 95% CI 1.17–11.65]; SIV [aOR 2.14, 95% CI 1.10–4.19]; > 2 living children [aOR 2.49, 95% CI 1.90–6.82]; women experiencing major depressive episode [aOR 1.86, 95% CI 1.69–3.65] Suicidal behavior: Women who experienced IPV [aOR 2.41, 95%CI 1.79–7.35]; food insecurity [aOR 3.98, 95% CI 1.52–10.41]	NA	NR	NR
Abdelghania et al. 2021	Beck Scale for Suicide Ideation, Zagazig depression scale, Hamilton anxiety scale and Structured Clinical Interview for DSM-IV-TR Axis II	Prevalence of CSR, suicidal ideation, and attempts were 23.4%, 21.6% and 1.8%, respectively. Predictors of CSR were history of IPV exposure, identification of current pregnancy as female baby, previous history of fetal loss, and moderate-to-severe depression	Comorbid depression, anxiety and personality disorders	NR	Formal health facility	NR
Belete et al. 2021	World Mental Health (WMH) Survey Initiative Version of the World Health Organization (WHO) Composite International Diagnostic Interview (CIDI)	Prevalence of suicide ideation and attempt were 11.8% and 2.7%, respectively. Unplanned pregnancy, poor social support, common mental disorders, and lifetime suicide ideation were factors associated with suicide ideation. Social support correlated with suicide attempts among pregnant mothers	Intimate partner violence, social support, unplanned pregnancy	NR	Formal healthcare facility	NR
Redinger, et al. 2021	EPDS	18% reported thoughts of self-harm at some stage during pregnancy. Prevalence of TSH was slightly higher in early pregnancy (12.5%) than later in pregnancy (11.6%). TSH were associated with a history of mental illness, concurrent depression, marital stress, and practical support	Depression and anxiety	NR	Formal healthcare facility	NR
Knettel et al. 2020	EPDS and PHQ-9	Suicidal ideation was endorsed by 12.8% of women during pregnancy and decreased significantly to 3.9% by 6 months postpartum. women experiencing anxiety and HIV stigma were significantly more likely to endorse suicidal ideation during pregnancy	Anxiety, HIV status, acceptance and stigma, disclosure of HIV status, social support and interpersonal violence	NR	Formal healthcare facility	NR
Psychosis						
Belete et al. 2021	MINI and DSM–IV	Prevalence of suicidal behavior (suicidal ideation, plan or suicide attempt) was 14.0% (138/988) (95% CI 12.00 to 16.00) in postpartum mothers. Poor wealth of the mother (AOR = 2.80, 95%CI 1.18–6.84), unplanned pregnancy of the current child (AOR = 2.28, 95%CI 1.48–3.54), history of rape (AOR = 2.26, 95%CI 1.42–3.61) and sickness of the new child (AOR = 1.68, 95%CI 1.12–2.52) were significantly associated with suicidal behaviors	Age at marriage, history of rape and abortion, intimate partner violence, social support, unplanned pregnancy, history of depression, substance use of partner, and sexually unfaithful husband	NR	Formal healthcare facility	NR
Adjorlolo et al. 2022	PHQ-9, GAD-7, and prodromal questionnaire	54.2%, 27.3% and 18.5% of pregnant women had no/low, moderate and high-risk psychosis, respectively. Women more concerned about COVID-19 effects, had high suicidal ideation score, had depressive symptoms and sleep difficulties were linked to psychosis	Emotional assistance, Intimate partner violence, suicidal ideation, sleeping difficulties, and COVID-19 concerns	NR	Formal healthcare facility	NR
Multiple and common mental health disorders						
Rurangirwa et al. 2018	MINI version 5.0.0	The prevalence rates of generalized anxiety disorder, suicide ideation and post-traumatic stress disorder (PTSD) were 19.7%, 10.8% and 8.0%, respectively. Exposure to the four forms of IPV during pregnancy was highly associated with the likelihood of meeting diagnostic criteria for each of the non-psychotic MHDs investigated. Physical, psychological, and sexual violence showed the strongest association with PTSD, with adjusted ORs (aORs) of 4.5, 6.2 and 6.3, respectively. Controlling behavior had the strongest association with major depressive episode in earlier periods with an aOR of 9.2	For physical, psychological, and sexual violence: aORs of 4.52 (95% CI: 2.14 to 9.58), 6.29 (95% CI: 3.18 to 12.46), and 6.22 (95% CI: 2.98 to 12.92), respectively. Suicidal ideation: with aORs of 3.16 (95% CI: 1.53 to 6.52), 2.89 (95% CI: 1.51 to 5.55) and 3.04 (95% CI: 1.40 to 6.60) for exposure to physical, sexual, or psychological violence, respectively	NA	NR	NR
Baumgartner et al. 2016	WHO SRQ-20	Poor health of the last delivered child and inequitable gender attitudes were associated with poor mental health among other factors. Social support from female friends was strongly protective	Poor health of the last delivered child and inequitable gender attitudes were associated with poor mental health among other factors. Social support from female friends was strongly protective	NR	NR	NR
Guo et al. 2014	PHQ-9 and Generalized Anxiety Disorder-7 (GAD-7)	Parenting scores were significantly higher among depressed mothers vs. non-depressed mothers. In the multivariate regression analyses, antepartum and postpartum depression were consistently associated with PS after adjusting for other variables	NR	Parenting stress	Antenatal clinics	NR
Baumgartner et al. 2014	WHO SRQ-20	On SRQ-20, 32.8% had probable CMD using the 4/5 cutoff score versus 19.8% using the more conservative 7/8 cutoff. About 15% of the women responded affirmatively that they had had suicidal thoughts	Unmarried women were significantly more likely than married women to experience poor mental health (*P* < 0.0001)	NR	NR	NR
Ade-Ojo et al. 2022	PHQ-9 and GAD-7	Major depressive disorder (MDD) and severe anxiety disorder were significantly higher among the HIV-positive group than in the HIV-negative group	Socio-demographic data, depression, and anxiety	NR	Formal health (Government) facilities	NR
Abrahams et al. 2022	EPDS	Prevalence of CMD was 12.5%. Psychological distress increased during the lockdown period, compared to before the COVID-19 outbreak	Intimate partner violence and food insecurity	NR	Formal healthcare facility	NR
Abdelhai and Mosleh 2015	HADS	Prevalence of anxiety and depression were 11.4% and 10.4%, respectively. Exposure to domestic violence was associated with anxiety and depression	Financial distress	NR	Formal healthcare facility	NR
Ndukuba et al. 2015	ICD	Schizophrenia was commonest, 48.7% (37/76) followed by depression, 22% (17/76), and mania, 15% (11/76). Those presenting with schizophrenia were younger when compared with other diagnostic groups, had a lower level of education, and presented earlier for treatment	NR	NR	Antenatal clinics	NR
Abrahams et al. 2022	EPDS	Experiencing domestic violence was associated with higher scores of common mental disorders	Domestic violence, abuse, financial support, sleep problems, psychological distress and presence of chronic infection	NR	Formal healthcare facility	NR
Agbaje et al. 2019	EPDS and HADS-A	Prevalence of depression and anxiety was 34.6% and 33.3%, respectively. History of depression and being a mother aged 15–29 years had association with anxiety. Attendance at postnatal care services was significantly associated with depression	History of depression and socio-demographic characteristics	NR	Formal healthcare facility	NR
Parcesepe et al. 2021	WHO SRQ-20	Prevalence of common mental disorders was 42%. Emotional, physical, and sexual IPV were reported by 44, 37, and 31% of respondents, respectively. All forms of IPV were associated with greater odds of CMD	Intimate partner violence	NR	Formal healthcare facility	NR
Bante et al. 2021	PHQ-9 and GAD-7	Prevalence of anxiety-depression comorbidity was 10%. Being married, highest wealth quintile, medical illness, encountering pregnancy danger signs, experiencing life-threatening events and household food insecurity were significantly associated with CAD	Social support, substance use, medical illness, household food insecurity, experience life-threatening events, and presence of danger signs in pregnancy	NR	NA/community-based study	NR
Jidong et al. 2021	NR	Mothers experienced persistent psychological distress from time of labour/birth and anxiety due to limited knowledge about childcare, access to support and healthy food	NR	NR	Formal healthcare facility	NR
Tamiru et al. 2022	WHO SRQ-20	Prevalence of common mental disorders was 37.5%. Current substance use, intimate partner violence, null parity, gestational age [first trimester and third trimester, history of abortion, and absence of antenatal care follow-up were associated with common mental disorder	Intimate partner violence, history of mental illness and chronic illness, substance use, history of gynecological depression	NR	NA/community-based study	NR
Kugbey et al. 2021	HADS	Monthly income, insomnia, non-nutritious food consumption (pica), and body image satisfaction were associated with depression. Marital status, insomnia, lifetime suicidal behavior and partner support were associated with anxiety. Partner abuse was associated with suicidal behavior	Intimate partner violence, partner support, food insecurity, substance use and loneliness	NR	Formal healthcare facility	NR
Nwafor et al. 2021	Depression Anxiety and Stress Scale-21 (DASS-21)	Severe and extremely severe depression were reported in 7.2% and 6.4%, respectively. A 3.3% and 7.7% of women had severe and extremely severe anxiety, respectively. In total, 23% of the women had severe stress while 16.7% reported extremely severe stress. Multiparity (2–4) and occupation, such as trading and farming, were predictors of depression whereas grand-multiparity, urban residence, and trading, were predictors of anxiety and stress	NR	NR	Formal healthcare facility	NR
Barsisa et al. 2021	WHO SRQ-20	Prevalence of common mental disorders was 36.6%. Being single/divorced/widowed, having a chronic medical illness, exposure to two/more stressful events, poor social support, mothers living with cigarette smoker husbands and mothers physically abused by spouses were factors associated with a common mental disorder	History of substance use, social support and life-threatening event	NR	Formal healthcare facility	NR
Masiano et al. 2022	WHO SRQ-20	About 52% and 44% reported anxiety and depressive thoughts, respectively. Adverse childhood experiences were associated with depressive symptoms	NR	NR	Formal healthcare facility	NR
Khan et al. 2020	MINI	36.5% and 5.7% prevalence of CMD and suicide risk, respectively	HIV status and presence of chronic illness	NR	Formal healthcare facility	NR
Umuziga et al. 2020	EPDS (depression) and Zung Self-rating Anxiety Scale (anxiety)	37.6% women had depression and 28.2% had anxiety. Among postnatal women, 63.6% had depression, and 48,1% had anxiety. Having four or more living children and poor relationships with partner was associated with depression. Lifetime exposure to stressful events was the only predictor of anxiety	Planned pregnancy and stressful life event	NR	Formal healthcare facility	NR
Ola et al. 2011	WHO SRQ-20	7% met the criteria for experiencing a common mental disorder. Presence of interpersonal violence predicted being a case of mental illness (OR = 3.400; 95%CI = 1.374—8.414 and OR = 5.676; 95% CI = 1.251—25.757 respectively)	Partner support, history of physical violence and substance use, and planned current pregnancy	NR	Formal healthcare facility	NR
Ngocho et al. 2019	EPDS and Brief Symptom Index (BSI-18)	25.0% and 23.5% of women had depression and anxiety. Depression was associated with being single, food insecurity, and HIV shame. Anxiety was associated with being single, HIV shame and lifetime experience of violence. 17.8% had comorbidity of depression and anxiety. Comorbid depression and anxiety was associated with being single HIV shame and lifetime experience of violence	Intimate partner violence, enacted stigma, HIV shame, food security, social support, and partner's HIV status	NR	Formal healthcare facility	NR
Woldetsadik et al. 2019	WHO SRQ-20	Prevalence of CMD during pregnancy was 35.8% (95% CI: 34–38%) and the main determinants of CMD were: illiteracy, presence of health risk, financial instability, physical or emotional abuse, having sexual intercourse without her willingness, family history of psychiatric illness and history of chronic medical illness	Planned pregnancy, history of stillbirth and abortion, history of neonatal death and pregnancy complication	NR	NA/community-based study	NR
Stewart et al. 2014	WHO SRQ 20; DSM-IV	Adjusted weighted prevalence of current major depressive episode and current major or minor depressive episode were 10.7% (95%CI: 6.9–14.5%) and 21.1% (95% CI 15.5–26.6%), respectively. On multivariate analysis, SRQ score was significantly associated with lower perceived social support, experience of intimate partner violence, having had a complication in a previous delivery, higher maternal mid-upper arm circumference and more years of schooling. Major depressive episode was associated with lower perceived social support and experience of intimate partner violence	Social support, complications during pregnancy, HIV status of mother, intimate partner violence and partner support	NR	Formal healthcare facility	NR
Bindt et al. 2012	PHQ-9; GAD WHO Disability Assessment Schedule II (WHO-DAS 2.0)	In Ghana, 26.6% of women showed substantially depressed mood. In Cote d’Ivoire, this figure was even higher (32.9%). Clear indications for a generalized anxiety disorder were observed in 11.4% and 17.4% of pregnant women, respectively. Comorbidity of both conditions was common, affecting about 7.7% of Ghanaian and 12.6% of Ivorian participants. Pregnant women in both countries reported a high degree of disability regarding everyday activity limitations and participation restrictions. Controlled for country and age, depression and anxiety accounted for 33% of variance in the disability score	NR	NR	Formal healthcare facility	NR
Shamu et al. 2016	CED-S	21.4% of participants were diagnosed with depression whilst 21.6% reported postpartum suicide thoughts and 4% reported suicide thoughts. IPV was associated with postnatal depression and emotional IPV were associated with suicidal ideation	Past violence, relationship status, HIV status, past suicidal ideation and pregnancy outcome	NR	Formal healthcare facility	NR
Bindt et al. 2013	PHQ-9 and GAD-7	Prevalence of depression and anxiety symptoms were 28.9% and 14.2% respectively. Mean birth weight was 3172.1 g (SD 440.6) and the prevalence of LBW was 1.7%. The mean gestational age was 39.6 weeks and the proportion of PTB was 4%. Multivariate linear regression revealed no significant association between maternal depression (B = 52.2, 95%CI −18.2 122.6, *p* = 0.15) or anxiety (B = 17.1, 95% CI −74.6 108.7, *p* = 0.72) and birth weight. Yet, low socio-economic status, female sex of the child, and younger maternal age were associated with lower birth weight. Multivariate logistic regression suggested no significant association between maternal depression (OR: 2.1, 95%CI 0.8 5.6, *p* = 0.15) or anxiety (OR: 1.8, 95%CI 0.6 5.5, *p* = 0.29) with PTB	Previous pregnancy complications, infections, substance use, infant sex, and mode of delivery	NR	Formal healthcare facility	NR
Boakye-Yiadom et al. 2015	CES-D and State Trait Anxiety Inventory (STAI)	Mean ± SD of anxiety score as well as stress score from the studied population were 15.3 ± 3.2 and 13.2 ± 4.9 respectively. The prevalence of anxiety was 9.7% while that for stress among these studied participants was 28.6%. Higher proportion (26.6%) of educated women had anxiety disorders with a lower (21.9 ± 10.8) mean gestation period was associated with anxiety disorders. Age was higher (28.1 ± 5.8; *p* = 0.0155) in women with pregnancy-specific stress than in normal women (25.0 ± 7.9). A higher proportion of women who were married (99.1%; 0.0097) were normal as compared to those who had pregnancy stress (90.9%). Mean number of births was seen to be higher among normal women (4.3 ± 5.9; 0.0054) than those with stress (1.8 ± 1.4). This study reiterates the rising levels of pregnancy-specific stress and anxiety, with social and medical factors such as literacy levels, gestational period, age, marital status and parity playing major roles in the determination of pregnancy related stress and anxiety levels	Complications during pregnancy, number of births, duration of marriage and gestation age	NR	Formal healthcare facility	NR
Roos et al	Spielberger trait inventory for anxiety and K-10	Predictors of distress and anxiety were lower self-directedness, higher harm avoidance, trait anxiety, lower resilience and social support	Perceived social support, resilience, temperament and trait anxiety	NR	Formal healthcare facility	NR
Wassif et al. 2019	DASS-42	1.6% of the studied females suffered from postpartum depression alone, 10% suffered from anxiety alone, and 21.2% suffered from both. The mean age of female who suffered from comorbid depression and anxiety was significantly (*p* = 0.01) higher than the normal group (26.9 and 25.1, respectively), and they had a significantly lower socioeconomic score than the normal ones (31.1 and 34.1, respectively), *p* < 0.05. There was a significant association (*p* < 0.001) between the past history of similar conditions and the current prevalence of postpartum disorders. Progesterone level ≤ 4.6, ≤ 11.3, and ≤ 2.8 significantly predict depression alone, anxiety alone, and comorbid diseases, respectively	Gestational age, sex of the born child, iron intake during pregnancy, anemia during pregnancy, and history of depression	NR	Formal healthcare facility	NR
Pobee et al. 2022	CES-D and Beck Anxiety Inventory	Prevalence of psychosocial disturbances was 34%, 10%, 2% (anxiety), 49%, 31%, 34% (depressive symptoms), and 46%, 37%, 59% (low QoL) for 1st, 2nd and 3rd trimesters, respectively. Gestational age and food insecurity were significant predictors of depressive symptoms, anxiety symptoms and QoL	Gestational age and food insecurity	NR	7 antenatal healthcare facilities	NR
Shuffrey et al. 2022	EPDS	Children born to mothers with both prenatal depression and trait anxiety had higher social-emotional problems (mean difference: 4.66; 95% CI 3.43 to 5.90) compared with children born to mothers with no prenatal depression or trait anxiety, each condition alone, or compared with mothers with depression and state anxiety. Additionally, children born to mothers with prenatal maternal depression and trait anxiety had the greatest reduction in mean cognitive scores on the BSID-III ST (mean difference: − 1.04; 95% CI − 1.99 to –0.08)	Maternal and child demographics, maternal depression and trait anxiety, child socioeconomic and cognitive development	Poor toddler socio-emotional and cognitive development	NR	NR
Mandell et al. 2022	EPDS and STAIS	Diastolic BP was positively associated with both suicidal ideation and depressive symptomatology, even after controlling for demographic variables, gestational age, and intimate partner violence	Blood pressure, suicidal ideation, and depression, demographic variables, gestational age, and intimate partner violence	Antenatal depression and suicidal ideation	12 community health centers	NR

*NR* Not reported

### Study countries and data sources

Half of the eligible studies were conducted in East Africa with Ethiopia providing 58 studies, followed by Kenya (*n* = 13), Tanzania (*n* = 10), Malawi (*n* = 9), Uganda (*n* = 6), Rwanda (*n* = 3), and Sudan (*n* = 2). South Africa provided 22% (*n* = 44) of the included studies with another 22% (*n* = 44) of included studies being conducted in West Africa (Nigeria (*n* = 20) and Ghana (*n* = 16). Others included Egypt (*n* = 8), Zimbabwe (*n* = 6), Cote d’Ivoire (*n* = 5), and the Democratic Republic of Congo (*n* = 2). Cameroon, Mozambique, Eswatini, and Tunisia each contributed one article to the review. The studies were conducted in urban, peri-urban, rural, low-income residential and health facility settings. More information is provided in Supplementary Table 1.

### Study design

Most included studies (*n* = 62) were of mixed-methods study designs. A few studies employed both quantitative and qualitative designs, in most of these the qualitative component was complementary to the quantitative component. Two studies were qualitative exploratory studies (Baumgartner et al. 2014; Mokhele et al. 2019). Thirty-six studies employed prospective/longitudinal cohort/nested designs. Other study designs included randomized controlled trials (*n* = 4), case-controls (*n* = 3), interrupted time series (*n* = 1) and retrospective case note reviews (*n* = 1) Most of the included studies were facility-based. These details are provided in Supplementary Table 1.

### Sampling strategy

Sampling techniques were varied and ranged from such as simple random (*n* = 20) and systematic random sampling (*n* = 39), cluster (*n* = 7), multistage (*n* = 16), stratified (Zotova et al. 2022) and consecutive (*n* = 17) sampling techniques. A few studies applied participant matching (2022; Govender et al. 2020) and randomization (GBD 2019 Mental Disorders Collaborators 2022; Kugbey et al. 2021; Khalifa et al. 2016; Anderson et al. 2021) techniques. Purposive/convenient/quota and census sampling techniques were also used (*n* = 32). Some studies did not report their sampling strategies (*n* = 52) simply because sampling was not applicable in some of them (*n* = 6). Most studies relied on primary data (*n* = 184), while some reanalyzed secondary data (*n* = 32) (Supplementary Table 1).

### Study participants

A pooled sample of 149,647 perinatal and/or women of childbearing age (18 + years) were involved as participants in the 206 included studies. This number included 92,810 prenatal and 40,603 postnatal women. About 1,573 study participants were prenatal or postnatal women, while 2,275 study participants were mothers to under-five children. About 1% (*n* = 1,828) adolescent mothers were included in the studies, and 7.1% (*n* = 10,558) women living with HIV/AIDS. Although almost all studies targeted women of reproductive age group, different studies used different age categories (in years). The mean age of the study participants in the included studies varied from 18.0 to 29.9 years (Supplementary Table 1).

### Measurement and diagnostic tools of perinatal mental disorders

The Edinburgh Postnatal Depression Scale (EPDS) was used by 87 studies to screen for perinatal mental disorders, specifically depression. Other tools used to screen for depression, including perinatal depression included the Beck’s Depression Inventory (BDI) scale, and the Patient Health Questionnaire-9 (PHQ-9). The WHO Self-Reported Questionnaire (WHO-SRQ-20) was used in 8 studies. The WHO Disability Assessment Schedule II (WHO-DAS 2.0), and the World Mental Health (WMH) Survey Initiative Version of the WHO Composite International Diagnostic Interview (CIDI) were also used. To screen for anxiety, the Generalized Anxiety Disorder-7 (GAD-7), the Mini International Neuropsychiatric Interview (MINI) version 5.0, Hospital Anxiety and Depression Scale (HADS), Pregnancy-Related Anxiety Questionnaire-Revised (PRAQ-R), Depression Anxiety and Stress Scale-21 (DASS-21/42), Perinatal Anxiety Screening Scale (PASS), EPDS, BDI, Center for Epidemiological Studies Depression Scale (CES-D), Zung Self-rating Anxiety Scale (ZSAS), Brief Symptom Index (BSI-18), State-Trait Anxiety Inventory (STAI), Spielberger Trait Inventory (STI) and Kessler Psychological Distress Scale (KPDS) were used (Table 1). While many of these assessment tools were validated, it is important to note that a significant number of assessment tools still lack validation in the African context.

## Results

This section presents the epidemiology and diverse determinants influencing maternal mental health problems/disorders, and a thorough exploration of outcomes associated with these problems and/or disorders.

### Epidemiology of maternal mental health disorders

‘Perinatal mental health problems and/or disorders’ captured the common mental health disorders reported during pregnancy, intrapartum and postpartum periods. The burden/magnitude of common perinatal mental disorders (depression, anxiety and generalized anxiety disorders, distress, posttraumatic stress disorder (PTSD) and suicidal ideation/behaviors, as well as psychosis) in the perinatal period (prenatal, intrapartum, and postnatal) is presented. Overall, the prevalence of mental or psychological distress during the perinatal period ranged from 17.0% to 26.0%. Detailed information is presented in Table 1.

### Antepartum depression

The prevalence of antenatal depression ranged from as low as 10.0% to as high as 80.9%. One study conducted in Ethiopia among 646 pregnant reported the prevalence of moderate to severe depression as 11.9% (Jebena et al. 2015). The prevalence of depressive symptoms during pregnancy was found to be 10.8% in a community-based study (Roos et al. 2013).

### Postpartum depression

The prevalence of postpartum depression ranged from 9.2% to 57.0%. while the incidence was 38.8% during the second or third trimester and in the first 4 to 12 weeks following childbirth (Kaiyo-Utete et al. 2020). There was a 21.0% postnatal depression rate for women who had unfavorable pregnancy outcomes such as preterm birth (Gebregziabher et al. 2020). The prevalence of perinatal depression (during both the prenatal and postnatal periods) ranged from 11.0–39.0% (African Union (AU) 2014; Page et al. 2021; Bitew et al. 2017; 2018; Osok et al. 2018; Weobong et al. 2015; Keliyo et al. 2021; Atuhaire et al. 2021; LeMasters et al. 2020; Oladeji et al. 2019). Major perinatal depression ranged from 10.0% to 28.8% (Osok et al. 2018; Rotheram-Fuller et al. 2018; Bitew et al. 2019; Shitu Ayen et al. 2021; Roos et al. 2013), and recurrent depression was estimated at 20.0% in one study (Barsisa et al. 2021). Five distinct trajectory patterns of depressive symptoms in the perinatal period were identified: moderate levels of depressive symptoms during pregnancy but minimal postpartum (3.5%); minimal levels during pregnancy and increasing postpartum (3.7%); unstable levels peaking at 12 months postpartum (6.6%); mild levels with slight decrease postpartum (82.9%); and severe levels during pregnancy and postpartum (3.1%) (Tiki et al. 2020).

Levels of perinatal depression among adolescent mothers ranged between 13.0% (Bitew et al. 2017) and 18.0% (Anato et al. 2020). The burden of perinatal depression among women living with HIV and AIDS was between 8.6% (Gelaye et al. 2016) and 28.7% (Chorwe-Sungani and Chipps 2018), while between 7.2% and 6.4% of women living with HIV were reported to have severe and extremely severe perinatal depression, respectively (Bishaw et al. 2022).

### Anxiety disorders and posttraumatic stress disorders

More than 2 in 5 (43.5%) women of reproductive age reported anxiety in 1 study conducted in Malawi (Bhushan et al. 2022). The prevalence of perinatal anxiety ranged between 31.0% and 44.0% in Sudan, Nigeria and Ethiopia (Rodriguez et al. 2018; Stellenberg and Abrahams 2015; xxxx). Moreover, the prevalence of generalized anxiety disorder (GAD) among perinatal women ranged from 11.0% to 44.0% (Larsen et al. 2022; Weobong et al. 2015; Adeyemo et al. 2020; Oboro et al. 2022; Koen et al. 2016), with 8.0% of cases reporting severe GAD (Koen et al. 2016) and PTSD (Larsen et al. 2022). An estimated 3.3% and 7.7% of perinatal women had severe and extremely severe anxiety, respectively in a study conducted in Congo DR (Bishaw et al. 2022). The prevalence of comorbid anxiety–depression ranged from 10.0% (Adamu and Adinew 2018) to 21.0% (Tsai et al. 2016) in perinatal women (Oladeji et al. 2022; Masiano et al. 2022; Gordon et al. 2021; Weobong et al. 2014; Madeghe et al. 2016).

### Suicide ideation and self-harm behaviors

Estimates of between 13.0 to 42.0% perinatal women reported suicidal ideation/behaviors (González-Guarda and Ortega 2018; 2014; n.d; Rodriguez et al. 2018; Rencken et al. 2022; Oladeji et al. 2022; Sewnet Amare et al. 2022; Woldetsadik et al. 2019; Roos et al. 2013), and about 18.0% in Ghana (Oladeji et al. 2022) and 39.0% in Northern Uganda (Lodebo et al. 2020) reported thinking about self-harm during the perinatal period (Oladeji et al. 2022).

### Psychosis

Only two studies from Ethiopia reported psychosis in the perinatal period. High rates of schizophrenia (48.7%) and mania (15.0%) were reported (Abebe et al. 2019). Between 19.0% and 27.0% of perinatal women were assessed with moderate-to-high risk psychosis in a second study (Belete et al. 2021). The study population was characterized by unplanned pregnancies, a history of suicidal ideation and other common mental disorders, and lack of social support (Abebe et al. 2019; Belete et al. 2021).

## Determinants of perinatal mental health problems and/or disorders

Determinants of perinatal mental disorders included sociodemographic and socioeconomic factors, maternal characteristics and family set-up.

### Sociodemographic/socioeconomic characteristics, household dynamics and social norms

Sociodemographic factors associated with perinatal mental disorders included maternal socioeconomic status (Rurangirwa et al. 2018; Chorwe-Sungani and Chipps 2018; Peltzer et al. 2018; Ndukuba et al. 2015; Baumgartner et al. 2014; xxxx; Abdelghani et al. 2021; Abebe et al. 2022; Koen et al. 2016; Stewart et al. 2019), income and education levels (Mwita et al. 2021; Koen et al. 2016; Duko et al. 2019), employment status (Shitu et al. 2019; January and Chimbari 2018; Wall et al. 2018; Rwakarema et al. 2015; Koen et al. 2016; Duko et al. 2019), place of residence (urban or rural) (Koen et al. 2016), financial/economic situation (Ngene and Moodley 2022; Weobong et al. 2015; Mokwena and Masike 2020; 2017), socioeconomic resources (Garman et al. 2019), the wealth index of individual women (González-Guarda and Ortega 2018) or households (LeMasters et al. 2020; Arach et al. 2020; Alenko et al. 2020; 2014; Shamu et al. 2016), living arrangements/conditions (LeMasters et al. 2020; Weobong et al. 2014) and poverty status (Rotheram-Fuller et al. 2018). In one study, prenatal depression was 4 times higher in pregnant women with little or no income and those working in the private sector compared to housewives and those earning high monthly income (Mossie et al. 2017). Similarly, unemployed women had a 51.0% higher chance of developing postnatal depression than their employed counterparts according to one study in Nigeria (Sulyman et al. 2016). In other words, low socioeconomic position, low education, low income, unemployment, and low household income were linearly associated with depression in mothers (Onah et al. 2017; Barthel et al. 2017; Rurangirwa et al. 2018; MacGinty et al. 2018; Abadiga 2019; Wycliffe et al. 2021; Dlamini et al. 2019; Davies et al. 2016; Garman et al. 2019). Overall, financial support was associated with a decrease in the prevalence of common mental disorders in women (Abrahams et al. 2022). For example, pregnant women engaged in private sector employment reported a lower likelihood of developing depression compared to those who were housewives (Mossie et al. 2017).

Other household dynamics, such as the number of children (Bishaw et al. 2022; Ayele et al. 2021; Tamiru et al. 2022; Saeed and Wemakor 2019) or household size (Belete et al. 2020) were directly associated with perinatal mental disorders. Moreover, social norms such as the unfavorable societal reaction to learning of a pregnancy (Bitew et al. 2017), baby’s sex preference (Shitu et al. 2019; Mokwena and Masike 2020; Necho et al. 2020; Khalifa et al. 2016; Fantahun et al. 2018) and misperception (e.g., witchcraft) (Stewart et al. 2014) were associated with perinatal mental disorders. A not preferred sex of the newborn might double the likelihood of postnatal depression (Shitu et al. 2019).

### Maternal age

In epidemiological models, maternal age was associated with perinatal mental health disorders in several studies (Tilahun et al. 2022; Wong et al. 2017; Osok et al. 2018; Ndukuba et al. 2015; Arach et al. 2020; Mbarak et al. 2019; Duko et al. 2019). Particularly, young women aged 18–23 years were 2–10 times more likely to suffer depressive symptoms compared to their older counterparts (Osok et al. 2018; Mbarak et al. 2019).

### Marital status and spousal support

Perinatal mental disorders were associated with the marital status of women (Arach et al. 2020; Wassif et al. 2019). Unmarried women (Mossie et al. 2017; Onah et al. 2017; Saeed and Wemakor 2019; Wemakor and Mensah 2016; Harrington et al. 2019; Belete and Misgan 2019; Fantahun et al. 2018), those living together without formal commitment (Mutahi et al. 2022), and divorced or widowed women (Belay et al. 2018; Shitu et al. 2019; Lodebo et al. 2020) were more likely to screen for poor mental health, including postnatal depression compared to women who were married or in union (Shitu et al. 2019). Other marital status-related issues, such as age at marriage (Roos et al. 2013), duration of marriage (Boakye-Yiadom et al. 2015), polygamy (Mwita et al. 2021; Dlamini et al. 2019; Kaida et al. 2014; Khalifa et al. 2016; Lillie et al. 2020), poor marital relationship (Wycliffe et al. 2021; Kassaw and Pandey 2020; Naudé et al. 2022), or low marital satisfaction (Rodriguez et al. 2018; Odinka et al. 2018; Abegaz et al. 2022; Akinsulore et al. 2021) were associated with mental health disorders in women. For example, unsatisfactory marital relations or marital conflicts and dissatisfaction were associated with an increase in the likelihood of postnatal depression (Ngocho et al. 2022; Tuthill et al. 2017; Odinka et al. 2018; Oboro et al. 2022).

Poor support from partner/spouse (Ngocho et al. 2022; Umuziga et al. 2022; Rurangirwa et al. 2018; Wake et al. 2022; Kim et al. 2021; Mwita et al. 2021; Gebregziabher et al. 2020; Govender et al. 2020) and the perception that the partner was not emotionally supportive quadrupled antenatal depression symptoms (Chorwe-Sungani and Chipps 2018). For example, the absence of support from a spouse/partner increased the prevalence of postpartum depression by 66.0% in one study (MacGinty et al. 2018), and 82.0% in another (Osok et al. 2018). Relationship problems with a spouse or partner (January and Chimbari 2018; Nydoo et al. 2017), infidelity in marriage (González-Guarda and Ortega 2018; Kakyo et al. 2012; Roos et al. 2013), or social isolation in socially vulnerable women, such as sex workers, were all associated with mental health stressors (Bisetegn et al. 2016). Furthermore, stigma African Union (AU) 2014; Rodriguez et al. 2018; Brittain et al. 2017; Khan et al. 2020; LeMasters et al. 2020; Bisetegn et al. 2016; Yator et al. 2016; Garman et al. 2019), loneliness, self-blame, and guilt (Ogueji 2021), and poor communication in marriage (Ahmed et al. 2021; Wycliffe et al. 2021) were associated with a range of perinatal mental health problems. On the contrary, partner/husband support (Shitu et al. 2019; Abrahams and Lund 2022; Adamu and Adinew 2018; Dadi et al. 2021; Okunola et al. 2022; Nwafor et al. 2021; Bhushan et al. 2022; Mokwena and Mbatha 2021; Redinger et al. 2021; Khan et al. 2020; Akinsulore et al. 2021; Mokwena and Masike 2020; Govender et al. 2020; Mbatha et al. 2020; Saeed and Wemakor 2019; Woldetsadik et al. 2019; Guo et al. 2014) was a protective factor against common perinatal mental disorders.

### Individual and household food and water insecurity

Experiencing food insecurity or hunger, whether at the individual or household level, including improper feeding practices, insufficient food, poor nutrition, or water insecurity was associated with a variety of perinatal mental disorders, including antenatal depressive symptoms (Gureje et al. 2022; Onah et al. 2017; Pingo et al. 2017; 2018; Jebena et al. 2015; Adebowale and James 2020; Tesfaye and Agenagnew 2021; Dadi et al. 2021; Tamiru et al. 2022; Gordon et al. 2021; Ngocho et al. 2019; Weobong et al. 2014; 2015; Shamu et al. 2016; Tsai et al. 2016; Boakye-Yiadom et al. 2015; Lillie et al. 2020; Yator et al. 2016).

### Parenting and parental context

The burden of motherhood (Kaiyo-Utete et al. 2020), or childcare (Tefera et al. 2015), parental distress, and dysfunctional parent–child interactions (Guo et al. 2014) were also linked to perinatal mental health disorders. Perceived difficulty of raising children predisposed women to perinatal depression by up to 57.0% in a study conducted in Kenya (Tefera et al. 2015). Dysfunctional parent–child interactions could also be a consequence of poor perinatal mental health (Guo et al. 2014). Likewise, for younger women, not living with their parents during pregnancy (Mbawa et al. 2018) and a lack of male/partner involvement during pregnancy (Rodriguez et al. 2018) were associated with perinatal mental disorders, whereas family visits during the perinatal period, the presence of emotional assistance and a woman’s autonomy were found to protect women against perinatal mental disorders (Adjorlolo et al. 2022; Abebe et al. 2019).

### Intimate partner and interpersonal violence

The typologies of intimate partner violence included physical (2014; Ngocho et al. 2022; Larsen et al. 2022; Ghoneim et al. 2021), sexual (xxxx; Larsen et al. 2022; Redinger et al. 2021), psychological/verbal (Larsen et al. 2022; Barthel et al. 2017; Redinger et al. 2021) or parental (Bindt et al. 2013) violence and their degree of association with perinatal mental health disorders varied (Bitew et al. 2019; Gold et al. 2013; Tsai et al. 2016). Intimate or interpersonal partner violence or abuse was significantly associated with a broad spectrum of perinatal mental disorders in women of all age groups and socioeconomic classifications. Intimate partner and interpersonal violence exacerbated mental ill-health among women reporting major depressive episodes or psychiatric illnesses, leading to the doubling of episodes of suicidal thoughts (Yotebieng et al. 2017). In addition, compared to other women, those who had experienced domestic violence reported 3 times as many cases of postpartum depression (Yotebieng et al. 2017). Indeed, having experienced violence throughout one's life increases the likelihood of developing depressive symptoms in pregnant women (WHO 2004; Herrman et al. 2006; WHO 2019). A history of rape was also associated with postpartum depression (Roos et al. 2013).

### Availability of social support

A lack of or poor social support, internalized shame and feelings of social rejection were particularly prevalent among women with mental ill-health (Adeoye et al. 2022; Yotebieng et al. 2017; Gebremichael et al. 2017; Belay et al. 2018; Rotheram-Fuller et al. 2018; Guo et al. 2013). An epidemiological study reported that a 1-unit increase in social rejection increased the depression score by 0.7 units in young pregnant women in Ethiopia (Tilahun et al. 2022). Similarly, poor, or low social support is associated with postpartum depression by up to 4 times (Yotebieng et al. 2017; Belay et al. 2018). Perceived availability of a support system (WHO 2019; Ngocho et al. 2019; Kaaya et al. 2016; Koen et al. 2016), including partner (WHO 2019) or social support (McNab et al. 2022; GBD 2019 Mental Disorders Collaborators 2022; González-Guarda and Ortega 2018; Umuziga et al. 2022; Tefera et al. 2015; Stewart et al. 2015; Stellenberg and Abrahams 2015; Abate et al. 2021; Ade-Ojo et al. 2022; Adebowale and James 2020; Abdelhai and Mosleh 2015; Acheampong et al. 2021; Abadiga 2019; Abrahams and Lund 2022; Abegaz et al. 2022; Okafor et al. 2021; Ogueji 2021; Ogueji 2021; Malaju et al. 2022; Ayele et al. 2021; Kim et al. 2021; Oboro et al. 2022; Khan et al. 2020; Gordon et al. 2021; Phukuta and Omole 2020; Dadi et al. 2020; Bitew et al. 2019; Shitu Ayen et al. 2021; Mbatha et al. 2020; Modjadji and Mokwena 2020; Mnisi et al. 2020; Mbarak et al. 2019; Kaaya et al. 2016; 2014; 2017; Kakyo et al. 2012; Shamu et al. 2016; Koen et al. 2021; xxxx; Guo et al. 2014; Bindt et al. 2013; Boakye-Yiadom et al. 2015; Roos et al. 2013; Bekele et al. 2017; Lillie et al. 2020; Koen et al. 2016) decreased the likelihood of depressive symptoms, stress and GAD in perinatal women. Social support factors that protected perinatal women from a range of mental health problems included psychosocial support (Knettel et al. 2020) and access to psychosocial support information (Chorwe-Sungani and Chipps 2018); family/relative support (Abegaz et al. 2022; Wycliffe et al. 2021; Mokwena and Masike 2020; Belete et al. 2019; Kakyo et al. 2012; Bekele et al. 2017); social support from female friends (Harrington et al. 2018); community support (Gold et al. 2013); social participation (Lillie et al. 2020); and presence of a self-help groups as a source of hope (Chingono et al. 2022).

### Lifestyle disorders, substance and harmful alcohol use

Current or previous substance use (González-Guarda and Ortega 2018; Yotebieng et al. 2017; Abebe et al. 2022; Adebowale and James 2020; Adamu and Adinew 2018; Dadi et al. 2021; Wycliffe et al. 2021; Malaju et al. 2022; Ayele et al. 2021; Nwafor et al. 2021; Kim et al. 2021; Ghoneim et al. 2021; Khan et al. 2020; Knettel et al. 2020; Kassaw and Pandey 2020; Govender et al. 2020; Dadi et al. 2020; Shitu Ayen et al. 2021; Mbarak et al. 2019; Saeed and Wemakor 2019; Davies et al. 2016; Stewart et al. 2014; Manikkam and Burns 2012; Khalifa et al. 2016; Kakyo et al. 2012; Koen et al. 2021; Boakye-Yiadom et al. 2015), abuse (Yator et al. 2016), partner substance use (Bindt et al. 2013; Roos et al. 2013) or personality disorders (Rwakarema et al. 2015; Kaaya et al. 2016) were all linked with a range of perinatal mental disorders. Notably, compared to non-users, women who used substances were 5 times more likely to suffer postpartum depression (Toru et al. 2018). Alcohol use (Kaiyo-Utete et al. 2020), problematic alcohol use (Onah et al. 2017) or a positive fasting alcohol level test (an indication of alcohol use) (Abdelhai and Mosleh 2015), especially during pregnancy (Azale et al. 2016) were associated with perinatal mental disorders. In addition, women who smoked cigarettes or used tobacco (Tefera et al. 2015; Barsisa et al. 2021) (or whose spouses did) were more likely to report mental health problems (Page et al. 2021; Osok et al. 2018). Lifestyles related to eating disorders, like binge eating, were also associated with antenatal depression as a risk factor or a consequence (Adeoye et al. 2022).

### Childhood trauma and stressful life events

Experiencing trauma in general (Abebe et al. 2022), and history of childhood trauma and adverse childhood events (MacGinty et al. 2020; Koen et al. 2016) such as sexual abuse (Belete et al. 2020; Tsai et al. 2016) and associated external behavior problems (xxxx) were associated with perinatal mental health problems. Likewise, perceived stress (Adeoye et al. 2022; Wall et al. 2018), exposure to negative/stressful life events/stressors during pregnancy (Gebremichael et al. 2017; Guo et al. 2013; Tefera et al. 2015; Stewart et al. 2015; Abrahams and Lund 2022; 2022; Masiano et al. 2022; Kaiyo-Utete et al. 2020; Tiki et al. 2020; Phukuta and Omole 2020; Mokwena and Masike 2020; Mbarak et al. 2019; Alenko et al. 2020; Davies et al. 2016; Stewart et al. 2014; Wemakor and Mensah 2016; 2017; Ayele et al. 2016; Duko et al. 2019; Belete and Misgan 2019), such as pregnancy losses, violence, life-threatening events (GBD 2019 Mental Disorders Collaborators 2022; Natamba et al. 2017; Adebowale and James 2020; Abdelhai and Mosleh 2015; Malaju et al. 2022; Ngocho et al. 2019) and family stress (Barthel et al. 2017) were associated with depression and anxiety. Experiencing stressful events such as major stresses, changes, or losses during pregnancy increased the likelihood of antenatal depression by more than three times (Gebremichael et al. 2017; Natamba et al. 2017). Family stress was also linked to a twofold increase in depressive and anxiety symptoms among low-income women (Barthel et al. 2017). In contrast, the ability to cope with stress decreased the likelihood of antenatal depression (Khan et al. 2020). These associations were corroborated by findings from a qualitative study conducted in South Africa (Davies et al. 2016).

## Pregnancy-related determinants

### Unintended or unwanted pregnancy

Unintended/unwanted pregnancy, leading to an “unwelcomed” baby (Umuziga et al. 2022; Larsen et al. 2022; Brittain et al. 2017; Ahmed et al. 2021; Keliyo et al. 2021; Umuziga et al. 2020; Alenko et al. 2020; Davies et al. 2016; Garman et al. 2019; Harrington et al. 2019; Osborn et al. 2022), repeated pregnancy (Govender et al. 2020) and a lack of information about contraceptive options (Bitew et al. 2017) were associated with perinatal mental health disorders in women. When compared to planned births, postpartum depression was 2–3 times more likely to occur in women who reported unwanted or unplanned pregnancies (Brittain et al. 2017). Conversely, the risk of depression in women who reported planned pregnancy or ready for the pregnancy was low (Adeoye et al. 2022; 2021; Oboro et al. 2022; Masiano et al. 2022; Ghoneim et al. 2021; Belete et al. 2020; Umuziga et al. 2020; Govender et al. 2020; Dadi et al. 2020; Shitu Ayen et al. 2021; Gold et al. 2013; Mochache et al. 2018; Bindt et al. 2013; Ayele et al. 2016; Bekele et al. 2017; Duko et al. 2019). These associations were complemented by a qualitative finding (Jidong et al. 2021).

### Obstetric-related factors and complications

Maternal (Baumgartner et al. 2014) and pregnancy/obstetrics-related factors or characteristics (Pan et al. 2024; Ade-Ojo et al. 2022; Abdelghani et al. 2021) such as gestational age (Tamiru et al. 2022; Oladeji et al. 2019; Bindt et al. 2013; Boakye-Yiadom et al. 2015; Wassif et al. 2019; Pobee et al. 2022; Mandell et al. 2022; Osborn et al. 2022), e.g., ranging from 16–39 weeks (Anderson et al. 2021), were also associated with perinatal mental disorders. In epidemiological analyses, parity (Umuziga et al. 2022; Gebremichael et al. 2017; Sulyman et al. 2016; Tamiru et al. 2022; Kakyo et al. 2012; Boakye-Yiadom et al. 2015), birth order (Shifa et al. 2018) and low birthweight (Tomita et al. 2015) were also factors associated with mother’s mental health status. Furthermore, maternal weight, body mass index (BMI) and body image (Kugbey et al. 2021; 2014; Kariuki et al. 2022), especially at postpartum, were associated with postpartum depression (Kariuki et al. 2022).

Pregnancy-related complications, throughout the peripartum period, previous pregnancy complications (Ayele et al. 2021; Mokwena and Mbatha 2021; Anbesaw et al. 2021; Beyene et al. 2021; MacGinty et al. 2020; Knettel et al. 2020; Akinsulore et al. 2021; Belete et al. 2020; Kassaw and Pandey 2020; 2011; Bitew et al. 2019; Mbatha et al. 2020; Pellowski et al. 2019; Dlamini et al. 2019; Belete et al. 2019; Manikkam and Burns 2012; Bindt et al. 2012; Gold et al. 2013; Mochache et al. 2018; Guo et al. 2014; Okronipa et al. 2012) or “fear” of pregnancy or pregnancy-related complications were associated with a range of perinatal mental disorders (Dadi et al. 2020; Beyene et al. 2021; Malaju et al. 2022). Danger signs in pregnancy (Bante et al. 2021) and preterm delivery (Oboro et al. 2022; Weobong et al. 2014; Mochache et al. 2018), as well as history of adverse perinatal outcomes (Ayele et al. 2021; Woldetsadik et al. 2019), such as history of abortion or miscarriage (Tesfaye and Agenagnew 2021; Beyene et al. 2021; Tamiru et al. 2022; Belete et al. 2021; Anbesaw et al. 2021; Oboro et al. 2022; Akinsulore et al. 2021; Gebregziabher et al. 2020; Tiki et al. 2020; Phukuta and Omole 2020; Belete et al. 2020; Govender et al. 2020; Woldetsadik et al. 2019; Kakyo et al. 2012; Guo et al. 2014; Roos et al. 2013; Ayele et al. 2016; Bekele et al. 2017; Bisetegn et al. 2016; Garman et al. 2019; Nyamukoho et al. 2019; Duko et al. 2019; Woldetensay et al. 2021) were all risk factors for perinatal mental disorders. The effect of these complications or adverse outcomes on perinatal mental health disorder could range from 2–5 times higher when compared to mothers with normal pregnancies or deliveries (Weobong et al. 2014; Nyamukoho et al. 2019).

## Child-related determinants

### Child health and development problems

Child health issues including birthweight and newborn complications, birth defects, illnesses/hospitalization, nutritional status, sleep problems, sub-optimal child socioemotional and cognitive development (Rodriguez et al. 2018; Rodriguez et al. 2018; Adjorlolo et al. 2022; Abebe et al. 2019; Abrahams and Lund 2022; Anbesaw et al. 2021; Gordon et al. 2021; Shitu Ayen et al. 2021; Mbatha et al. 2020; Kaaya et al. 2016; Gold et al. 2013; Fantahun et al. 2018; Shuffrey et al. 2022) were all associated with maternal mental health problems. Evidence shows that low birthweight (Tomita et al. 2015), infant illness (Shitu Ayen et al. 2021) and sleep problems (Adjorlolo et al. 2022; Abrahams and Lund 2022; Anbesaw et al. 2021) were associated with a high likelihood of postnatal depression by approximately three-, two- and fourfold, respectively.

## Child death

History of stillbirth (Beyene et al. 2021; Woldetsadik et al. 2019; Gold et al. 2013; Bisetegn et al. 2016), neonatal/infant, and child death (Shifa et al. 2018; Tefera et al. 2015; Wake et al. 2022; Woldetsadik et al. 2019; Harrington et al. 2019; Fantahun et al. 2018) were associated with various perinatal mental disorders. For instance, women who lost a child were twice more likely to experience mental distress than mothers who did not lose a child (Shifa et al. 2018).

## Maternal health-related determinants

### Medical conditions

The presence of self-reported or diagnosed physical illnesses, both acute and chronic, such as hypertension and syphilis, or hospitalization were associated with a range of perinatal mental disorders (January and Chimbari 2018; Tefera et al. 2015; Stewart et al. 2015; Mohammed et al. 2014; Adebowale and James 2020; Ahmed et al. 2021; Adamu and Adinew 2018; Okafor et al. 2021; Bante et al. 2021; Wycliffe et al. 2021; Beketie et al. 2021; Rencken et al. 2022; Belete et al. 2021; Keliyo et al. 2021; Anbesaw et al. 2021; Kaiyo-Utete et al. 2020; Belete et al. 2020; Modjadji and Mokwena 2020; Dlamini et al. 2019; Belete et al. 2019; Alenko et al. 2020; Manikkam and Burns 2012; 2014; xxxx; Okronipa et al. 2012; Ayele et al. 2016; Lillie et al. 2020; Koen et al. 2016; Stewart et al. 2019). Women who reported physical illnesses were twice as likely to experience postpartum depression compared to their healthier counterparts (Sulyman et al. 2016). Concerns about COVID-19 also elevated the risk of prenatal mental illnesses (Abate et al. 2021; Ade-Ojo et al. 2022; Abrahams et al. 2022; Abrahams and Lund 2022; Bishaw et al. 2022; Nwafor et al. 2021; Sewnet Amare et al. 2022; Kassaw and Pandey 2020). In contrast, reporting no medical problems and attending antenatal care (ANC) reduced women's mental distress by between 57.0% and 64.0% (Ayele et al. 2016). Physical, psychological, social and overall quality of life were associated with depression in women, including among adolescent expectant women (Oladeji et al. 2019; Soyemi et al. 2022).

### HIV and AIDS

HIV/AIDS and the dynamics related to living with the disease were associated with perinatal mental health disorders (Rotheram-Fuller et al. 2018; Wycliffe et al. 2021; Belete et al. 2021; Keliyo et al. 2021; Sewnet Amare et al. 2022; Tiki et al. 2020; Belete et al. 2020; Mbatha et al. 2020; Modjadji and Mokwena 2020; Saeed and Wemakor 2019; Woldetsadik et al. 2019; Alenko et al. 2020; Necho et al. 2020; Wemakor and Mensah 2016; Kakyo et al. 2012; Anderson et al. 2021; xxxx; Boakye-Yiadom et al. 2015; Azale et al. 2016; Garman et al. 2019). Being diagnosed with HIV/AIDS (Gebremichael et al. 2017) or to be HIV seropositive (Onah et al. 2017; Shitu Ayen et al. 2021), partner's positive HIV status (Abebe et al. 2019; Barsisa et al. 2021; Gordon et al. 2021; Pellowski et al. 2019), disclosure of positive HIV status to partner (2014; Sewnet Amare et al. 2022; Madeghe et al. 2016), HIV-related stigma (Gelaye et al. 2016; Tilahun et al. 2022; Abate et al. 2021; Sewnet Amare et al. 2022; Gordon et al. 2021), non-adherence to antiretroviral treatment (Barthel et al. 2017; Wall et al. 2018; Abate et al. 2021; Necho et al. 2020), non-condom use at last sex (Wall et al. 2018), viral load/CD4 counts and WHO-HIV clinical stage (Tomita et al. 2015; Abebe et al. 2019; Tamiru et al. 2022; Goweda and Metwally 2020; Saeed and Wemakor 2019; Stewart et al. 2014; Madeghe et al. 2016), and HIV status of the newborn (Kakyo et al. 2012) were associated with a range of perinatal mental disorders. Poor adherence to antiretroviral therapy increased antenatal depression by 49.0% (Wall et al. 2018). On the contrary, access to HIV support information (Abate et al. 2021), HIV knowledge (Mokhele et al. 2019) and adherence to ART (Peltzer et al. 2018) were protective against perinatal mental health disorders.

## Pre-existing or previous mental illnesses

### Mental illnesses

Self and/or family history of mental disorders was strongly associated with perinatal mental disorders (Page et al. 2021; Osok et al. 2018; Shitu et al. 2019; Peltzer et al. 2018; Guo et al. 2013; Tefera et al. 2015; Stewart et al. 2015; Stellenberg and Abrahams 2015; xxxx; Ade-Ojo et al. 2022; Agbaje et al. 2019; Parcesepe et al. 2021; Bante et al. 2021; Wycliffe et al. 2021; Rencken et al. 2022; Nwafor et al. 2021; Mokwena and Mbatha 2021; Oboro et al. 2022; Umuziga et al. 2020; 2017; Madeghe et al. 2016). There were strong and overwhelmingly significant associations between pre-existing, ‘comorbid’ or recurrent depression and perinatal mental disorders (2014; Page et al. 2021; Ngene and Moodley 2021; Bitew et al. 2017; Brittain et al. 2017; Peltzer et al. 2018; Rwakarema et al. 2015; Baumgartner et al. 2016; Mohammed et al. 2014; Stellenberg and Abrahams 2015; Adamu and Adinew 2018; Ayele et al. 2021; Tamiru et al. 2022; Oladeji et al. 2022; Anbesaw et al. 2021; Kim et al. 2021; Bitew et al. 2019; Mnisi et al. 2020; 2017; Tsai et al. 2016; xxxx; Boakye-Yiadom et al. 2015; Okronipa et al. 2012; Madeghe et al. 2016; Roos et al. 2013; Wassif et al. 2019; Stewart et al. 2019; Garman et al. 2019; Belay et al. 2019), including major depressive episodes (Mutahi et al. 2022). There was between two- to sevenfold increased association in postpartum depression for women with a history of depression (Gebremichael et al. 2017; Rogathi et al. 2017). Moreover, self/family history of anxiety symptoms was associated with perinatal mental disorders (Ngene and Moodley 2021; Rurangirwa et al. 2018; Peltzer et al. 2018; Rwakarema et al. 2015; Adamu and Adinew 2018; Rencken et al. 2022; Oladeji et al. 2022; Sewnet Amare et al. 2022; Madeghe et al. 2016; Lillie et al. 2020; Wassif et al. 2019; Garman et al. 2019), including postnatal depressive symptoms (Rurangirwa et al. 2018). Suicidal ideation and attempts (Rodriguez et al. 2018; Onah et al. 2017; Shifa et al. 2018; Adjorlolo et al. 2022; Rencken et al. 2022; Belete et al. 2021; Anbesaw et al. 2021; Knettel et al. 2020; Goweda and Metwally 2020) were associated with perinatal mental health disorders. Self-harm tendencies (Wong et al. 2017; Rencken et al. 2022) were associated with an increased likelihood of depression in women by up to 3 times (Wong et al. 2017). It is important to note that in a reverse association, self-harm could also be a consequence of poor mental health in perinatal women (Kassaw and Pandey 2020; Ngocho et al. 2019; Oladeji et al. 2019; 2014; Stewart et al. 2019).

## Outcomes related to perinatal mental disorders

### Pregnancy-related outcomes of perinatal mental disorders

Several pregnancy-related outcomes (as listed in Table 1), including low ANC attendance and poor postnatal care attendance (Agbaje et al. 2019), were reported in women who experience perinatal mental disorders. For example, the increased tendency to have more non-scheduled ANC visits, as did the number of emergency healthcare visits to both traditional providers and biomedical providers for pregnancy-related emergencies (Bitew et al. 2016), pre-eclampsia, caesarean section, and episiotomy (Acheampong et al. 2021) were some pregnancy-related experiences for mentally unwell perinatal women.

### Short-term outcomes of perinatal mental disorders

Preterm delivery (Oboro et al. 2022; Weobong et al. 2014; Mochache et al. 2018), low birth weight (Tomita et al. 2015; Oladeji et al. 2022; Wemakor and Mensah 2016; Weobong et al. 2014), premature births and admission to neonatal intensive care unit (Mostafa et al. 2021; Mnisi et al. 2020), smaller head circumference (MacGinty et al. 2020), neonatal/infant complications/morbidities (e.g., the difficulty of breathing or fast breathing and convulsions or spasms, acute infections, diarrhea) (Bitew et al. 2017; Desalegn et al. 2022; Dadi et al. 2021), non-exclusive breastfeeding (Madeghe et al. 2016), malnutrition (Dadi et al. 2021; Kaaya et al. 2016) and neonatal/infant mortality (Mostafa et al. 2021; Weobong et al. 2014) were reported more by women who experienced mental health disorders than by their healthier counterparts. For example, the likelihood of low birth weight in women who experienced antenatal depression was 84% higher as compared to mentally well mothers (Tomita et al. 2015). Furthermore, 1 in 4 women with depressive symptoms gave birth prematurely, and the probability of doing so was 3.8 times higher in this group (Mostafa et al. 2021). Likewise, exclusive breastfeeding decreased by 28.0% in women who experienced perinatal depression (Tuthill et al. 2017). Moreover, infant mortality was twice as high for children born to women who experienced perinatal mental disorders (Weobong et al. 2014).

Complications during pregnancy (e.g., edema, blurred vision, severe abdominal pain, abnormal vaginal discharge, burning sensation while urinating, and severe headache), labor complications (e.g., severe headache convulsion, hemorrhage, unconsciousness, fever, premature rupture of membranes, prolonged labor and retained placenta) and postpartum complications (e.g., convulsion, hemorrhage, unconsciousness and fever) were reported by those perinatal women who reported mental disorders (Bitew et al. 2017). For instance, women with antenatal depressive symptoms were twice as likely to report complications during pregnancy, labor, and the postpartum period (Bitew et al. 2017; Shitu Ayen et al. 2021). Similarly, depressed women had an increased risk of pre-eclampsia, cesarean section, and episiotomy (Acheampong et al. 2021).

### Long-term outcomes of perinatal mental disorders

Infant cognitive, fine/gross motor developmental and socioemotional delays (Rodriguez et al. 2018; Koen et al. 2021; Shuffrey et al. 2022), compromised children’s physical growth (e.g., weight and height) (Rotheram-Fuller et al. 2018), stunted growth (Wemakor and Mensah 2016) and behavior problems (e.g., attention deficit, aggression, internalizing or externalizing symptoms) (Rotheram-Fuller et al. 2018), as well as lasting medical symptoms (e.g., presence of recurrent wheeze (MacGinty et al. 2018) were reported problems by mother who experienced perinatal mental disorders.

## Sources of healthcare access

The review also sought to synthesize evidence on where women experiencing perinatal mental health problems/disorders sought care and support. Care for perinatal mental disorders was reportedly accessed in formal healthcare (government/public facilities), including community health centers, or primary maternity care clinics and hospitals (district/tertiary) (WHO 2019; McNab et al. 2022; González-Guarda and Ortega 2018; Mutahi et al. 2022; Pan et al. 2024; Gelaye et al. 2016; 2015; Grace and Sansom 2003; 2014; Baron et al. 2016; xxxx; Umuziga et al. 2022; Gureje et al. 2022; Ngene and Moodley 2021; Larsen et al. 2022; Bitew et al. 2017; Rodriguez et al. 2018; Khalifa et al. 2018; Wong et al. 2017; Pingo et al. 2017; Brittain et al. 2017; Mbawa et al. 2018; Rodriguez et al. 2018; 2018; Toru et al. 2018; Anokye et al. 2018; January and Chimbari 2018; Wall et al. 2018; Chorwe-Sungani and Chipps 2018; Peltzer et al. 2018; Rwakarema et al. 2015; Tomita et al. 2015; Jebena et al. 2015; Ndukuba et al. 2015; Weobong et al. 2015; Baumgartner et al. 2016; Bitew et al. 2016; Guo et al. 2013; Baumgartner et al. 2014; Tefera et al. 2015; Mohammed et al. 2014; Stellenberg and Abrahams 2015; Sulyman et al. 2016; xxxx; Abate et al. 2021; Ade-Ojo et al. 2022; Abdelghani et al. 2021; Abebe et al. 2022; Abrahams et al. 2022; Adjorlolo et al. 2022; Ahmed et al. 2021; Adeyemo et al. 2020; Abebe et al. 2019; Abadiga 2019; Abrahams and Lund 2022; Abegaz et al. 2022; Adamu and Adinew 2018; Agbaje et al. 2019; Desalegn et al. 2022; Tesfaye and Agenagnew 2021; Mostafa et al. 2021; Okafor et al. 2021; Bante et al. 2021; Beyene et al. 2021; Dadi et al. 2021; Ogueji 2021; Wycliffe et al. 2021; Bishaw et al. 2022; Zotova et al. 2022; Malaju et al. 2022; Beketie et al. 2021; Wake et al. 2022; Borie et al. 2022; Rencken et al. 2022; Okunola et al. 2022; Ayele et al. 2021; Tamiru et al. 2022; 2022; Oladeji et al. 2022; Kugbey et al. 2021; Belete et al. 2021; Keliyo et al. 2021; Nwafor et al. 2021; Sewnet Amare et al. 2022; Atuhaire et al. 2021; Bhushan et al. 2022; Mokwena and Mbatha 2021; Kim et al. 2021; 2021; Oboro et al. 2022; Jones et al. 2021; Masiano et al. 2022; Redinger et al. 2021; MacGinty et al. 2020; Knettel et al. 2020; Gordon et al. 2021; Anato et al. 2020; Tiki et al. 2020; Phukuta and Omole 2020; Belete et al. 2020; Mokwena and Masike 2020; Kassaw and Pandey 2020; LeMasters et al. 2020; Umuziga et al. 2020; Dadi et al. 2020; Modjadji and Mokwena 2020; Mbarak et al. 2019; Goweda and Metwally 2020; Saeed and Wemakor 2019; Dlamini et al. 2019; Belete et al. 2019; Woldetsadik et al. 2019; Necho et al. 2020; Mochache et al. 2018; Khalifa et al. 2016; Kakyo et al. 2012; Tsai et al. 2016; Anderson et al. 2021; xxxx; Guo et al. 2014; Okronipa et al. 2012; Bekele et al. 2017; Koen et al. 2016; Yator et al. 2016; Azale et al. 2016).

A paltry 12.7% of women had visited any health provider since the onset of their symptoms (postnatal depression), while only 4.2% of women with severe postnatal depression symptoms received mental healthcare. Factors such as urban residency, high levels of social support, perceived physical causes, perceived severity, perceived need for treatment, high scores for mental health disorders, and impairment were significantly related to seeking out health services. A study reported that 7 out of 10 women with high levels of postnatal depression symptoms perceived a need for treatment (Azale et al. 2016). None of the eligible studies reported on the perception of healthcare quality.

## Discussion

This review aims to provide an overview of the evidence on maternal mental health, focusing specifically on the epidemiology of perinatal mental health problems/disorders in the African region. While this systematic review identified over 200 eligible studies, there remains a notable research gap specifically addressing the unique challenges and contexts of low- and middle-income countries, particularly in Africa. Additionally, there is a significant variation in the prevalence of perinatal mental health problems in different settings due to differences in definitions, measurements, and interpretations of the findings. The African region presents a complex and diverse landscape marked by significant heterogeneity in participant demographics and various factors associated with perinatal mental health. Moreover, countries in the region report different aspects for maternal and child health outcomes, as well as varying levels of availability and accessibility of mental health services. This situation calls for conducting empirical research that highlights the depth and breadth of the perinatal mental health problem and urges collective actions by all stakeholders. The analysis identifies evidence gaps to guide research projects, advocacy efforts, and policies pertinent to regional contexts.

There are four main takeaways from this current review. First, our analysis revealed that published evidence on perinatal mental health comes mostly from Eastern and South Africa (ESA), and a few from West and Central Africa (WCA). This means that the current evidence in the African region is not truly representative of the region. The implication is that a lot more research needs to be undertaken to understand the prevalence and impact of common perinatal mental health disorders in other regions, especially where maternal and infant mortality rates are arguably higher compared to the ESA region. Current evidence suggests that neglect of maternal mental health may lead to the global failure to reach the SDGs targets of reducing maternal and child mortality (Beyene et al. 2021; Kim et al. 2021). It is therefore important to clearly understand the burden of maternal and child mortality attributable to perinatal mental health disorders in the African region. There is a lack of evidence on psychosis during the perinatal period in the region. However, based on two studies included in this review, it was found that more than 1 in 5 perinatal women are at moderate-to-high risk of experiencing psychosis (Abebe et al. 2019; Belete et al. 2021). These figures significantly surpass the global averages, particularly for postpartum psychosis (Miranda et al. 2005). The specific occurrences of schizophrenia and mania (Abebe et al. 2019) offer insights into the diverse and severe spectrum of psychotic conditions in the African population, highlighting the urgent need to address this understudied facet of maternal mental health. The scarcity of data emphasizes the critical necessity for more extensive research efforts and targeted interventions to unravel the nuanced complexities surrounding psychosis in the perinatal context.

Secondly, this review revealed that socioeconomic disadvantages and other environmental factors, as well as toxic family environment (e.g., intimate partner violence), contributes to common maternal mental ill-health. There are, however, several important questions regarding the associations between perinatal mental ill-health and other outcomes, including whether it is a cause or consequence. For example, it is generally recognized that poor mental health can be both a contributing factor and a consequence of other issues such as substance use or misuse, suicidal thoughts, and experiences of trauma or violence. It is imperative to understand that these relationships are complex and bidirectional, and do not imply that women are responsible for the violence inflicted upon them.

Thirdly, there is inadequate evidence on how the timing, severity, and duration of exposure to the factors listed above affect perinatal mental disorders. Different types of factors may have different mental health consequences. Moreover, previous studies have tended to focus on a single maternal mental illness outcome, for example, depression. The significance of poverty, for example, as a *cause* of disease is a function of the broad range of conditions to which it is related. Moreover, these risk factors vary greatly and there is little known about the various trajectories (Nyamukoho et al. 2019) and the causal pathway and of these risk factors over the life course of reproductive age, that contribute to the rate of maternal mental health disorders. For example, the findings point to the fact that depression can have diverse trajectories from antepartum to the postpartum period (Redinger et al. 2021; Bindt et al. 2013), including chronic-low (71.1%), late-postpartum (10.1%), early postpartum (14.4%), and chronic-high (4.5%) (Tsai et al. 2016), and the patterns of depressive symptoms vary over time. That is, a chronic-low pattern represents stable, consistently low depressive symptoms, while late-postpartum refers to symptoms emerging later in the postpartum period; moreover, early postpartum depressive symptom trajectory denotes symptoms shortly after childbirth, and a chronic-high trajectory reflects enduring, consistently high depressive symptoms (Larsen et al. 2022; Barthel et al. 2017; Malaju et al. 2022; Garman et al. 2019), attributing to different determinants (Malaju et al. 2022; Pellowski et al. 2019; Garman et al. 2019). We thus strongly recommend large-scale studies that collect new data from generalizable population, to delve into the detrimental health, developmental and social effects of common perinatal mental health disorders across the continuum of the perinatal period.

Fourthly, evidence on the appropriateness and validity of screening and diagnostic (measurement) tools is still generally weak in the region. Many studies conducted in the African region have used the EPDS to screen for antepartum and postpartum depression symptoms. While the tool has been validated in various contexts with high levels of sensitivity and specificity, a recent systematic review indicates marked heterogeneity between studies due to differences in methodology and language rendering comparability difficult (Gibson et al. 2009). The systematic review also indicates that the EPDS may not be an equally valid screening tool across all settings and contexts, unless carefully validated and adapted (Gibson et al. 2009). As suggested in evidence, culture has an impact on the experience and expression of mental illness as well as the course and outcome of disorders (Kumar et al. 2015). Hence, semantic and linguistic equivalence are important in developing appropriate screening tools for mental health assessment (Kumar et al. 2015). Another screening tool that has been used to assess major depressive disorder, suicidality, alcohol abuse, generalized anxiety disorder and PTSD is the MINI (Stellenberg and Abrahams 2015). The MINI Plus is a modular DSM-IV-based structured psychiatric tool which has been used widely and has demonstrated cross-cultural validity in diverse settings. Both the validity (i.e. the clinical calibration) and the test–retest reliability of the EPDS and the MINI Plus need further analysis in the African context. Validation exercises of the 10-item CES-D-10 have been undertaken in two studies conducted in South African populations (Gibson et al. 2009). The 10-item Centre for CES-D is a valid, reliable screening tool for South African populations, in particular Zulu, Xhosa and colored Afrikaans populations, but also provided the specific cut-off points that generated the most balanced predictive value (Baron et al. 2017). The PHQ-9 is also frequently used tool to screen for depression (Wong et al. 2017; Wake et al. 2022; Sewnet Amare et al. 2022). More studies are needed in different African countries to develop locally relevant screening tools, or to design short, validated tools that can identify multiple symptoms for common mental disorders (including depression, anxiety, and suicidality) and integrated into the health systems (both community or low primary care levels) or national surveys. This is particularly these in the context of psychosis, highlighting the necessity for screening tools that effectively capture cultural sensitivities and nuances.

### Strengths and limitations

This systematic review has taken a comprehensive approach in synthesizing existing evidence on many aspects of perinatal mental disorders in Africa. We examined various peer-reviewed and grey literature conducted in Africa, covering the entire spectrum of women of reproductive age. We used standard guidelines to assess the quality of studies from quantitative and qualitative research designs. Further, our methodology involves a thorough and critical evaluation and summary of results for each objective. (Munn et al. 2018). This review identifies gaps and trends in current evidence, engaging in explicit discussions on contextual underpinnings to inform future research. In contrast to a scoping review, primarily identifying and mapping available evidence, it encompasses an explicit, transparent, peer-reviewed search strategy, mandatory critical appraisal (risk of bias assessment), synthesis of findings, and respective summary.

The review is not without its limitations. Most of the included studies were cross-sectional and presented findings on the prevalence of the conditions, thus limited in establishing the temporal sequence of causation between the factors and the perinatal mental ill-health. Consequently, the available evidence from these studies might not explicitly reflect the health impacts of perinatal mental disorders. Although a handful of prospective studies exist, findings from these studies were limited by the inability to disentangle pre-existing (mental) health problems, less reliability and validity from limited sample size, lack of appropriate comparison groups and a brief period of follow-ups, making it difficult to disentangle the antecedents of perinatal mental disorders from potential outcomes. Typically, these studies lack control for other health-related behaviors and may have limited information about the relevant temporal sequence between risk factors and outcomes, thus limiting the capacity to make causal inferences. Selection bias is a concern since this synthesis has been restricted to the available literature published in English. Moreover, studies in this field have been challenged by unreliable definitions and measurements of perinatal mental disorders and related health outcomes. For example, most of the measurement tools employed in the included studies were not validated in African contexts. Methodologies used to examine perinatal mental disorders and associated adverse consequences have frequently been criticized for inconsistencies in measurement, recall, selection and social desirability bias. The findings of this review are also largely derived from quantitative data, with only two eligible qualitative studies and limited data from qualitative components in mixed-methods studies, primarily intended to complement quantitative findings rather than offering substantial qualitative data. Furthermore, while the review protocol included the incorporation of grey literature to thoroughly retrieve relevant data, this study was unable to retrieve pertinent data from grey literature, potentially leading to an underrepresentation of prevalence data that frequently remain unpublished in peer-reviewed journals. The great majority of literature included in this review was from the three regions of Africa (East, West, and South). Thus, findings may not be representative of all countries in Africa. Moreover, employing PRISMA guidelines in this systematic review, which predominantly comprised observational epidemiological prevalence studies with a minority of intervention studies, ensures thorough reporting but lacks inherent assessment of individual study quality, addressing publication bias, interpreting substantial heterogeneity, and handling diverse study designs. Additional tools, such as the JBI Critical Appraisal Checklist for quality assessment (2020) or specific tools designed for assessing such studies (Munn et al. 2015; Munn et al. 2014), were necessary. Notably, quality assessment for included studies in this review utilized NIH and CASP tools for quantitative and qualitative studies, respectively. Although the scope of this paper is comprehensive, there is a discernible potential for at least two distinct papers to emerge from this review. However, the existing body of literature is notably scarce in providing substantial data on health-seeking behaviors, the nature and caliber of mental health services available and accessible for perinatal women, and the short- and long-term effects of maternal mental health on various domains such as maternal wellbeing, and pregnancy, neonatal and child health outcomes. Despite these limitations, this review provides important knowledge and data on perinatal mental health, and the epidemiology of perinatal disorders and their drivers in Africa.

## Conclusions

Detailed statistics on burden of perinatal mental health problems/disorders are important either to contribute to the public debate or to provide intelligence to guide public health policy and resource allocation. Without such data, many government agencies are subject to a “blind spot” in the planning of a range of community services for mothers who are at risk of mental health problems.

From our analysis of available evidence, the following evidence generation activities need to be increased in the African continent:

1. Determine the prevalence of antepartum and postpartum depression and depressive symptomatology among women in more settings, including in countries currently left out in the literature and focusing on critical populations such as women who experience stillbirths and adolescents.
2. Identify the broad range of risk and protective factors for depression across populations, including scrutiny of broader social and structural determinants of health, explicit exploration of systemic issues, such as healthcare access, policy frameworks, and societal norms.
3. Investigate short- and long-term effects of maternal mental health on maternal wellbeing, and pregnancy, neonatal and child health outcomes to enhance our understanding and contribute meaningfully to the existing knowledge base.
4. Ascertain routine practices for mental health screening for mothers at primary healthcare facilities.
5. Test validity and reliability of the Edinburgh Depression Scale (antepartum and postpartum) and the DSM IV-based MINI as culturally acceptable screening tools.
6. Test acceptability and feasibility of a systematic screening process in routine care with health providers and assess potential referral strategies for diagnosis and treatment.

## Supplementary Information

Below is the link to the electronic supplementary material.

Supplementary file1 (DOCX 14 KB)

Supplementary file2 (DOCX 111 KB)

Supplementary file3 (XLSX 32 KB)

Supplementary file4 (XLSX 12 KB)

## Data Availability

All data generated or analysed during this systematic review are included in this submitted manuscript [and its supplementary files].

## Abbreviations

ANC: Antenatal care
BDI: Beck’s Depression Inventory
BSI: Brief Symptom Index
CASP: Critical Appraisal Skill Program
CES-D: Center for Epidemiological Studies Depression Scale
CIDI: Composite International Diagnostic Interview
DASS: Depression, Anxiety, and Stress Scale
EPDS: Edinburgh Postnatal Depression Scale
ESA: Eastern and South Africa
GAD: Generalized anxiety disorder
HADS: Hospital Anxiety and Depression Scale
KPDS: Kessler Psychological Distress Scale
LMICs: Low- and middle-income countries
MINI: Mini-International Neuropsychiatric Interview
NIH: National Institutes of Health
PASS: Perinatal Anxiety Screening Scale
PHQ: Patient Health Questionnaire
PRAQ-R: Pregnancy-Related Anxiety Questionnaire-Revised
PRISMA: Preferred Reporting Items for Systematic Reviews and Meta-analyses
SDG: Sustainable Development Goal
STAI: State-Trait Anxiety Inventory
STI: Spielberger Trait Inventory
WCA: West and Central Africa
WHO-SRQ: WHO Self-Reported Questionnaire
WHO-DAS: WHO Disability Assessment Schedule
WMH: World Mental Health
ZSAS: Zung Self-rating Anxiety Scale
