# Supplementary material for: The epidemiology of maternal mental health in Africa: a systematic review
Source: Arch Womens Ment Health. 2025 Apr 12;28(5):997–1089. doi: 10.1007/s00737-025-01563-4 (PMC12436556; doi:10.1007/s00737-025-01563-4)
Supplement: Supplementary file 1 — Supplementary file1 (DOCX 14 KB) [file 737_2025_1563_MOESM1_ESM.docx]

**SEARCH STRATEGY**

**PUBMED**

Africa[Mesh] OR Africa [Title/Abstract] OR Algeria*[Title/Abstract] OR Angola[Title/Abstract] OR Benin[Title/Abstract] OR Botswana[Title/Abstract] OR Burkina Faso[Title/Abstract] OR Burundi[Title/Abstract] OR “Cabo Verde” [Title/Abstract] OR Cameroon[Title/Abstract] OR “Central African Republic”[Title/Abstract] OR Chad[Title/Abstract] OR [Title/Abstract] OR Comoros[Title/Abstract] OR Congo[Title/Abstract] OR Congo[Title/Abstract] OR Cote d'Ivoire[Title/Abstract] OR Djibouti[Title/Abstract] OR Egypt[Title/Abstract] OR Equatorial Guinea[Title/Abstract] OR Eritrea[Title/Abstract] OR Eswatini[Title/Abstract] OR Ethiopia*[Title/Abstract] OR Gabon[Title/Abstract] OR Gambia[Title/Abstract] OR Ghana*[Title/Abstract] OR Guinea[Title/Abstract] OR Guinea-Bissau[Title/Abstract] OR Kenya[Title/Abstract] OR Lesotho[Title/Abstract] OR Liberia[Title/Abstract] OR Libya[Title/Abstract] OR Madagascar[Title/Abstract] OR Malawi[Title/Abstract] OR Mali[Title/Abstract] OR Mauritania[Title/Abstract] OR Mauritius[Title/Abstract] OR Morocco[Title/Abstract] OR Mozambique[Title/Abstract] OR Namibia[Title/Abstract] OR Niger[Title/Abstract] OR Nigeria[Title/Abstract] OR Rwanda[Title/Abstract] OR (Sao Tome AND Principe)[Title/Abstract] OR Senegal[Title/Abstract] OR Seychelles[Title/Abstract] OR Sierra Leone[Title/Abstract] OR Somalia[Title/Abstract] OR “South Africa*”[Title/Abstract] OR “South Sudan”[Title/Abstract] OR Sudan[Title/Abstract] OR Tanzania[Title/Abstract] OR Togo[Title/Abstract] OR Tunisia[Title/Abstract] OR Uganda[Title/Abstract] OR Zambia[Title/Abstract] OR Zimbabwe[Title/Abstract]

AND

"Mental Health"[Mesh] OR "Mental Disorders"[Mesh] OR “Mental health”[Title/Abstract] OR “Mental disorders”[Title/Abstract] OR Anxiety[Title/Abstract] OR Depress*[Title/Abstract] OR dysthymi*[Title/Abstract] OR Psychosis [Title/Abstract] OR "obsessive-compulsive disorder"[Title/Abstract] OR "post-traumatic stress disorder" OR "Bipolar Disorder*"[Title/Abstract] OR "Mental Illness"[Title/Abstract] OR "Post-Traumatic Stress Disorder*"[Title/Abstract] OR psychiatric[Title/Abstract] OR psychotrauma[Title/Abstract] OR “emotional disorder*”[Title/Abstract]

AND

"Maternal Health Services"[Mesh] OR "Prenatal Education"[Mesh] OR "Delivery, Obstetric"[Mesh] OR "maternal health services"[Title/Abstract] OR "antenatal care"[Title/Abstract] OR antenatal[Title/Abstract] OR "prenatal care"[Title/Abstract] OR "assisted delivery"[Title/Abstract] OR prenatal[Title/Abstract] OR "maternal-child health services"[Title/Abstract] OR postnatal[Title/Abstract] OR postpartum[Title/Abstract] OR "postpartum care"[Title/Abstract] OR obstetric*[Title/Abstract] OR pregnan*[Title/Abstract] OR "reproductive age"[Title/Abstract]

**EMBASE**

'psychotrauma'/exp OR psychotrauma:ti,ab,kw OR psychiatric:ti,ab,kw OR 'emotional stress'/exp OR 'emotional stress':ti,ab,kw OR 'emotional disorder'/exp OR 'emotional disorder':ti,ab,kw OR 'neurosis'/exp OR neurosis:ti,ab,kw OR 'obsessive compulsive disorder'/exp OR 'obsessive compulsive disorder':ti,ab,kw OR depress*:ti,ab,kw OR 'depression'/exp OR 'mental disease'/exp OR 'mental disease':ti,ab,kw OR 'anxiety'/exp OR 'anxiety':ti,ab,kw OR 'anxiety disorder'/exp OR 'anxiety disorder':ti,ab,kw OR 'mental health'/exp OR 'mental health':ti,ab,kw

AND

'maternal health service'/exp OR 'maternal health service':ti,ab,kw OR 'obstetric delivery'/exp OR 'obstetric delivery':ti,ab,kw OR 'prenatal care'/exp OR 'prenatal care':ti,ab,kw OR 'postnatal care'/exp OR 'postnatal care':ti,ab,kw OR 'perinatal care'/exp OR 'perinatal care':ti,ab,kw OR 'child health care'/exp OR 'child health care':ti,ab,kw OR 'childbirth education'/exp OR 'childbirth education':ti,ab,kw OR 'maternal care'/exp OR 'maternal care':ti,ab,kw

AND

'africa'/exp OR africa OR algeria OR angola OR benin OR botswana OR (burkina AND faso) OR burundi OR (cabo AND verde) OR cameroon OR 'central african republic' OR chad OR comoros OR congo OR 'cote divoire' OR djibouti OR egypt OR (equatorial AND guinea) OR eritrea OR eswatini OR ethiopia OR gabon OR gambia OR ghana OR guinea OR 'guinea bissau' OR kenya OR lesotho OR liberia OR libya OR madagascar OR malawi OR mali OR mauritania OR mauritius OR morocco OR mozambique OR namibia OR niger OR nigeria OR rwanda OR 'sao tome and principe' OR senegal OR seychelles OR 'sierra leone' OR somalia OR 'south africa' OR 'south sudan' OR sudan OR tanzania OR togo OR tunisia OR uganda OR zambia OR Zimbabwe

**PSYCINFO**

(maternal health service' or 'obstetric delivery' or 'prenatal care' or 'prenatal care' or 'postnatal care' or 'perinatal care' or 'child health care' or 'childbirth education' or 'maternal care' or 'reproductive age' or 'pregnan*).mp.

AND

africa or africa or algeria or angola or benin or botswana or (burkina and faso) or burundi or (cabo and verde) or cameroon or 'central african republic' or chad or comoros or congo or 'cote divoire' or djibouti or egypt or (equatorial and guinea) or eritrea or eswatini or ethiopia or gabon or gambia or ghana or guinea or 'guinea bissau' or kenya or lesotho or liberia or libya or madagascar or malawi or mali or mauritania or mauritius or morocco or mozambique or namibia or niger or nigeria or rwanda or 'sao tome) and principe') or senegal or seychelles or 'sierra leone' or somalia or 'south africa' or 'south sudan' or sudan or tanzania or togo or tunisia or uganda or zambia or Zimbabwe).mp.

AND
('psychotrauma' or psychiatric or 'emotional stress' or 'emotional disorder' or 'neurosis' or 'obsessive compulsive disorder' or depress* or 'mental disease' or 'anxiety' or 'anxiety disorder' or 'mental health').mp.
