# Supplementary material for: The epidemiology of maternal mental health in Africa: a systematic review
Source: Arch Womens Ment Health. 2025 Apr 12;28(5):997–1089. doi: 10.1007/s00737-025-01563-4 (PMC12436556; doi:10.1007/s00737-025-01563-4)
Supplement: Supplementary file 2 — Supplementary file2 (DOCX 111 KB) [file 737_2025_1563_MOESM2_ESM.docx]

| Supplementary Table 1: Maternal mental health problems in Africa: study characteristics by study design | | | | | | | | | |
| --- | --- | --- | --- | --- | --- | --- | --- | --- | --- |
|  | | | | | | | | | |
| Author, year | **Country** | **Study settings (urban, rural, peri-urban, health facilities)** | **Study population and sample size** | **Age (range, mean) in years** | **Study aim/objective** | **Data sources (e.g., health facilities, etc.) or secondary (e.g., record reviews, surveys (DHS, SPA, MICs, etc.))** | **Sampling strategy** | **Stage of pregnancy (prenatal, postnatal)/non-pregnant** | **Analysis** |
| Case review and case-control studies | | | | | | | | | |
| Ndukuba et al., 2015 | Nigeria | Federal Neuropsychiatric Hospital Enugu | 76 women diagnosed with postpartum psychiatric conditions in a federal hospital | Mean age = 27.76 | Determine the clinical and socio‑demographic characteristics of women diagnosed with postpartum psychiatric conditions in a tertiary mental health facility of a developing country | Primary data | NR | Postpartum | Chi-square |
| Idowu et al., 2022 | Nigeria | Ekiti State | 198 participants (99 HIV-positive and 99 HIV-negative women) | 34.2 ± 4.1 for HIV positives vs 32.0 ± 3.5 for HIV negatives | Examine depression and anxiety disorders in pregnant HIV-positive women in response to the COVID-19 pandemic | Primary data | Simple random sampling | NR | Mann–Whitney U-test or Chi-square tests |
| Ghoneim et al., 2021 | Egypt | Suez (Urban) | 316 women (158 case and 158 in control groups) | 18-45 | Determine the relation between intimate partner violence and depression during pregnancy. | Primary data | 1 to 1 matching | Second and third trimesters | Logistic regression |
| Mnisi et al., 2019 | South Africa | Pretoria, South Africa (urban). | 2,671 postpartum mothers | 17-41; mean = 27.96 | Determine the prevalence and identify risk factors for postnatal depression in mothers whose babies were admitted to the neonatal unit | Primary data | Systematic | Between 26- and 42-weeks’ gestation (preterm) | Logistic regression model |
| Time-series studies | | | | | | | | | |
| Rencken et al., 2022 | South Africa | KwaZulu-Natal Province (urban) | 132 pregnant women | 21-39 (mean = 30) | Describe prevalence of postnatal depression in HIV-positive and HIV-negative mothers delivering healthy singleton infants | Primary data | NR | Within 6 months of delivery | Chi-square |
| Cross-sectional studies | | | | | | | | | |
| Ngocho et al., 2022 | Tanzania | Moshi municipality in northern Tanzania | 1,033 pregnant women 18 years and above | 18 and above (mean = 25) | Quantify depressive symptoms and factors associated with depression among pregnant women in Moshi, Tanzania | Secondary analysis of baseline data from randomized controlled trial on HIV reduction intervention in antenatal care | NA | NR | Log binomial regression model |
| Umuziga et al., 2022 | Rwanda | Southern Province in Rwanda | 396 pregnant women receiving ANC services and in their 6^th^ months and above | 16 to 45 (mean = 30.8) | Examine the relationship between antenatal depressive symptoms and social support across several relationships among women attending ANC | Analysis of baseline data from a quasi-experimental study | Simple random sampling | Antenatal: 6th months or above | Logistic regression model |
| Tilahun et al. , 2022 | Ethiopia | Adisa Ababa, Ethiopia | 615 postnatal mothers seeking PNC care and services within 6 weeks postpartum | 18 and above | Magnitude and associated factors of suicidal behavior among postpartum mothers | Primary data | Multistage sampling techniques | Within 6 weeks of postpartum | Logistic regression model |
| Gebremichael et al., 2017 | Ethiopia | Rural and semi-urban | 728 pregnant mothers and mothers having a child less than one year in Southern Ethiopia | 15+; mean 28.02 ± 5.8 | Assess the perinatal depression and associated factors among mothers in Southern Ethiopia | Primary data | Random cluster sampling | Perinatal | Multivariable logistic regression |
| Onah et al., 2017 | South Africa | Low-income, residential and industrial suburb within the City of Cape Town | 376 women visiting midwife obstetric unit for antenatal care | > 18 | Investigate the prevalence and associated psychiatric and socio-economic contextual factors for SIB among pregnant women living in low-resource communities in South Africa | Primary data | Systematic sampling technique | Perinatal | Logistic regression |
| Mossie et al., 2017 | Ethiopia | Maichew Town | 196 pregnant women | 16+ | Assess the magnitude of antenatal depressive symptoms and associated factors among women at Maichew Town, North Ethiopia | Primary data | Systematic random | Antenatal | Multivariable logistic regression |
| Belay et al., 2018 | Ethiopia | Dubti hospital | 357 pregnant women attending ANC | < 20 | Assess prevalence of antenatal depression and associated factors among Dubti Hospital Antenatal care attendants | Primary data | Systematic random sampling | Antenatal | Logistic regression |
| Brittain et al., 2017 | South Africa | Gugulethu in Cape Town, South Africa | 623 HIV-infected pregnant women initiating ART | > 18 | Examine factors associated with social support and stigma among pregnant women initiating antiretroviral therapy in the Western Cape | Primary data | NR | Antenatal | Linear regression |
| Natamba et al., 2017 | Uganda | Gulu Regional Referral Hospital | 403 pregnant women recruited from the ANC of Gulu Regional Referral Hospital | 24.7 ± 5.0 | Assess association between FI and depressive symptoms severity and assessed whether such an association varied among Ugandan pregnant women | Primary data | NR | Antenatal | Linera regression |
| Barthel et al., 2017 | Ghana & Cote dvoire | urban | 776 women in their last trimester of pregnancy - Centre de SanteÂ Urbain à base communautaire d'Abobo in Abidjan (CIV) or at the Komfo Anokye Teaching Hospital in Kumasi | 28.9 (5.5; 18 to 46) | Investigate the long-term course of maternal depressive symptoms and its association with various mother- and child-related characteristics in two West African lower middle-income countries with a focus on the relationship with long-term anxiety symptoms | Primary data | NR | Antenatal | Logistic regression |
| Rurangirwa et al., 2018 | Rwanda | Northern Province of Rwanda and Kigali city (urban, semiurban and rural) | 921 women who gave birth 1–13 months before the interview | 15-47 | Investigate the prevalence of non-psychotic mental health disorders (MHDs) and the association between exposure to all forms of intimate partner violence (IPV) during pregnancy and MHDs | Primary data | Simple random sampling | Postpartum | Multivariable logistic regression |
| Mbawa et al., 2018 | Zimbabwe | Bulawayo provinces of Zimbabwe | 50 adolescent mothers and 50 adult mothers (first pregnancy after 18th birthday) | adolescent < 19; adults > 19 | Determine PPD among adult mothers relative to adolescent mothers | Primary data | NR | Postpartum | NR |
| Odinka et al., 2018 | Nigeria | urban | 309 low-risk, postpartum women in Enugu, Southeastern Nigeria | 20–46; mean + SD (29.65 ± 4.87) | Assess the prevalence of PPD and anxiety, and to investigate their relationship with marital satisfaction | Primary data | NR | Postpartum | Chi-squaare/ correlations |
| Shifa et al., 2018 | Ethiopia | Gamo Gofa Zone, Southern Ethiopia | 356 mothers who lost their children (exposed) and 712 mothers with alive children (unexposed); matched for date of birth of the index child | NR | Examine the association between child death and maternal mental distress | Primary data | Purposive and census | Postpartum | Weighted conditional logistic regression |
| Harrington et al., 2018 | Malawi | Government ANC | 299 women initiating antiretroviral therapy for HIV | Median 26 (22-30) | Estimate the prevalence and incidence of probable perinatal depression among women enrolled in Option B+ antenatal HIV care in Malawi | Primary data | NR | Antenatal and postnatal | Kappa statistics |
| Toru et al., 2018 | Ethiopia | Mizan-Aman town | 460 postpartum women who gave birth within 12 months before data collection | 18-39 | Assess the magnitude of depression and associated factors among postpartum women in Mizan Aman town, Bench Maji Zone, Southwest Ethiopia 2017 | Primary data | Random sampling | Postpartum | Logistic regression |
| Anokye et al., 2018 | Ghana | Urban, teaching hospital | 212 mothers who were within 12 months after delivery | > = 18; Mean = 27.3; SD = 8.31 | Determine the prevalence of postpartum depression and interventions utilized for its management in a Health facility in Ghana. | Primary data | Simple random sampling | Postpartum | Logistic regression |
| Osok et al., 2018 | Kenya | Community health center | 176 adolescent pregnant women | 15-18 | Determine the prevalence of depression and related psychosocial risks among pregnant adolescents reporting at a maternal and child health clinic in Nairobi, Kenya | Primary data | NR | Antenatal | Hierarchical multivariate linear regression |
| Rotheram-Fuller et al., 2018 | South africa | peri-urban township neighborhoods in Cape Town | 1,238 pregnant women | 26.4 (SD = 5.6) | Determine the consequences of maternal depressed mood on children’s growth, health, cognitive and language development | Primary data | NR | Antenatal and postnatal | Mixed-effects linear regression models |
| MacGinty et al., 2017 | South africa | peri-urban area in Paarl | 1,137 pregnant women over 18-year-old, between 20 and 28 weeks’ gestation, and enrolled in a South African parent study, the Drakenstein Child Health Study (DCHS) | > 18; median 26 (22.3-31.1) | Assess the association between maternal mental health and childhood wheezing has not been well studied in low and middle-income countries (LMIC), such as South Africa | Primary data | NA | Antenatal and postnatal | Logistic regression |
| Shitu et al., 2019 | Ethiopia | Ankesha District, Northwest Ethiopia | 596 postpartum mothers | 30.57 (SD ± 6.3) | Assess postpartum depression and associated factors among mothers who gave birth in the last twelve months in Ankesha district, Awi zone, Northwest Ethiopia | Primary data | Cluster sampling | Postpartum | Logistic regression |
| January et al., 2018 | Zimbabwe | Rural | 192 mothers who attended postnatal services 7 days and 42 days post-delivery at postnatal clinics | 23.7 (6.14) | Explore the prevalence and associated factors of PND among women attending postnatal care services in two rural districts of Chipinge and Mutasa, Manicaland, Zimbabwe between August and September 2017 | Primary data | Consecutively sampling | Postpartum | Logistic regression |
| Chorwe-Sungani et al., 2018 | Malawi | antenatal clinics in Blantyre district, Malawi | 480 women attending ANC | 18 to 43 (mean 25.2 ± 5.5) | Describe demographic, clinical and risk profile of antenatal depression among pregnant women attending antenatal clinics in Blantyre district, Malawi | Primary data | NR | Postpartum | Logistic regression |
| Rwakarema et al., 2015 | Tanzania | Antenatal clinics in Mwanza city (urban) | 369 pregnant women who attended ANC | median age was 26 (range 18 to 42) | Assess depression in pregnancy and related psychosocial risk factors among select pregnant women residing in Mwanza region, Northern Tanzania. | Primary data | Systematic random | Antenatal | Logistic regression |
| Tomita et al., 2015 | South Africa | Urban | 651 postpartum women | 15+ | Explore the relationship between depression before pregnancy and the low birth weight (LBW) of infants in post-apartheid South Africa | Secondary data (Waves 1 and 2 of the South African National Income Dynamics Study) | NR | Pre-pregnancy | Logistic regression |
| Jebena et al., 2015 | Ethiopia | Jimma Zone, urban | 660 pregnant women | 15+; mean (SD)=25.5 (± 4.9). | Assess household food insecurity and mental distress among pregnant women in Southwestern Ethiopia: a cross sectional study design | Primary data | Probability sampling | Antenatal | Logistic regression |
| Baumgartner et al., 2016 | Ethiopia | Amhara region | 1,319 postnatal non-pregnant women who had a delivery in the previous 24 months. | 15+ | Assess mental health among 1294 nonpregnant, postpartum women in Amhara region | Primary data | Random | Postpartum | Multivariate logistic regression |
| Bitew et al., 2016 | Ethiopia | Sodo district, Gurage Zone, southern Ethiopia. | 1,311 women in the second or third trimesters of pregnancy | 26,8 | Investigate whether maternal health care utilization varies as a function of antenatal depressive symptoms | Primary data | NR | Antenatal | Logistic regression |
| Baumgartner et al., 2014 | Ethiopia | Amhara region | 1,319 15–49 years old who had a delivery in the previous 24 months | 15-49 years | Describe the mental health status of women who had given birth in the last 24 months in the Amhara region of Ethiopia | Primary data | NR | Postpartum | Chi-square |
| Tefera et al., 2015 | Ethiopia | Selected kebele of Goba and Robe town | 357 pregnant women and women with under 1 year of child | 22.9 (SD ± 2.1) | Determine the prevalence of perinatal depression and its associated factors among reproductive age group women at Goba and Robe town of Bale zone; Oromia Region, Southeast Ethiopia | Primary data | Simple random sampling | Perinatal | Logistic regression |
| Steward et al., 2014 | Malawi | Mangochi district which lies at the southern end of Lake Malawi; maily rural | 98 women in pregnancy and postnatal period | NR | Investigate the thoughts and emotions experienced by women in pregnancy and the postnatal period, their expectations of support from husband and others, problems and difficulties faced and the impact of these on psychological wellbeing | Primary data | Purposive | Prenatal and Postpartum | Thematic analysis |
| Mohammed et al., 2014 | Egypt | Rural | 200 women who gave birth within the last 14 months preceding interview | aged between 19 and 45 (mean age 29 ± 5.2) | Examine prevalence of postpartum depression (PPD) in a certain rural area in Upper Egypt, identifying the factors that might be involved in its genesis | Primary data | Systematic random | Postpartum | Logistic regression |
| Stellenberg et al., 2015 | South africa | Rural | 159 mothers 18 years and older | 18+ | Determine the prevalence of PND and to identify the contributing risk factors associated with PND. | Primary data | Convenient | Postpartum | Bivariate |
| Sulyman et al., 2016 | Nigeria | federal tertiary health institution in northeastern Nigeria. | 483 women who delivered at the maternity unit of a tertiary health institution in northeastern Nigeria | 20+ | Determine the prevalence rate of postnatal depression and assess factors that are associated with its development among northeastern Nigerian women | Primary data | NR | Postpartum | Logistic regression |
| Peltzer et al., 2016 | South Africa | community health centers in Mpumalanga province | 663 HIV-positive prenatal women | 28.3 (SD = 5.7) | Assess the prevalence of depressed symptoms and associated factors in prenatal HIV-positive women in primary care facilities in rural South Africa | Primary data | Systematic random | Postpartum | Logistic regression |
| Abate et al., 2021 | Ethiopia | Amhara regional state | 291 HIV-positive pregnant women | 18 and above (30.37 ± 5.12). | Assess depression and its associated factors among HIV-positive pregnant women seeking antenatal care | Primary data | Census | Antenatal | Univariable and multivariable logistic regression |
| Abdelghania et al., 2021 | Egypt | Sharkia province | 875 pregnant women | 20-40 (28.7 ± 6.04) | Evaluate current suicide risk (CSR) and its correlates of among pregnant women in Egypt | Primary data | NR | Any trimester | Independent t-test, chi-square, fisher exact and Mann Whitney tests |
| Abebe et al., 2022 | Ethiopia | Adisa Ababa, Ethiopia | 368 HIV-positive pregnant women | 18 and above | Determine the prevalence of depression and its association with adherence to ART among HIV-positive pregnant women attending antenatal care | Both primary and secondary data | Review of secondary data and simple random sampling technique | Third trimester | Logistic regression model |
| Abrahams et al., 2022 | South Africa | Cape Town | 85 perinatal women | NR | Examine the association between common mental disorders (CMDs), food insecurity, and experiences of domestic violence | Both primary and secondary data | File review of secondary data and simple random sampling technique | Pregnancy to 3 months after delivery | Poisson regression model |
| Adebowale et al., 2020 | Nigeria | Benin-City, Edo State | 395 pregnant women | 16-49 | Identify the magnitude of IPV, and its relationship with psychiatric morbidity and partner alcohol use | Primary data | Systematic sampling | Prenatal | Binary logistic regression |
| Abdelhai et al., 2015 | Egypt | Cairo | 376 pregnant women | 27.8 +/-5.99 | Examine anxiety and depression among pregnant women, as well as identify the frequency and association of exposure to DV | Primary data | Systematic sampling | Any trimester | Binary logistic regression |
| Adjorlolo et al., 2022 | Ghana | Greater Accra and Central Regions | 589 pregnant women | 18 and above | Investigate prevalence and correlates of PLEs among pregnant women in Ghana | Primary data | Simple random sampling | Any trimester | Principal component analysis, MANOVA and multinomial logistic regression |
| Ahmed et al., 2021 | Egypt | eastern part of Assiut City | 257 postnatal mothers) | 27.98 ± 4.7 | Determine the prevalence of depression among postnatal mothers and evaluate the risk factors associated with depression | Primary data | Systematic sampling | Postnatal women attending child welfare clinics following 2, 4, and 6 months after delivery | Logistic regression model |
| Adeyemo et al., 2020 | Nigeria | Eti-Osa Local Government Area of Lagos State | 250 postnatal mothers | 18 and above (29.5 ± 5.70 SD) | Determine the prevalence of postpartum depression and its predictors among postnatal women | Primary data | Multistage sampling | 6 weeks postpartum | Chi-square and logistic regression |
| Abebe et al., 2019 | Ethiopia | Bahir Dar Town | 511 postnatal mothers | 18-39 (mean = 24.3 (± 3.83)) | Assess postpartum depressive symptoms among postpartum women | Primary data | Systematic sampling | Within 6 months of postpartum | Logistic regression model |
| Abadiga, 2019 | Ethiopia | Nekemte town, East Wollega zone, West Ethiopia, | 287 postnatal women | 15 and above (29.6 +/- 9.45 SD) | Assess the magnitude and associated factors of postpartum depression among women | Primary data | Simple random sampling | Within 12 months of delivery | Logistic regression model |
| Abegaz et al., 2022 | Ethiopia | Debre Markos town | 408 pregnant women | 18 and above | Assess the magnitude of pregnancy-related anxiety and associated factors among pregnant women attending an antenatal checkup | Primary data | Systematic random sampling | Antenatal | Bivariable and multivariable logistic regression |
| Adamu et al., 2018 | Ethiopia | Adisa Ababa, Ethiopia | 618 postpartum women | 15 and above | Identify predictors of postpartum depression | Primary data | Multistage sampling | Postpartum | Logistic regression model |
| Agbaje et al., 2019 | Nigeria | Nsukka and Obollo-Afor towns in Nsukka and Udenu Local Government Areas (LGAs), Enugu State. | 267 postnatal women | 15 and above | Identify the factors associated with depressive and anxiety symptoms in women | Primary data | Convenient sampling | Postpartum periods from 4 to 12 weeks | Binary logistic and multinomial logistic regressions |
| Desalegn et al., 2021 | Ethiopia | South Gondar zone, Amhara regional state, northwest Ethiopia | 606 HIV-positive pregnant women | 18 and above | Assess antenatal depression and associated factors among HIV-positive pregnant women attending prevention of mother to child transmission service | Primary and secondary data | Multistage sampling | Pregnant women at any trimester of pregnancy | Binary logistic regression |
| Tesfaye et al., 2021 | Ethiopia | Jimma Town | 314 pregnant women | 15 and above | Assess the prevalence of antenatal depression and associated factors among women attending antenatal care (ANC) service | Primary data | Systematic random sampling | Antenatal | Chi-square test |
| Mostafa et al., 2021 | Egypt | Shebien el Kanater (rural) | 232 pregnant women | 15–49 (mean = 25.9 ± 4.8) | Estimate the prevalence of antepartum depression and its effects on pregnancy outcomes among pregnant women in the last trimester | Primary data | Multistage sampling | Antenatal in the third trimester | Logistic regression model |
| Parcesepe et al., 2021 | Cameroon | Yaoundé, Cameroon | 230 pregnant women living with HIV | 18 or older | Estimate the prevalence and correlates of mental health disorders among pregnant women living with HIV | Secondary analysis of baseline data | NR | Third trimester of pregnancy | Multivariable logistic regression |
| Bante et al., 2021 | Ethiopia | Arba Minch Zuria district, Gamo zone | 667 pregnant women | Mean age (±SD) was 27.4 (± 6) | Assess comorbidity anxiety and depression (CAD) and associated factors among pregnant women | Primary data | Cluster sampling | Any trimester | Logistic regression model |
| Beyene et al., 2021 | Ethiopia | Debre Tabor and Woreta towns, Northeast Ethiopia (urban) | 548 pregnant women | NR | Assess the prevalence and potential predictors of antenatal depression | Primary data | Cluster sampling | Any trimester | Logistic regression model |
| Dadi et al., 2021 | Ethiopia | Gondar town (urban) | 866 mother-infant dyads | 18 and above (mean (SD) = 26.5 (4.5)) | Estimate associations between perinatal depression and infant diarrhea, acute respiratory infection (ARI), and malnutrition | Primary data | Systematic | Second to third trimester | Generalized Structural Equation Models (GSEM), generalized estimating equation (GEE) model with a Poisson link function and exchangeable correlation structure |
| Ogueji, 2021 | Nigeria | Akwa Ibom, Benue, and Rivers States of Nigeria | 840 HIV-positive pregnant women | (age range 22–46; mean = 38.86 + 13.0) | Explore the experiences and predictors of psychological distress in pregnant women living with HIV | Primary data | Convenient/purposive | NR | Linear regression and thematic analysis |
| Wycliffe et al., 2021 | Kenya | Nairobi, Kenya (urban) | 59 postpartum women | 18-41 | Establish factors contributing to emotional distress among postpartum mothers with newborns | Primary data | Simple random sampling | NR | Descriptive statistics |
| Bishaw et al., 2022 | Ethiopia | east Gojjam zone of Debre Markos town (urban) | 847 pregnant women | 18 and above (mean (SD) = 27.57±6.08)) | Assess the prevalence and factors associated with generalized anxiety disorder among pregnant women attending antenatal care | Primary data | Multistage sampling | Any trimester | Logistic regression |
| Zotova et al., 2022 | Congo | Kinshasa, Congo | 1,392 pregnant women | NR | Assess the association between HIV disclosure and depressive symptoms among pregnant WLHIV and examine whether the knowledge of partner’s status or recent IPV modified this association | secondary analysis of randomized trail data | NA | Any stage of pregnancy and postpartum mothers within 12 months of delivery | Linear model |
| Beketie et al., 2021 | Ethiopia | Arba Minch town | 316 pregnant mothers | 18 and above (25.8 (± 5.4 SD)) | Assess the magnitude and associated factors of antenatal depressive symptoms among pregnant women | Primary data | Systematic sampling | Any trimester of pregnancy | Logistic regression |
| Wake et al., 2022 | Ethiopia | Addis Ababa, Ethiopia | 461 postnatal mothers | 15 and above | Assess the magnitude and determinant factors of postpartum depression among mothers attending their postnatal and vaccination services | Primary data | Multistage sampling | Within 6 weeks after delivery | Binary logistic regression |
| Borie et al., 2022 | Ethiopia | Sodo town, Addis Ababa | 309 pregnant mothers | 14 and above | Assess the prevalence of maternal depression and associated factors among antenatal care attendants | Primary data | Systematic random | Any trimester | Binary logistic regression |
| Jidong et al., 2021 | Nigeria | Jos in Plateau state (Urban) | 40 postpartum mothers | 18-43 | Explore mental health lived experiences of mothers in Jos, Nigeria | Primary data | Purposive and snowball sampling | Postnatal between 3 and 48 months | Thematic analysis |
| Madeghe et al., 2021 | Kenya | Urban low-income settlement in Nairobi | 262 pregnant women | mean age (SD) = 25.3 + 5.0; range: 18 to 44 | Determine the associations between nutrition status, dietary intake, and maternal depression among pregnant women | Primary data | Consecutive | Any trimester | Logistic regresson |
| Ayele et al., 2021 | Ethiopia | Gondar town (urban) | 409 pregnant mothers | 15 and above | Investigate the prevalence and associated factors of antenatal depression among pregnant mothers who had intimate partner violence during pregnancy | Primary data | Consecutive | Any trimester | Binary logistics regression |
| Tamiru et al., 2022 | Ethiopia | Kersa and Haramaya (rural) | 1,034 pregnant women | age range (20–35) with a mean of 30.1 (SD = 8.5) | Determine the prevalence of common mental disorders and associated factors among pregnant mothers | Primary data | Simple random | Any trimester | Logistic regression |
| Jihed et al., 2022 | Tunisia | Sousse, Tunisia (urban) | 389 pregnant women | Mean age = 30.1 +/- 6.4 | Determine the prevalence of major depressive disorder (MDD) and its associated factors in working pregnant women. | Primary data | NR | Second trimester | Logistic regression |
| Oladeji et al., 2022 | Nigeria | Ibadan, Nigeria (urban) | 1,359 pregnant adolescent mothers | 14-20; mean = 18.4 (1.0 SD) | Examine the prevalence and clinical and psychosocial factors associated with depression and depression severity in pregnant adolescents | Secondary data analysis | NR | Any trimester | Logistic regression |
| Kugbey et al., 2021 | Ghana | Hohoe Municipal (urban) | 214 pregnant women | 18 and above | Examine the prevalence and correlates of prenatal depression, anxiety and current suicidal behaviors among pregnant women | Primary data | Consecutive | Any trimester | Logistic regression |
| Belete et al., 2021 | Ethiopia | Hawassa city (Urban) | 738 pregnant women | 15-49; mean = 25.5(4.12) | Assess the prevalence and factors associated with suicide ideation and attempt among pregnant women attending antenatal care services | Primary data | Systematic sampling | Any trimester | Chi-square and binary logistic regression |
| Keliyo et al., 2021 | Ethiopia | Faafan zone of Ethiopian Somali region | 395 pregnant women | 15-40; mean = 25.71 (SD ± 5:04) | Assess the prevalence of antenatal depression and its associated factors among pregnant women | Secondary data analysis | NA | Any trimester | Binary logistic regression |
| Nwafor et al., 2021 | Nigeria | Abakaliki (urban) | 456 pregnant women | 18-45 (mean = 27 ± 12.6) | Determine prevalence and predictors of COVID-19-related depression, anxiety and stress symptoms among pregnant women | Primary data | Convenient | Any trimester | Logistic regression |
| Sewnet et al., 2020 | Ethiopia | Amhara regional state | 422 pregnant women | 15 and above; mean age = 28 (SD ± 4.86) | Assess the prevalence of depression during the COVID-19 pandemic in pregnant women and its associated factors | Primary data | Systematic random sampling | Any trimesters | Logistic regression |
| Barsisa et al.,2021 | Ethiopia | Arbaminch Town (urban) | 776 women with under-five children | 18 and above; mean (SD) age was 30 (5.9) | Assess the prevalence and factors associated with common mental disorder among mothers of under-five year children | Primary data | Systematic random sampling | Mothers with children under-five | Logistic regression |
| Atuhaire et al., 2021 | Uganda | Kampala, the capital city of Uganda | 292 pregnant women | 15-49 | Determine the prevalence and understand the factors associated with Postpartum depression among mothers | Primary data | Stratification | 6 to 8 weeks’ postpartum periods | Chi-square and binary logistic regression |
| Bhushan et al., 2022 | Malawi | Not reported | 545 pregnant women | NR | Understand how depression changes over the perinatal period, varies across treatment and retention groups | Review of secondary data | NR | Antenatal | Multilevel generalized linear models (MLM) |
| Mokwena et al., 2021 | South Africa | Umhlathuze District, KwaZulu Natal (urban) | 386 HIV-positive women | 16-42 (mean = 29) | Determine sociodemographic factors associated with the prevalence of postnatal depression symptoms among a sample of HIV-positive women attending health services | primary data | NR | Postnatal, 1–12 weeks after delivery | Chi-square test and logistic regression model |
| Anbesaw et al., 2021 | Ethiopia | Jimma Town (urban) | 415 pregnant women | 18 and above 25.22 (± 4.62) | Explore the prevalence of suicidal ideation and associated factors among pregnant women attending antenatal care | Primary data | Systematic | Gestational age of 30 days and above | Logistic regression model |
| Kim et al., 2021 | Ghana | 32 communities (clusters) in West Mamprusi District and Nabdam District in Upper East Region | 313 pregnant women | 16 and older; mean = 26.8(6.6) | Examine the effects of antenatal depression and women’s perceived health during the antenatal period on maternal health service utilization in rural northern Ghana | secondary analysis of clustered-randomized data | NR | Any trimester | Linear regression |
| Mwita et al., 2021 | Tanzania | Lake and Western zones of Tanzania (urban) | 345 pregnant women | 20-47 (mean = 30.35) | Determine the prevalence and risk factors associated with depression among pregnant women attending antenatal clinic | Primary data | Census | Any trimester | Logistic regression |
| Oboro et al., 2022 | Nigeria | southern part of Nigeria | 511 pregnant women | 18-43 (mean = 27.9 (± 6.01) | Determine the prevalence and factors associated with antenatal depression in first half of pregnancy | Primary data | NR | Within first half of pregnancy (< 20 weeks) | Logistic regression model |
| Jones et al., 2021 | Inter-country study including Kenya, Tanzania, Uganda, and Nigeria | multicenter | 1,333 pregnant women | 18-44 | Assess prevalence of depression among WLWH enrolled in the African Cohort Study (AFRICOS) by pregnancy status and describe factors associated with depression | Secondary analysis of longitudinal study data | NR | NR | Logistic regression with generalized estimating equations |
| Masiano et al., 2022 | Malawi | Three sites: Two large urban health centers in Lilongwe, and Mangochi, a busy rural district hospital | 798 pregnant women living with HIV | 18 and above (mean = 27.45 (SD=6.59) | Assess the prevalence of adverse childhood experiences and their association with common mental disorders among pregnant women living with HIV | Secondary analysis of randomized trial data | NR | Any trimester | Poisson regression model |
| Redinger, et al., 2021 | South Africa | Soweto (urban) | 649 pregnant women | 18 or older (mean = 29.6 (SD +/- 5.9) | Examine rates of Thought of self-harm across pregnancy and investigate factors associated with the onset and persistence of TSH, as well as the relationship between TSH, depression and/or anxiety. | Secondary analysis of cohort study data | NR | < 20 weeks of gestational age | Logistic regression |
| Khan et al., 2020 | Mozambique | Maputo and Nampula (semi-urban) | 853 women | 18-49 (mean = 25.9 (5.9)) | Compare the frequency of CMD and suicide risk in a sample of women presenting or accompanying patients in primary care in two Mozambican semi-urban settings | Primary data | NR | pregnant women in reproductive age, those with child under one year and those with children more than a year | ANOVA, Chi-square and logistic regression |
| Kaiyo-Utete et al., 2020 | Zimbabwe | Harare District (urban) | 375 pregnant women | 18-48 | Assess prevalence of and associated factors for antenatal depression among pregnant women attending antenatal care services | Primary data | Simple random | second trimester through to 6 weeks post-delivery. | Chi-square and logistic regression |
| MacGinty et al., 2020 | South Africa | Drakenstein subdistrict of the Western Cape Province, South Africa (periurban) | 961 pregnant women | 18 and above (mean = 26 (5.70)) | Identify risk factors for antenatal maternal psychological distress and determine whether antenatal maternal psychological distress was associated with infant birth and developmental outcomes. | Analysis of baseline/primary data from a cohort study | NR | Between 20 and 28 weeks of gestation | Fisher exact, Chi-square and Wilcoxon Rank sum tests. Linear and logistic regression models |
| Anato et al., 2020 | Ethiopia | Kebeles district (rural areas) | 232 mother–infant pairs | Age group not reported; (mean = 28.0 (6.6)) | Assess the association between maternal postpartum depression symptoms and infant (5–10 months of age) stunting in northern Ethiopia | Primary data | Multi-stage sampling | NR | Logistic regression model |
| Akinsulore et al., 2021 | Nigeria | Ile-Ife (urban) | 230 pregnant women | 18-45; mean = 28.2, SD = 5.4) | Investigate pregnancy-related anxiety symptoms and associated factors among pregnant women | Primary data | NR | Any trimester | Hierarchical logistic regression model |
| Gebregziabher et al., 2020 | Ethiopia | Central Region of Eritrea | 380 postpartum mothers | 16-43 (mean = 27.7 (SD ± 5.23)) | Assess the magnitude and predictors of postpartum depression among mothers in Central Region of Eritrea | Primary data | Simple random sampling | Within 2 to 14 weeks of delivery | Logistic regression model |
| Tiki et al., 2020 | Ethiopia | West Shoa zone, Oromia regional state (urban) | 862 pregnant women | (mean age (± SD) = 28.41 ± 5.9) | Assess the prevalence of depression and associated factors among pregnant mothers in the community setting | Primary data | Cluster sampling | Second and third trimester | Logistic regression model |
| Phukuta et al., 2020 | South Africa | Levai Mbatha Community | 227 postpartum women | 18 and above | Assess prevalence and associated factors associated with postnatal depression | Primary data | Consecutive | Antenatal | Chi-square and logistic regression model |
| Belete et al., 2020 | Ethiopia | Bahir Dar Town (urban) | 988 postpartum women | 18 and above | Assess the proportion of postpartum depression and associated factors among postnatal women in care in public health facilities | Primary data | Multi-stage sampling | 6–8 weeks postpartum | Logistic regression model |
| Mokwena et al., 2020 | South Africa | sub district in Tshwane | 406 postnatal women | 18 - 45 (mean = 28 (SD = 5.91)) | Determine the prevalence of, as well as factors associated with, postnatal depression in a sample of clinic attendees | Primary data | Simple random | Within a year of delivery | Logistic regression model |
| Kassaw et al., 2020 | Ethiopia | Dilla, Ethiopia (urban) | 178 pregnant women | mean = 28 (SD = 5.6)) | Determine the magnitude and factors associated with general anxiety disorder among pregnant women | Primary data | Consecutive | Any trimester | Logistic regression model |
| LeMasters et al., 2020 | Malawi | two urban and three rural areas in Lilongwe | 73 women living with HIV | NR | Determine the experiences of postnatal depression among women living with HIV (WLWH) in Malawi. | Primary data | NR | Prenatal/postnatal | Narrative analysis |
| Umuziga et al., 2020 | Rwanda | Eastern province (rural) | 165 women | 15 and above | Explore the prevalence of symptoms of perinatal depression and anxiety in Rwanda, and its factors | Primary data | Systematic sampling | Second and third trimester of pregnancy, and up to 1 year postnatal | Logistic regression |
| Govender et al., 2020 | South Africa | Ugu, KwaZulu-Natal Province (rural) | 326 adolescent postpartum mothers | 13-19 | Estimate the prevalence of depression and its associated risk factors among pregnant and postpartum adolescents | Primary data | Convenient | Prenatal/postnatal | Logistic regression |
| Ola et al., 2011 | Nigeria | Ikeja, Lagos (Urban) | 178 women | Age range not reported; mean = 30.28 (4.4) | Assess the magnitude and predictors of common mental disorder | Primary data | Consecutive | NR | Logistic regression |
| Shitu et al., 2021 | Ethiopia | Gurage zone, Addis Ababa (urban) | 343 pregnant mothers | age range not reported; Mean = 27.3 (SD ± 5.6)). | Assess its magnitude and associated factors among pregnant women attending antenatal clinics | Primary data | Systematic | Any trimester | Logistic regression |
| Mbatha et al., 2020 | South Africa | rural KwaZulu-Natal | 386 postpartum women | age range not reported; mean = 29 (4.4) | Determine the prevalence of and clinical and obstetric risk factors for postnatal depression symptoms among HIV positive women accessing rural facilities | Primary data | Convenient | Within 1-12 weeks of live delivery | Logistic regression |
| Ngocho et al., 2019 | Tanzania | Kilimanjaro region | 200 pregnant women | 18 years and above | Determine prevalence of depression and anxiety and identify factors associated with these common mental health disorders among HIV-infected pregnant women | Secondary data analysis of longitudinal data | NR | Second or third trimester | Logistic regression |
| Oladeji et al., 2019 | Nigeria | Ibadan (urban) | 9,352 pregnant women | NR | Examine difference between adolescents and adults in the occurrence and correlates of perinatal depression | Secondary data analysis using data from cluster randomized controlled trial | NR | Any trimester | t-test, ANOVA and linear regression |
| Lodebo et al., 2020 | Ethiopia | West Badewacho woreda (urban) | 541 pregnant women | 18-49 (mean= 27.28 (SD = 4.8)) | Assess the prevalence of antenatal depression and associated factors among pregnant women | Primary data | Multistage random | Any trimester | Logistic regression |
| Arach et al., 2020 | Uganda | rural district of Lira, Northern Uganda | 1,789 postpartum women | 12-47 (mean (± SD) was 25 (± 7) | Examine the association between perinatal death and postpartum depression among women in Lira district, Northern Uganda. | Secondary analysis of data from a community-randomized trial | NR | Women who gave birth at 28 weeks of gestation or more and were available 50 days postpartum | Generalized estimation equation with poison link |
| Pellowski et al., 2019 | South Africa | Not reported | 831 postpartum women | NR | Determine the trajectories of depressive symptoms over time in South Africa | Secondary analysis of cohort study data | NR | Second trimester | Multinomial logistic regressions |
| Modjadji et al., 2020 | South Africa | Thembisile Hani local municipality, located in Nkangala District of the Mpumalanga Province | 228 postpartum women | Age range not reported; mean age (SD) 28 ± 7. | Assess/screen for postnatal depression and determine the risk factors among postpartum women in selected community health centers. | Primary data | Purposive | Postpartum within 12 weeks of delivery | Logistic regression model |
| Mbarak et al., 2019 | Tanzania | Dar es Salaam community | 390 postpartum mothers | age range not reported; mean = 29.2 ± 6.28 | Determine the magnitude and risk factors for postpartum women among diagnosed with pre-eclampsia or eclampsia at Muhimbili National Hospital. | Primary data | Convenient | Postpartum women who were diagnosed with pre-eclampsia or eclampsia during pregnancy; delivered and attended postnatal clinic | Logistic regression model |
| Goweda et al., 2020 | Egypt | Suez governorate | 237 postpartum women | 17-45; mean (SD) = 27.9 ± 5.9 | Estimate the prevalence of postpartum depression and to identify the associated risk factors. | Primary data | NR | Postpartum women seeking care within 4 - 12 weeks following delivery, or attending child vaccination or family planning clinic | Fisher exact and Chi-square tests |
| Saeed et al., 2019 | Ghana | Mankuma community in Bole district (rural) | 244 mothers with under-five children | 18 and above (mean age = 28.7 ± 6.29) | Determine the prevalence of depression and identify its determinants in mothers of children under 5 years | Primary data | Simple random | Mothers with children under- 5 years and attending child welfare clinic | Chi-square and logistic regression model |
| Dlamini et al., 2019 | Eswatini | Manzini | 114 postpartum women | 18 and above | Describe the prevalence and factors associated with postpartum depression among women seeking postnatal and child welfare services | Primary data | Convenient | Mothers seeking postnatal or child welfare services and between 7 days and 6 weeks of postpartum | Logistic regression model |
| Belete et al., 2019 | Ethiopia | Aneded woreda in Amhara Region | 342 pregnant women | 15 and above; median age was 24 with IQR of 18-28 | Assess the prevalence of antenatal depression and factors associated with antenatal depression among pregnant women | Primary data | Simple random | All registered pregnant women in community health information system | Logistic regression model |
| Woldetsadik et al., 2019 | Ethiopia | (Robe, Goba and Ginnir) of Bale zone South-East Ethiopia | 743 pregnant women | age range not reported; mean ± SD = 27.3 ± 5.2 | Assess the prevalence of CMD and its determinants among pregnant women in Southeast Ethiopia | Primary data | Multistage | All registered pregnant women at any trimester | Logistic regression |
| Necho et al., 2020 | Ethiopia | Dessie town, South Wollo zone, Amhara region, Northeast Ethiopia | 378 postnatal women | 15–49 (mean = 29.85 (SD = 6.39) | Determine the magnitude of postpartum depression and its correlates | Primary data | Systematic | Within 4 weeks of delivery | Logistic regression model |
| Davies al., 2016 | South Africa | urban township, Cape Town | 84 postpartum women | 18 years or older | Examine the experiences and explanations of depression amongst Xhosa-speaking pregnant women and mothers | Primary data | Purposive | Any trimester pregnancy and postpartum within 1 year of delivery | Thematic analysis |
| Stewart et al., 2014 | Malawi | Mangochi District Hospital, Mangochi, Malawi (rural) | 583 pregnant women | mean age 25.14 (SD +/- 6.22) | Examine the prevalence and factors associated with common mental disorders | Primary data | Convenient | Antenatal | Negative binomial regression and logistic regression models |
| Heyningen et al., 2016 | South Africa | urban area of Cape Town, South Africa | 376 pregnant women | 18 and above | Investigate the prevalence of risk factors for antenatal depression among women living in adversity in a low-resource, urban setting in Cape Town | Primary data | Systematic | Antenatal | Logistic regression model |
| Manikkam et al., 2012 | South Africa | KwaZulu-Natal Province (urban) | 390 pregnant women | NR | Determine the prevalence and risk factors associated with depression among pregnant women attending depression at KwaZulu | Primary data | Consecutive | Antenatal | Logistic regression model |
| Bindt et al., 2012 | Ghana and Cote d'Ivoire | Kumasi (Ghana) and Abobo community (Cote d'Ivoire) | 1,090 pregnant women (299 from Ghana and 731 from Coˆte d’Ivoire) | 18-46 (mean = 29.6 (SD) = 4.88) for Ghana and 28.4 (5.58) for Cote d’Ivoire) | Assess the impact of communicable and non-communicable diseases on infant development | Primary data | Consecutive | Any trimester | Linear regression |
| Wemakor et al., 2016 | Ghana | Bilpeila Health Centre, Tamale (rural) | 384 pregnant women | 15-45 (mean = 27.9 (± 8.2) | Examine the association between maternal depression and stunting in mother-child pairs attending Child Welfare Clinic (CWC) in Northern Ghana | Primary data | Census | All women (aged 15–45 years) with apparently healthy children under 5 years old seeking CWC services at the Bilpiela Health Centre or its 2 outreach posts | Logistic regression |
| Gold et al., 2014 | Ghana | Kumasi (urban) | 183 postpartum women | 18 years or above (mean = 28) | Describe the prevalence of and risk factors for depression in a high-risk population of mothers of ill newborns in Ghana | Primary data | Convenient | All mothers of infants hospitalized in the mother to baby unit | Fisher exact test |
| Kaida et al., 2014 | Uganda | Mbarara District (rural) | 477 women living with HIV | 18-49 (median age was 32) IQR: 27–37) | Measure and compare depression symptom severity during non–pregnancy-related, pregnant, and postpartum periods among HIV-infected women initiating ART in rural Uganda | Secondary data analysis | NR | Women seeking HIV care | Linear regression using generalized estimating equations and multivariable GEE logistic regression model |
| Nydoo et al., 2017 | South Africa | KwaZulu-Natal Province (urban) | 102 pregnant women (62 HIV uninfected and 40 HIV-infected) | 17-40; mean ± SD = 25 ± 5.30 | Compare depressive scores between newly diagnosed HIV-infected and HIV- uninfected pregnant women | Primary data | Census | Second or third trimester | t-test and Spearman's correlation coefficient |
| Khalifa et al., 2016 | Sudan | Khartoum state | 238 pregnant women | NR | Explore the factors associated with postnatal depression (PND) at 3 months postpartum in a sample of Sudanese women in Khartoum state | Primary data | NR | Second or third trimester | Logistic regression model |
| Kakyo et al., 2012 | Uganda | Kabarole (rural) | 202 postnatal mothers | 16 and above (mean (S.D) = 24 (4.33)) | Explore the factors associated with postpartum depressive symptoms among women living in a rural district | Primary data | Census | Postpartum | Pearson correlation statistics and point biserial |
| Shamu et al., 2016 | Zimbabwe | Harare | 842 postnatal women | 18-49 | Investigate the association between postnatal depression and suicidal ideation with emotional, physical and sexual IPV experienced by women during pregnancy | Primary data | Consecutive | Postpartum | Logistic regression model |
| Tsai et al., 2016 | South Africa | three townships surrounding Cape Town (urban) | 1,238 pregnant women | NR | Estimate the association between IPV and depression symptom | Secondary analysis of longitudinal study data | Consecutive | Any trimester | Quantile regression and logistic regression models |
| Anderson et al., 2014 | Kenya | Southern Nyanza Province | 135 HIV positive pregnant women | 18 and above; mean = (24.26 (SD = 4.87) | Examine associations between linkage to HIV care, postpartum depression, and internalized stigma in a population with a high risk of depression | Secondary analysis of longitudinal study data | Census | Antenatal | Generalized estimating equations (GEE) using binary logistic models |
| Koen et al., 2017 | South Africa | TC Newman and Mbekweni in the Drakenstein sub-district in Paarl, Western Cape | 111 postpartum mothers | 18 and above; median = 25 (IQR = 22-31) | Investigate the association between maternal posttraumatic stress disorder (PTSD) and infant development in a South African birth cohort | Secondary data from the Drakenstein Child Health Study | Systematic | Within 20–28 weeks gestation | Linear regression models |
| Nöthling et al., 2013 | South Africa | Cape Town | 70 HIV-infected postpartum mothers | mean = 28.8; range 16–64 | Investigate whether maternal postpartum trauma exposure and PTSD were risk factors for child behaviour problems | Primary data | Systematic | Within 12 months of delivery | Linear regression models |
| Boakye-Yiadom et al., 2015 | Ghana | Tamale West | 154 pregnant women | Range: 17–42; mean (SD = 27.9 ± 5.8) | Assess the prevalence of stress and anxiety, as well as the association that exists between stress/anxiety and sociodemographic characteristics, among pregnant women | Primary data | NR | Any trimester | Chi-square and unpaired t-test |
| Madeghe et al., 2016 | Kenya | Kariobangi North and Kariobangi South in Nairobi (urban) | 200 postpartum women | 17–39 | Examine the effects of postpartum depression on infant feeding practices and malnutrition among women in an urban low-income settlement | Primary | Census | Postpartum | Logistic regression |
| Roos et al. | South Africa | Western Cape | 110 pregnant women | 18 and above (mean (SD) = 25.4 (5.38)) | Examine whether temperament and character, trait anxiety, resilience and social support predicted distress and anxiety symptoms in pregnancy | Primary data | Simple random | 20 weeks of gestation | ANOVA and linear regression |
| Ayele et al., 2016 | Ethiopia | Gondar town (urban) | 388 pregnant women | 14 and above. (median = 25; IQR: 28.75–22.0) | Determine the prevalence and identify associated factors for antenatal depression | Primary data | Systematic random | 16-39 weeks gestational age | Logistic regression |
| Bekele et al., 2017 | Ethiopia | Addis Ababa (urban) | 753 pregnant women | Mean (SD) 23.4± 4.75; range: 15-42 | Determine the prevalence and factors associated with MD during pregnancy among ANC attendees at Saint Paul’s Hospital (SPH) | Primary data | Systematic random | Third trimester | Logistic regression |
| Lillie et al., 2020 | Ghana | rural Northern Ghana | 374 pregnant women | 16-50; mean = 27.0 (6.8) | Describes the prevalence of depression among rural pregnant women, participating in a maternal and child health program, in Ghana, and examines associated risk factors for depression | Secondary analysis of data from a cluster randomized control trial | NR | Any trimester | Modified Poisson and generalized estimating equations (GEE) model |
| Bisetegn et al., 2016 | Ethiopia | Debretabor Town (urban) | 527 pregnant women | 17-39; mean = 27.5 (SD ± 4.95) | Determine the prevalence and associated factors with antenatal depression. | Primary data | Cluster sampling | Any trimester | Logistic regression |
| Koen et al., 2016 | South Africa | peri-urban community outside Cape Town | 544 mother-infant dyads | 18 and above | Investigate the prevalence and risk factors for maternal trauma and posttraumatic stress disorder (PTSD), and their association with adverse birth outcomes in the Drakenstein Child Health Study, a South African birth cohort study | Secondary analysis of longitudinal study data | Systematic | 20 to 28 weeks of gestation | Linear and logistic regression models |
| Yator et al., 2017 | Kenya | Nairobi (urban) | 123 postpartum women | 18–50 years (mean = 31 (SD = 5.2)) | Determine the prevalence and severity of postpartum depression (PPD) amongst women living with HIV and to further understand the impact of stigma and other psychosocial factors in 123 women living with HIV attending Prevention of Mother to Child transmission (PMTCT) clinic | Primary data | NR | Postpartum | Logistic regression |
| Azale et al., 2016 | Ethiopia | Sodo district, Gurage Zone | 385 postpartum women | Mean = 28.8 (SD = 5.23) | Determine the proportion of women with PPD who sought help form a health facility and the associated factors | Primary data | Multistage sampling | Postpartum | Logistic regression model |
| Wassif et al., 2019 | Egypt | Qaliubeya governorate | 500 postpartum women | Mean ± SD (range) 25.6 ± 5.07 (18–40) | Measure the prevalence of postpartum depression and/or anxiety among females and to explore the underlying factors of these disorders and find if progesterone level has a role | Primary data | Multistage random sampling | Postpartum | Logistic regression |
| Garman et al., 2019 | South Africa | Cape Town settlements (peri-urban) | 446 pregnant women | 18 years or above (mean = 26) | Identify trajectories of depressive symptoms and its predictors among low-income perinatal women in South Africa and its association of trajectories with child outcomes | Secondary analysis of cluster randomized controlled trial (RCT) data | Randomization | Any trimester | Latent class growth analysis (LCGA) and linear regression |
| Nyamukoho et al., 2019 | Zimbabwe | Chitungwiza City Council (urban) | 198 HIV positive pregnant women | 18 and above (mean (SD) = 26.6 (4.5)) | Determine the factors associated with depression in HIV positive pregnant women in Zimbabwe | Primary data | Simple random | First trimester | Logistic regression model |
| Duko et al., 2019 | Ethiopia | Hawassa city (Urban) | 317 pregnant women | 15 and above (mean (SD) = 23.82 ± (6.65)) | Assess the prevalence and factors associated with depression among pregnant women in public health institutions, Hawassa, Ethiopia | Primary data | Systematic sampling | Any trimester | Logistic regression model |
| Belay et al., 2019 | Ethiopia | Wondo Genet district (peri-urban) | 589 pregnant women | 16 and 45 (mean = 25) | Measure the prevalence of IPV and depression during pregnancy and assess the association between IPV and depression and other determinants. | Primary data | Purposive and consecutive | 25–34 weeks of gestational age | Logistic regression model |
| Garman et al., 2019 | South Africa | Khayelitsha, a peri-urban informal settlement in Cape Town | 384 pregnant women | 18 and above (mean (SD) = 27 (5.56)) | Identify trajectories of perinatal depressive symptoms and their predictors among women living in a low-resource setting in South Africa, and who present with a risk of depression during pregnancy | Secondary analysis of cluster randomized controlled trial (RCT) data | Randomization | First or second trimester | Logistic regression model |
| Kerie et al., 2018 | Ethiopia | Southwestern Ethiopia | 422 postpartum women | 15 and above (mean= 26.06 (± 5.79) | Determine the prevalence and associated factors of postpartum depression among mothers who gave birth within the last 12 months among hospitals of Southwest Ethiopia | Primary data | Systematic sampling | Postpartum | Logistic regression model |
| Harrington et al., 2020 | Malawi | Lilongwe | 725 HIV positive pregnant women | 16 and above (median = 29 (IQR) 24–33)) | Describe the prevalence and factors associated with antenatal depression among Malawian women enrolled in Option B+ | Secondary analysis of cohort study data | Census | Any trimester | log binomial regression model |
| Belete et al., 2019 | Ethiopia | Bahir Dar (subcity) | 988 postpartum mothers | 18 and above | Assess the prevalence and associated factors of suicidal behavior (suicidal ideation, plan or suicide attempt) in postpartum mothers | Primary data | Multistage | Postpartum | Logistic regression model |
| Fantahun et al., 2018 | Ethiopia | Addis Ababa (urban) | 618 postpartum mothers | 15 and above (mean (SD) = 28.05(5.0)) | Assess the prevalence and factors associated with postpartum depression among postpartum mothers attending public health centers in Addis Ababa, Ethiopia | Primary data | Multistage | Postpartum | Logistic regression model |
| Mokhele et al., 2019 | South Africa | Tshwane, Ekurhuleni and Johannesburg Metropolitan districts in the Gauteng Province (peri-urban) | 1,151 postpartum women | 18 and above (median (IQR) =29 (25–33) | Measure the prevalence of PPD comparing postpartum HIV-1 infected women with pre-pregnancy HIV care experience, newly diagnosed (in latest pregnancy) HIV-1 infected women and HIV negative women, and to identify predictors of major PPD among these women in a peri-urban clinic in South Africa | Secondary analysis of a randomized controlled trial (RCT) data | Convenient | Postpartum | Ordinal logistic regression |
| Woldetensay et al., 2018 | Ethiopia | Woliso, Tiro-Afeta and Gomma districts in Southwestern Ethiopia | 4,680 pregnant women | Age range not reported; median age (IQR) = 26 (22, 30) | Describe the prevalence of prenatal depressive symptoms and whether it is associated with maternal nutrition, intimate partner violence and social support among pregnant women in rural Ethiopia | Secondary analysis of cohort study data | Convenient | 12-32 weeks of gestation | Logistic regression model |
| Chingono et al., 2021 | Zimbabwe | Harare, Zimbabwe | 93 adolescent (expectant) mothers | 16-19 | Explore whether and how participation in a self-help group intervention affected vulnerable young mothers’ experiences and perceptions of mental health stressors | Primary data | NR | Antenatal/postnatal | Basic frequencies and cross tabulations |
| Lelisho et al., 2022 | Ethiopia | Kembata Tembaro, in the Southern Ethiopia zone, | 423 women | Mean = 31.09 (SD = 3.72) | Determine the prevalence of generalized anxiety disorder symptoms during COVID-19, and its associated factor among mothers attending perinatal service in the study area during the pandemic through the application of ordinal logistics regression | Primary data | Random sampling | Perinatal | Ordinal logistic regression model |
| Silverman et al., 2021 | South Africa | Tzaneen in Mopani District, Limpopo Province, South Africa | 224 women | 18 and above | Characterize rates and patterns of common mental disorder symptoms over the postpartum period among women in rural areas surrounding Tzaneen, a city located in the Limpopo Province of South Africa, and to identify associated risk factor | Secondary analysis of baseline data nested within a larger, 2-year long child development intervention trial | Convenient | Postnatal | Linear regression models |
| Kariuki et al., 2022 | Kenya | Nairobi | 575 postnatal mothers | 18 and above | Investigate the Postnatal depression risk factors among mothers attending Lang’ata and Riruta Maternal and Child Health Clinics (MCH) in the slums, Nairobi | Primary data | NR | Postnatal | Multivariate linear regression models |
| Soyemi et al., 2022 | Nigeria | Abeokuta | 285 pregnant women each for first and third trimester | above 18years | Compare the prevalence and correlates of depression in the first and third trimesters of pregnancy and to determine the relationship between quality of life and depressive disorder | Primary data | Systematic random | Prenatal | NR |
| Osborn et al., 2021 | Kenya | Nairobi | 824 pregnant women living with HIV | above 14years | Evaluate the prevalence and correlates of depression, as well as the population attributable risk percent (PAR %) of those correlates on depression among pregnant WLWH in Kenya | Primary data | NR | Prenatal | Generalized estimating equation models (GEE) with a Poisson link and independent correlation structure |
| Prospective/longitudinal cohort studies | | | | | | | | | |
| Adeoye et al., 2022 | Nigeria | Ibadan, the city in Oyo State, South-Western, Nigeria | 1,745 pregnant women ≤ 20 weeks’ gestation,  aged ≥ 18 years, without severe medical complications. | 18 and above (mean = 29.9) | Assess prevalence of depression, associated factors and perinatal outcomes of antepartum depression (APD) among pregnant women | Primary data | NR | ≤ 20 weeks of gestation | Chi-square and t-tests. Logistic and Poisson regression models |
| Ngene et al., 2021 | South Africa | Not reported | 50 pregnant women with severe preeclampsia and 90 normotensive pregnant women | Age range (23 - 28) | Determine if any maternal pre-delivery soluble correlate with either perceived stress scale (PSS) or verbal numeric rating scale (VNRS) pain scores. | Primary data | NR | Postpartum | Mean, standard deviation, interquartile range and Spearman's correlation |
| Larsen et al., 2022 | Kenya | Nairobi and Nyanza region | 825 participants | 28 (IQR: 23–30) | Examine longitudinal patterns and cofactors of depressive symptoms among pregnant and postpartum women living with HIV (WLWH) | Primary data | NR | Antenatal/postnatal | Generalized estimating equation models (GEE) with a Poisson link and independent correlation structure |
| Bitew et al., 2017 | Ethiopia | Rural (Sodo district) | 1251 antenatal women | NR | Examine whether antenatal depressive symptoms are associated with use of maternal health care services | Primary data | NR | Second and third trimesters | Multivariate logistic regression |
| Khalifa et al., 2018 | Sudan | Khartoum state. | 300 postpartum mothers | 28 | Investigate the change in screening status and in severity of depression and distress symptoms between three and eight months postpartum | Primary data | Random sampling | Second or third trimester | Correlations |
| Wong et al., 2017 | South africa | Urban (Cape Town) | 628 HIV-infected pregnant women | > 18: mean age for younger women (ages 18–24) was 22 and 30 for older women (≥ 25) | Assess depression, alcohol use, and stigma in younger versus older HIV-infected pregnant women initiating antiretroviral therapy in Cape Town, South Africa | Secondary analysis | NR | Antenatal | Multivariable logistic regression |
| Yotebieng et al., 2017 | Democratic Republic of Congo | 85 clinics in Kinshasa | 433 newly-diagnosed HIV-infected women, ≤ 32 weeks pregnant | Median age among depressed 30 (26, 34); not depressed 29 (25, 34) | Assess the association between prenatal depression and 1) loss of follow-up or 2) uptake of PMTCT services | Primary data | NR | Antenatal through postpartum | Logistic regression |
| Bitew et al., 2017 | Ethiopia | Rural (Sodo district) | 1240 antenatal women | NR | Investigate whether antenatal depressive symptoms predict perinatal complications in a rural Ethiopia setting | Primary data | NR | Second and third trimesters | Multivariate logistic regression |
| Tuthill et al., 2017 | South africa | Kwazulu Natal | 68 South African women living with HIV in their third trimester (28–42 weeks) of pregnancy | 18+ | Identify levels of perinatal depression among women living with HIV in South Africa and examine the relationship of perinatal depression on infant feeding outcomes. | Primary data | NR | Prenatal and Postpartum | Multivariate logistic regression |
| Rogathi et al., 2017 | Tanzania | Kilimanjaro Region, Tanzania | 1,013 pregnant women of less than 24 weeks gestation attending ANC | > 18 | Assess the relationship between IPV and PPD among women attending antenatal services in Tanzania | Primary data | NR | Antenatal | Logistic regression |
| Pingo et al., 2017 | South africa | Eastern metropole of Cape Town | 57 postpartum women | 25.1 (6.4) | Evaluate the frequency of, and factors associated with, postpartum hypomania (PPH) and postpartum depression (PPD) in a South African sample | Primary data | NA | Postpartum | Logistic regression |
| Ongeri et al., 2018 | Kenya | outpatient waiting areas of maternal and child health (MCH) clinics associated with two major public hospitals | 171 pregnant, between ages 18 and 49 years, and in their third trimester of pregnancy | Depression: 25.5 (22.8, 28.0); no depression: 25.0 (22.5, 29.0) | Estimate the prevalence, incidence and correlates of significant postpartum depressive symptoms in Kenyan women | Primary data | NR | Antenatal/postnatal | Logistic regression |
| Rodriguez et al., 2018 | South africa | Rural | 69 mother-infant dyads | 29 ± 5 | Examine the impact of depression in HIV-infected women in rural SA on infant development | Primary data | NA | Antenatal and postnatal | Logistic regression |
| Wall et al., 2017 | Tanzania | Antenatal clinics in the Ilemela and Nyamagana districts of Mwanza, Tanzania | 212 women with 32 weeks’ gestational age or less at the time of enrolment based on women’s last menstrual period | median (range) 26 (16–44) | Assess Factors associated with pregnancy related anxiety in Tanzanian women: a cross-sectional study | Primary data | Systematic | Antenatal and postnatal | Multiple linear regression |
| Peltzer et al., 2018 | South africa | public primary care in rural South Africa | 681 HIV-infected prenatal women between 8 and 24 weeks of gestation | 28.3 (SD = 5.7); range of 18–46 | Assess the longitudinal prevalence of prenatal and postnatal depression and associated factors among HIV-infected women in rural South Africa | Primary data | Consecutive (drawn from drawn an ongoing longitudinal PMTCT RCT) | Antenatal and postnatal | Logistic regression |
| Guo et al., 2014 | Ghana and Cote dvoire | Urban | 577 pregnant women from the 3rd trimester in pregnancy to 2 years postpartum residing within a distance of ≤ 5 km from the study hospitals | 29.1 (5.5) | Assess mental health-related determinants of parenting stress among urban mothers of young children–results from a birth-cohort study in Ghana and Côte d’Ivoire | Primary data | NR | Antenatal and postnatal | Generalized estimating equation (GEE) |
| Weobong et al., 2015 | Ghana | Rural/periurban communities within the Kintampo Health Research Centre study area | 13,360 mothers who had a live singleton birth | NR | Assess the impact of probable depression in the immediate postnatal period on subsequent infant mortality and morbidity. | Primary data | NR | Postpartum | Logistic regression |
| Acheampong et al., 2021 | Ghana | Bekwai Municipal | 360 pregnant women with severe and mild depression | 18-47(mean = 27.5) | Compare the risk of developing adverse outcomes between pregnant women with moderate-severe depression and those who had mild depression | Primary data | NR | Second trimester | Chi-square and log-binomial regression mode |
| Abrahams et al., 2022 | South Africa | Cape Town | 859 postnatal women | NR | Examine the association between socio-economic adversities at baseline and CMD prevalence and worse mental health would be associated with higher food insecurity | Primary data | NR | Within 6 months of postpartum | Multivariable (multilevel) regression analysis |
| Okafor et al., 2021 | South Africa | Drakenstein subdistrict of the Western Cape Province, South Africa (periurban) | 981 women | 18 or older (mean = 27) | Investigate the longitudinal association between emotional, physical or sexual IPV and depression among South African women. | Primary data | Data collected at the second trimester of pregnancy, 6 months, 12 months, and 18 months postpartum | Second trimester of pregnancy | Chi-square test and pooled logistic regression (GEE Model) |
| Malaju et al., 2022 | Ethiopia | South Gondar zone, Northwest Ethiopia. | 775 postpartum women | 15 and above (26.41 + 4.25) | Investigate the trajectories of Post traumatic stress disorder (PTSD) symptoms and mediating relationships of variables associated with it among postpartum women | Primary data | Simple random sampling | Postpartum women within 6-, 12- and 18-weeks following delivery | Confirmatory factor analysis (CFA), Structural Equation Model (SEM), and multinomial logistic regression |
| Okunola et al., 2021 | Nigeria | Ekiti State (urban) | 272 pregnant women | 15-49 (mean = 31 (5.28)) | Evaluate the relationship between antenatal depression and postpartum depression and predictors of postpartum depression | Primary data | Consecutive | 34-36 weeks of pregnancy and after delivery | Chi-square, fisher exact test and logistic regression |
| Beyene et al., 2021 | Ethiopia | Debre Tabor and Woreta towns (urban) | 933 pregnant women | 15 and above (29.6 +/- 9.45 SD) | Examine the independent effect of antenatal depression on newborn birth weight in an urban community in Northwest Ethiopia | systematic sampling techniques | Systematic | Gestational ages from 16 to 34 weeks | Logistic regression model |
| Knettel et al., 2020 | Tanzania | Kilimanjaro region (six urban and three rural facilities) | 200 HIV-positive pregnant women | 18-44 (mean = 30 (SD = 6) | Examine patterns and predictors of suicidal ideation among women living with HIV in antenatal care | Primary data | Convenient | second or third trimester | Binary logistic regression |
| Dadi et al.i, 2020 | Ethiopia | Gondar town (urban) | 940 pregnant women | 18 and above; mean (±SD) age was 26.5 (± 4.5) | Explore the causal mechanisms underlying antenatal depression | Primary data | Consecutive | Second and third trimesters | Structural equation model |
| Bitew et al., 2019 | Ethiopia | Sodo district (rural) | 1,240 postnatal women (356 with and 884 without antenatal depressive symptoms) | 15 and above (mean = 26.8) | Identify independent predictors of persistence and incidence of postnatal depressive symptoms | Primary data | Systematic | second or third trimester and 4–12 weeks after childbirth | Poisson regression with robust standard errors |
| Mnisi et al., 2019 | South Africa | Pretoria, South Africa (urban). | 2,671 postpartum mothers | 17-41; mean = 27.96 | Determine the prevalence and identify risk factors for postnatal depression in mothers whose babies were admitted to the neonatal unit | Primary data | Systematic | Between 26 and 42 weeks' gestation (preterm) | Logistic regression model |
| Weobong et al., 2014 | Ghana | Kintampo (rural) | 20,679 pregnant women | NR | Examine the associations of antenatal depression (AND) with survival of the baby | Primary data | Census | All women of reproductive age were visited at home every 4-weeks by a local field worker | Logistic regression model |
| Kaaya et al., 2016 | Tanzania | Dar es Salaam (urban) | 699 pregnant women living with HIV | 15 and above | Examine the relationship between maternal depression and infant malnutrition among women living with HIV | Primary data | Convenient | HIV-positive pregnant women less than 27 weeks of gestation | Generalized estimation equation with exchangeable working structure |
| Mochache et al., 2018 | Kenya | Nairobi (urban) | 255 pregnant women | 18-40 | Determine whether antenatal depression is associated with preterm delivery in a low resource hospital sample from suburbs of Nairobi. | Primary data | Systematic | First and second trimesters | log binomial regression analysis |
| Guo al., 2014 | Ghana and Cote d'Ivoire | Kumasi (Ghana), and the Abobo Community Hospital in Abidjan (Côte d’Ivoire) | 577 pregnant women | 18 and above (mean=29.1 (5.5)) | Determine the association between ante- and postnatal depression and anxiety on parenting stress | Primary data | Consecutive | Third trimester | Generalized estimating equation (GEE) |
| Bindt et al., 2013 | Ghana and Cote d'Ivoire | Kumasi (Ghana), and the Abobo Community Hospital in Abidjan (Côte d’Ivoire) | 719 pregnant women | 18 and above (mean = 29.1 (5.5)) | Explore the association between antenatal depression and anxiety symptoms and birth outcomes in a low-obstetric risk sample of mother/child dyads | Primary data | Consecutive | Third trimester | Logistic regression model |
| Okronipa et al., 2012 | Ghana | Manya and Yilo Krobo districts, Eastern region (rural) | 245 pregnant women | 18-48; mean = 28.5 ± 0.3 | Examine the incidence and days ill with diarrhea among infants of HIV positive (HIV-P), HIV negative (HIV- N), and unknown HIV status (HIV-U) women, and determined if symptoms of maternal postnatal depression (PND) modulated the risk of diarrhea | Primary | Convenient | Any trimester | Logistic regression |
| Stewart et al., 2018 | Malawi | Mangochi District (rural) | 1,006 pregnant women | Mean (SD) = 25.3 (6.1) | Investigate whether antenatal depression is associated with shorter duration of pregnancy and reduced newborn size in rural Malawi | Primary data | Census | Second or third trimester | Linear regression |
| Pobee et al., 2022 | Ghana | Cape coast, Ghana | 116 pregnant women | 18-38 years | Determine the reliability of the questionnaires used to assess psychosocial outcomes, then establish the prevalence estimate of depressive and anxiety symptoms and low QoL throughout pregnancy in Cape Coast, Ghana | Primary data | Multistage | Prenatal | Exploratory factor analyses |
| Naude et al., 2022 | south Africa | peri-urban area outside Cape Town, South Africa | 255 pregnant women | 18 and above | Investigate if antenatal maternal depressive symptoms are associated with elevated inflammatory cytokines in mothers and in their children in early life, and further, to determine whether these elevated immune markers play a mediating role in the relationship between maternal depressive symptoms in pregnancy on neurodevelopmental outcomes | Primary data | Convenient | Prenatal to 2 years after birth | SPSS, Wilcoxon t-test, Pearson's chi-squared test, multiple linesr regression, linear mixed models |
| Shuffrey et al., 2022 | South Africa | Western cape province of south Africa | 600 maternal-infant dyads | 16 or older at the time of consent, and a gestational age between 6 and 40 weeks | Determine if prenatal maternal depression and state or trait anxiety were associated with child social-emotional problems or cognitive development at approximately 3 years of age in a South African cohort from the Western Cape. | Primary data | Convenient | Prenatal to 3 years after birth | Linear regression models |
| Mandell et al., 2022 | South Africa | Mpumalanga rural setting | 217 pregnant women living with HIV | 18 and above | Examine BP, depression, and suicidal ideation among pregnant WHIV in rural South Africa | Primary data | Convenient | Prenatal | Univariate stastictics, t-tests, chi-square tests, SPSS |
| Boateng et al., 2022 | Kenya | seven rural, peri-urban, and rural areas (Kisumu, Macalder, Migori, Nyahera,Nyamaraga, Ongo, Rongo) in the former Nyanza region, Kenya | 371 pregnant women within first 7 months of pregnancy | 18-38 | Understand the temporal relationship between time variant risk factors for depression, i.e. food and water insecurity 2. To assess how these factors potentially interacted with HIV to increase depressive symptomatology | Primary data | Quota | Postnatal | Linear regressions, univariate analyses |
| Randomized Controlled Trial studies | | | | | | | | | |
| Gureje et al., 2022 | Nigeria | four urban and five rural commuties in Oyo state, Nigeria | 242 pregnant women (intervention: 141; Control: 101) with foetal gestational age of <36 weeks and speaking Yoruba | age < 20, mean = 18 | Effect of intervention delivered by frontline maternal care providers to improve outcome and parenting skills among adolescents with perinatal depression in Nigeria | Primary data | Block randomization | < 36 weeks of gestational age | Multivariable mixed-effect regression models to |
| Rodriguez et al., 2018 | South africa | Rural (Nkangala districts in Mpumalanga province) | 681 women living with HIV | Mean age: 28.3 (SD = 5.7) | Assess perinatal correlates of suicidal ideation among women living with HIV (WLHIV) in rural South Africa | Primary data | NR | Antenatal and postnatal | Multinomial logistic regression |
| Gordon et al., 2021 | South Africa | Peri-urban township of Cape Town | 1,111 pregnant women | age group not reported; mean = 26.5 (5.3) | Examine the impact of depressed mood on child outcomes | Primary data | Randomization | Antenatal | Multilevel fixed-effects logistic regression model |

| **Note:** | DHS: Demographic and Health Surveys. |
| --- | --- |
|  | MICS: Multiple Indicator Cluster Surveys. |
|  | RCT: Randomized Controlled Trial. |
|  | SPA: Service Provision Assessment. |
